# Co-Designing and Evaluating a Multimodal Digital Application to Enable People With Dementia to Self-Report Quality of Life Patient-Reported Outcome Measures: Co-Design Study and Summative Evaluation

**DOI:** 10.2196/87565

**Published:** 2026-04-01

**Authors:** David Kernaghan, Kieren Egan, Marilyn Lennon, Roma Maguire

**Affiliations:** 1 Digital Health and Wellness Research Group, Computer Information Science, University of Strathclyde, Glasgow, Scotland, United Kingdom; 2 School of Health in Social Sciences, University of Edinburgh, Doorway 6, Medical School, Teviot PlaceEdinburgh, Scotland, EH8 9AG, United Kingdom

**Keywords:** dementia, patient-reported outcomes, co-design, ePROMs, multimodal, accessibility, mobile phone, electronic patient-reported outcome measure

## Abstract

**Background:**

Worldwide, there are an estimated 55 million people living with dementia, with an estimated cost to society of US $1.3 trillion a year. These numbers are predicted to rise, with the dementia population doubling every 20 years, reaching an estimated 152 million by 2050. There is currently no cure for dementia, with the condition having a significant impact on people’s independence and quality of life (QoL). It is therefore vital that people living with dementia are given the support that helps them to manage these impacts and optimizes their QoL. To do this, a more personalized and detailed understanding of a patient’s daily life is needed. Patient-reported outcome measures (PROMs) have long been associated with significant benefits in other domains, though the use of PROMs in routine dementia care is lacking. Factors such as platform design, literacy, language proficiency, and physical and mental capability can all severely impact the ability of people living with dementia to complete PROMs routinely.

**Objective:**

This study aims to co-design and evaluate a novel multimodal digital application to enable people living with dementia to regularly self-report QoL PROMs. The research questions were (1) What features, questions, and modalities do people living with dementia prefer when interacting with a digital application that enables them to self-report QoL PROMs? (2) What are the design considerations and specifications for a digital application to enable people living with dementia to self-report QoL data via PROMs?

**Methods:**

People living with dementia, informal caregivers (ICs), and health care professionals (HCPs) participated in iterative co-design workshops and a final summative evaluation to co-design a multimodal digital application from initial concepts to a functional prototype. Prototypes were presented using cognitive walkthroughs and think-aloud protocols, and data were analyzed following framework analysis using interaction design and features voted for by participants using MoSCoW (Must Have, Should Have, Could Have, Won't Have).

**Results:**

A total of 25 participants took part in this study (people living with dementia=9, ICs=4, and HCPs=12) developing and evaluating a total of 34 prototypes into a single final functional multimodal prototype that can collect PROMs using text, visuals, and voice.

**Conclusions:**

A functional prototype for a novel digital application was developed that enables people living with dementia to regularly self-report QoL PROMs, which was then successfully evaluated by people living with dementia, ICs, and HCPs. The prototype was co-designed with direct involvement from people living with dementia during every stage of development, and this is one of the first studies to evaluate perceptions from key stakeholders (including people living with dementia, ICs, and HCPs) regarding the use of electronic patient-reported outcome measures for dementia in routine care.

## Introduction

### Background

Worldwide, there are an estimated 55 million people living with dementia (people living with dementia), with the condition now the 7th leading cause of mortality [1]. These numbers are predicted to rise, with the dementia population doubling every 20 years, reaching an estimated 152 million by 2050 [2]. There is currently no cure for dementia, with the condition having a significant impact on people’s independence and quality of life (QoL) [3]. It is therefore vital that people living with dementia are given the support to manage these impacts and optimize their QoL with health and care services that focus on delivering person-centered care. The nature of dementia requires health care professionals (HCP) to recognize and monitor individuals’ changing needs throughout the disease’s progression and adjust care and practices accordingly while preserving the individual’s personhood [4,5].

One such tool to monitor an individual’s changing needs is patient-reported outcome measures (PROMs). These are questionnaires used to measure subjective outcomes relating to a patient’s health, QoL, or functional status associated with health care or treatment, reported directly by patients without interpretation from HCP or other parties [6]. Traditional PROMs take the form of paper questionnaires, though electronic patient-reported outcome measures (ePROMs), digital PROMs that can be completed remotely on various internet-connected devices and collated automatically, offer an efficient method of data collection with a comparable level of response compared to traditional methods [7] as well as reducing times and associated costs [8].

PROMs and ePROMs are increasingly recognized as an important tool for fostering clinician-patient communication, informing direct care decisions, and improving the quality of care [9,10]. PROMs continue to see greater use in routine care delivery to assess patients’ experiences, evaluate outcomes, and encourage shared decision-making [11], with many patients welcoming the use of PROMs and believing they should be used more routinely [12]. Despite this, implementation of PROMs is lacking, with significant barriers including patient inability to complete PROMs, the perceived irrelevance of PROMs and their lack of value to the patient, as well as difficulty using electronic devices to complete PROMs [13]. This difficulty in completing PROMs is compounded for individuals with cognitive impairments, where the content, layout, and procedures of the PROM can pose barriers if PROMs are implemented without cognitive accessible designs in mind [14,15].

One solution would be to develop PROMs that are better designed and more accessible for people living with dementia in their day-to-day environment. While such PROMs have existed for decades [16], their usage is almost exclusively limited to research studies [17]. QoL measures are often used as proxies instead of dementia-specific PROMs [18], though these often do not capture benefits and issues specific and meaningful to people living with dementia [19] and are not designed with cognitive accessibility in mind [14]. This issue is compounded by the lack of high-quality QoL PROMs that are validated for use in a home environment [20].

Despite the challenges, PROMs could provide a largely unrealized potential for advocating the needs and measuring the QoL of people living with dementia in a scalable manner [17]. It is therefore vital that the voice of the people living with dementia is enabled to be heard, as it is “a fundamental right of all patients and individuals living with long-term conditions to be included in the decisions and be in control of their own care and support” [21].

### Aims and Objectives

This study aims to co-design and evaluate a novel digital application to enable people living with dementia to self-report QoL PROMs. To achieve this, the following research questions were answered: (1) What features, questions, and modalities do people living with dementia prefer when interacting with a digital application that enables them to self-report QoL PROMs? (2) What are the design considerations and specifications for a digital application to enable people living with dementia to self-report QoL data via PROMs?

## Methods

### Study Design

This iterative qualitative study invited people living with dementia and informal caregivers (ICs) to co-design and test prototypes for a novel application during 4 cycles of online workshops that developed a final functional prototype (FFP) before a final summative evaluation cycle involved all stakeholders to review and evaluate the FFP. Before this study, a short patient and public involvement and engagement phase allowed stakeholders to voice their expectations and recommendations for the prototype, which were used to develop the initial prototypes. People living with dementia and ICs were then invited to review and refine these prototypes during the co-design phase, where they directly contributed to the look, design, and functionality of the application, as well as the type of PROM questions asked, and the modality of interaction. All stakeholders were then invited to evaluate the FFP during the summative evaluation phase.

Questions and responses used in the prototypes were originally taken from the quality of life in Alzheimer disease (QoL-AD) PROM questionnaire [22] using the “participant version” and the verbal questions from “instructions for the interviewer” [23]. QoL-AD was chosen on recommendation from the health care and charity professional partners, and for having validated questions for both text-based and voice-based modalities. Early prototypes used these questions and responses verbatim. The prototypes were designed to be web-based for use on computers, laptops, and large-screen tablets following the preferences of participants during initial patient and public involvement, engagement research, and prior literature research [15,24-29].

### Co-Design Phase

**Figure 1.**
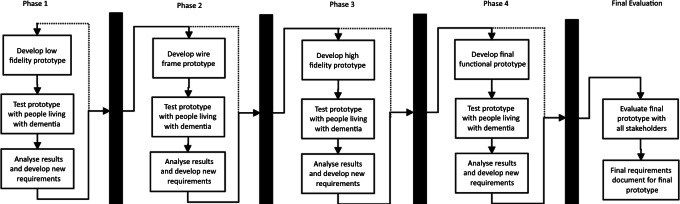
Prototype development phases. WFP: wireframe prototype.

We present four interlinked iterative cycles, with each cycle consisting of 3 agile sprints involving development, testing, and analysis (Figure 1). Cycle 1 focused on the development, testing, and analysis of a low-fidelity prototype (LFP). Cycle 2 developed the LFP into a wireframe prototype (WFP). Cycle 3 improved WFP into high-fidelity prototypes (HFP) with cycle 4 culminating in an FFP. Between each workshop, participants voted on several features they felt should be prioritized for the next prototype.

### Summative Evaluation

A final summative evaluation phase concluded this study, with all stakeholders evaluating the functional prototype for its feasibility, usability, and utility.

During cycles 1‐3, author DK performed cognitive walkthroughs [30] with participants, detailing the features and differences of each prototype concept to participants who provided their opinions and recommendations on how they can be improved. During cycle 4, DK led participants in a think-aloud protocol [31] using the FFP while relaying their experiences and opinions. All workshops were recorded and overseen by an HCP and an experienced researcher (KE or RM) who ensured the needs of participants were always met. Participants were also offered regular breaks during workshops and were reminded that if they were struggling with cognitive or emotional issues, they should inform the experienced researchers. Audio from the workshops was fully transcribed into text using a combination of Microsoft Stream (Microsoft Corp) and human transcription by DK. These transcriptions were analyzed by DK along with notes taken by KE and RM using NVivo (Lumivero) with major themes extracted using framework analysis [32] following the 5 dimensions of interaction design to determine what features should be improved, removed, or added [33]. These themes were then presented to participants via an online poll where they voted on what features they want prioritized using the MoSCoW (Must Have, Should Have, Could Have, Won't Have) prioritization method [34]. This informed the sprint product backlog for the next iterative cycle, where the prototype was redeveloped into a higher fidelity version and presented to participants for usability testing during the next workshop. During cycle 1, general feedback collected in notes written by the researchers was used for analysis, as well as the results of the MoSCoW. This was due to unforeseen technical issues that resulted in no usable recordings or transcripts for cycle 1.

### Ethical Considerations

Ethical approval was granted by the National Health Service (NHS) Health Research Authority (IRAS Project ID: 273039). Participants received no compensation. Informed consent was collected from all participants who were provided a participant information sheet detailing the study details including their right to withdraw at any time, as well as a consent form that could be completed via written consent or recorded verbal consent. All identifiable data collected was anonymized.

## Results

### Recruitment

A total of 25 individual participants took part in the entire study. Of these, 7 took part in the co-design phase and 20 in the summative evaluation. Two participants took part in both phases.

### Co-Design Phase

A total of 7 participants (people living with dementia and IC) were recruited from various charities and NHS partners across Scotland to take part in 4 online co-design workshops conducted between October 2021 and May 2022 (Table 1). For participants who completed the demographic questionnaire (n=5), a total of 4 identified themselves as “somewhat or very confident” using technology, with 1 participant “somewhat unconfident.” Participants identified themselves as having access to a wide variety of commercial technology, including laptop computers (80%, 4/5), e-readers (80%, 4/5), tablets (60%, 3/5), smartphones (60%, 3/5), smart speakers (20%, 1/5), and smartwatches (20%, 1/5).

**Table 1.** Participant details for both phases.

Characteristics	Co-design phase (N=7)		Evaluation phase (N=20)	
Gender, n (%)				
Woman	4/7 (57)		10/20 (50)	
Man	3/7 (43)		10/20 (50)	
Race, n (%)				
White	7/7 (100)		18/20 (90)	
Asian	N/A^a^		2/20 (10)	
Role type, n (%)				
Person with dementia	5/7 (71)		6/20 (30)^b^	
Informal caregiver	2/7 (29)		2/20 (10)	
Health care professionals, n (%)			12/20 (60)^c^	
Manager or lead	—^d^		3/20 (15)	
Doctor or consultant	—		3/20 (15)	
Nurse	—		2/20 (10)	
Not stated	—		4/20 (20)	

a N/A: not applicable.

b Two previously participated in the co-design phase.

c Two took part in the MoSCoW (Must Have, Should Have, Could Have, Won't Have) but could not attend the workshops.

d Not available.

### Summative Evaluation

For the summative evaluation phase, a total of 20 participants took part. This included 12 HCPs recruited via the NHS and 8 participants (people living with dementia and IC) recruited via Alzheimer’s Scotland (including 2 returning from the co-design phase), who took part in the summative evaluation cycle over 3 separate workshops in May 2022.

**Figure 2.**
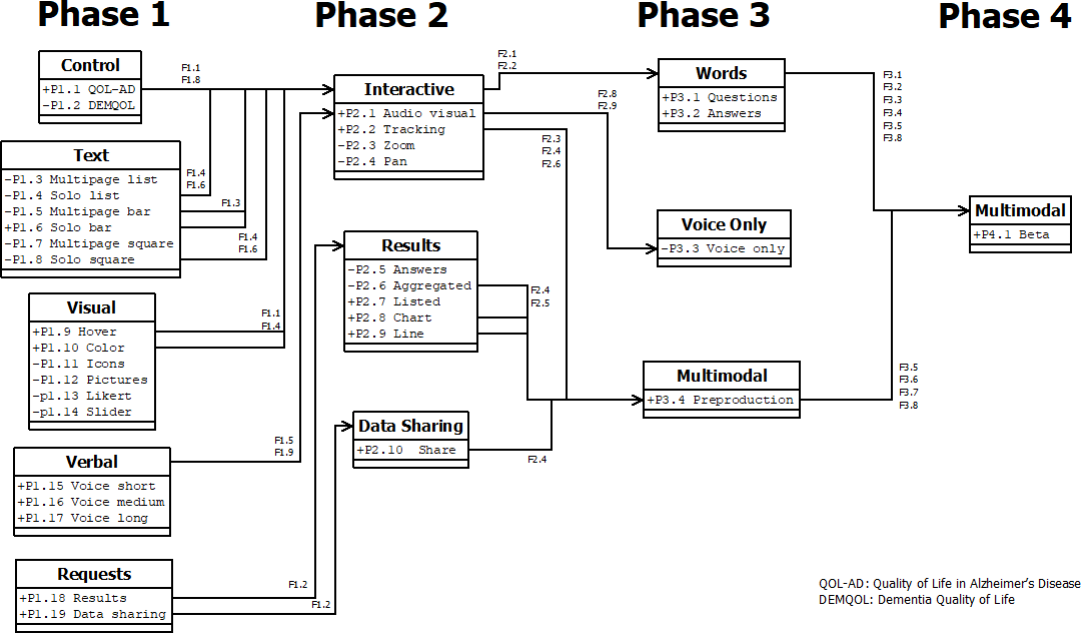
Prototype development class chart. DEMQOL: dementia quality of life; QOL-AD: quality of life in Alzheimer’s disease.

A total of 34 prototypes were developed over the course of 4 iterative co-design workshop cycles (Figure 2, Multimedia Appendix 1). Features from these prototypes were combined and refined over the course of the workshops into an FFP (Figure S38 in Multimedia Appendix 2). The results of the MoSCoW for each phase are displayed and ranked in Tables 2-5.

**Table 2.** Workshop 1 MoSCoW^a^ results.

ID	Features	MoSCoW score	Will not include	Priority
F1.1	Larger font and bolder fonts	1.95	0	High
F1.2	Results page for participants to see their own responses	1.8	1	High
F1.3	Options displayed in boxes	1.55	1	High
F1.4	Automatically move to the next page	1.5	0	High
F1.5	Actor’s voice recording	1.45	3	Moderate
F1.6	One question on each page	1.35	0	Moderate
F1.7	All questions on 1 page	1	2	Low
F1.8	More detailed questions and explanations	0.85	1	Low
F1.9	Visual prompt with verbal questions	0.85	1	Low

a MoSCoW: Must Have, Should Have, Could Have, Won't Have.

**Table 3.** Workshop 2 MoSCoW^a^ results.

ID	Features	MoSCoW score	Will not include	Priority
F2.1	Adjust some of the words to be more inclusive (such as changing “spouse“ to “partner,“ “decreased“ to “reduced,“ “stayed the same“ to “no change“)	2.35	0	Very high
F2.2	Make the questions less ambiguous and more relevant to me	1.8	0	High
F2.3	Show my previous answers after I submit them, and let me change them	1.7	0	High
F2.4	Option to turn colors on or off	1.6	0	High
F2.5	Have a drop-down menu for the results page that goes into more detail	1.6	0	High
F2.6	Show reminders of what kind of questions have been asked (such as ”mood“ instead of “question 3”)	1.1	0	Moderate
F2.7	Option to add notes to your answers so I can provide more details	1.1	0	Moderate
F2.8	Let me answer using my voice instead of touching the screen	0.6	1	Low
F2.9	Have the results be spoken using audio instead of reading	0.6	2	Low
F2.10	Use animations and screen transitions between questions	0.3	2	None

a MoSCoW: Must Have, Should Have, Could Have, Won't Have.

**Table 4.** Workshop 3 MoSCoW^a^ results.

ID	Features	MoSCoW score	Will not include	Priority
F3.1	Shorten and simplify longer questions	1.8	1	High
F3.2	Remove terms such as “lately“ and be more precise with time frames	1.8	1	High
F3.3	Mark sensitive questions (such as marriage or friends) as optional and not asked if not relevant.	1.35	1	Moderate
F3.4	Change unclear questions that start with “how about“	1.1	1	Moderate
F3.5	Add comment boxes to questions to add our own notes	1.05	2	Moderate
F3.6	Allow us to add people we can share the data with ourselves	0.75	2	Low
F3.7	Allow us to answer using just our voice	0.25	1	Low
F3.8	Provide an option to skip questions	0.15	0	Low
F3.9	A voice-only option that will run on a device such as Siri (Amazon.com, Inc) or Amazon Echo (Amazon.com, Inc)	0	0	None

a MoSCoW: Must Have, Should Have, Could Have, Won't Have.

**Table 5.** Workshop 4 MoSCoW^a^ Results.

ID	Features	MoSCoW score	Will not include	Priority
F4.1	A button at the end to end the session	2.6	0	Very high
F4.2	Questions automatically move to the next when answered	2.3	0	Very high
F4.3	A comment section to add your own notes	2	0	High
F4.4	A button to print out the results	1.6	0	High
F4.5	A mobile version of the application	1.2	1	Moderate
F4.6	Thicker lines and larger fonts are used on the line graph	1	1	Moderate
F4.7	More visible buttons on the ”share with“ page	0.85	1	Low
F4.8	Voice feature that reads out questions and allows you to answer with your voice	0.6	2	Low

a MoSCoW: Must Have, Should Have, Could Have, Won't Have.

The FFP was designed to be used by people living with dementia in a home environment, either independently or supported by IC. The prototype is accessible via a web link that is sent to the user and presents users with a home screen with options to answer the questionnaire, view their previous results, or adjust settings such as the color display or voice modality. The questionnaire asks 12 questions modified from QoL-AD, with the option to have them read out using a digital voice. Once the questionnaire is completed, users are presented with their responses and the opportunity to update them. Users can then choose who to share this information with. Once completed, users are returned to the main menu with the option to review their results in multiple charts and over various periods of time.

### Cycle 1

During cycle 1, which focused on the development, testing, and analysis of LFPs during online workshops, the highest priority features related to the visual dimension of interaction design, with 3 of the top priority features relating to that dimension (F1.1, F1.2, and F1.3), with the time dimension and space dimension also scoring moderately. “Larger font and bolder fonts” took the highest priority for users, which reflects one of the main talking points during the session. Users complained that the font display was far too small. Prototypes had to be magnified 150% of normal size to allow participants to clearly see and read. Participants were keen to have questions automatically move to the next once answered (F1.4), with 1 question displayed per page (F1.6). While participants were interested in actors’ voice recordings being used in future prototypes (F1.5), the feasibility of this during the prototyping phase was limited and out of scope for this study. Another feature heavily discussed in the workshop that scored highly was related to ownership of data and participants being able to see their own results (F1.2), as well as the ability to take ownership of this data and be able to show it to relevant people and professionals. No prototypes had been developed for these features, so prototypes for this were implemented in cycle 2 (P2.5-P2.10).

### Cycle 2

For cycle 2, which aimed to develop the LFPs into WFPs, the MoSCoW response had the highest priority features related to the word dimension (F2.1 and F2.2), in particular, the choice of words and phrasing used in the questions. Terminology was the highest priority issue raised (F2.1), with many participants taking umbrage at the use of “marriage” on the results page. While this topic was not discussed as heavily during the workshop as the issue of ambiguity, it scored significantly higher in the MoSCoW feedback. The next priority feature regarding questions was their ambiguity and lack of relevance (F2.2) with much criticism raised against the format of the questions and the predefined response options. Additionally, many participants voiced confusion about why a question about physical health was being asked as part of a dementia PROM and felt the question in its current form was irrelevant to them and their dementia. Participants queried the use of the word “lately,” criticizing that with dementia, many found it hard to gauge periods of time coherently.

### Ambiguity

Much criticism was raised against the format of the questions and the predefined response options. Participants found the phrasing of the questions to be inappropriately positive when discussing a topic of dementia, especially with 1 response being “excellent.” Participants also noted that the combination of some questions and responses was grammatically incorrect. The first question using the term “physical health” was deemed to be ambiguous, with participants unsure whether it was asking about their physical activity, their physical health overall, specifically regarding their dementia, or how their physical health is being treated. Additionally, 1 participant voiced confusion about why a question about physical health was being asked as part of a dementia PROM and felt the question in its current form was irrelevant to them and their dementia. Participants also criticized the use of the word “lately” in some of the questions, noting that with dementia, many found it hard to gauge periods of time coherently, and the vague use of “lately” made them unsure on whether they were thinking of today, the past week, or since the last questionnaire. Many stated that their response could fluctuate significantly over any given period of time and that they would prefer questions that were asked in the moment.

*I I'm not, I'm not. Trying to be obstructive, but I would have thought the great majority of people who have dementia don't feel good about it [dementia]. Don't feel. Don't feel fair about it [Dementia].*

[PWD3]

*Yeah, I, I mean I just. Think that you come along. You are a stranger. You ask me how I am about my physical health, yeah. And erm. It just does not. It's, it is just the word. Best word is ambiguous… this it is not a good question.*

[PWD3]

*Are you asking about dementia? or how [is] the rest of me?*

[PWD5]

*I need to be honest and say I think it's a silly question for somebody with dementia. The reason I’ve been diagnosed with dementia is that I have a poor memory.*

[PWD2]

### Relevance

Participants also raised issues with the relevance of the fourth QoL-AD question, which asked about the users’ living situation [23]. The question was intended to gauge users’ satisfaction with their home environment, though the phrasing and use of “now” was interpreted by participants as indicating that their home environment had changed. Many found this inappropriate for a frequent questionnaire and did not foresee it changing bar from significant changes in circumstances. Participants felt the phrasing of the question needed to be changed, or to ask a prequestion asking if their living situation had changed before asking their response. Further, 1 participant also questioned the relevance of questions asking about users’ memories and found it to be “a silly question for somebody with dementia.”

***PWD5:** That's not likely to change week from week. I’d have thought, yeah. So, I don't quite see the point of it. In a weekly questionnaire.*

***PWD2:** I see what PWD5 means, mean. my situation wouldn't change at all. unless the worst happened.*

***PWD2:** You can get into a care home or something. But it's not on a weekly basis. It's not something that I think would necessarily change.*

***PWD3:** I feel the addition of the word “now” yeah. Uhm? Alters the meaning of the rest of the question ahead. How do you feel about the place you live now? It is implying that.*

***PWD5:** You changed?*

### Terminology

Participants were vocal about the terminology used in the questions. Even with prototypes (P2.1, P2.2, P2.3, and P2.4) developed to showcase features relating to other dimensions (visual, space, or time), participants would often cycle back to criticize the terminology of the questions. Participants did not like, for example, the use of “marriage” on the results page. Participants correctly pointed out that marriage would exclude participants who are unwed, in civil partnerships, or in other partnerships, with many participants requesting a change to “partner.”

***PWD2:** I struggle with the word marriage. Can that be change to partnership?*

***PWD5:** That should actually.*

***PWD2:** cause I'm in a same sex relationship.*

***PWD5:** Yeah, it should be. Yeah, it should be changed.*

***PWD2:** So, it doesn't really accommodate me.*

***PWD2:** I’d like to switch spouse for partner. And also have an option for no one.*

***PWD5:** I think you’d have to.*

### Misread and Tonally Incorrect

Additional requests regarding the word dimension were changing words that could be misread or were seen as tonally incorrect, such as “decreased,” which could be misread as “deceased,” and “excellent” being replaced for being far too positive a response.

***PWD5:*** *With my dementia, I saw deceased.*

***PWD4:*** *Oh.*

***PWD5:*** *So, I think, if possible, could you change that.*

***PWD4:*** *Yeah.*

***PWD2:** Deceased! ha-ha.*

***PWD5:*** *Yeah, you do get funnier things with my dementia.*

### Cycle 3

The top priority during cycle 3, which aimed to improve by changing WFPs into HFPs, was the word dimension, with 3 of the top 4 prioritized themes (F3.1, F3.2, and F3.4) belonging to this dimension. A significant amount of priority was also set for the behavior dimension (F3.3, F3.5, and F3.6). Matching the framework analysis results for workshop 3, MoSCoW results recognized shortening and simplifying the longer questions (F3.1) as well as updating the terminology that asks about time (F3.2) as being the top priority. Participants also wanted to see grammar updated to remove the use of questions starting with “how about” for greater clarity (F3.4). Regarding question sensitivity, participants had a significant preference for unnecessary or sensitive questions to not be shown at all to participants (F3.3) rather than just the option to skip (F3.9). While participants were open to the idea of being able to skip questions entirely in the workshop, this was a rather low priority. Participants also showed interest in providing greater input using the application. A free-text comment box for some questions would allow them to provide additional context along with their answers (F3.5), and the ability to nominate people with whom the data are shared directly into the application (F3.6) was also pitched. As with previous MoSCoW results, voice modality features were of limited priority (F3.7 and F3.9).

### Complexity of Questions

A particular barrier highlighted was the length and complexity of questions. Most QoL-AD questions [23] were deemed far too long and multifaceted to read, or the questions were asked in a way that did not align with the responses available. Several questions were noted to be too long, especially those that posed multiple facets for a single question (Q2 and Q3). Some questions were deemed too large in their scope for participants to consider (Q6), and questions where a yes or no answer seemed more appropriate than a 4-point scale (Q4). Further, 1 participant found the short headings used above each question to be enough and easy to read at a glance. Finally, participants noted that some questions (Q6 and Q7) could be combined as the overlap between family and partners would be hard for them to separate.

*I think it’s difficult because there are 3 answers[Q3], it’s not as, not as focused as I’d expect it to be?*

[PWD3]

*I think questions 6 and the other [Q7] can be put together. Or maybe have parts to put the family members with different options but I don’t think it needs to be separate questions.*

[PWD4]

*Yes, I’d be able to answer it, but I also think that’s there’s too much there, the headings enough [Q9]*

[PWD4]

### Terminology

Another area of significant comment related to the frequent use of certain terminology. As raised in cycle 2 (F2.2), participants disliked questions that asked users to consider a period of time, especially with the term “lately,” which they found imprecise. This was once again raised in cycle 3 when reviewing all the questions with participants, noting the term was “difficult” to gauge what period of time the question wanted you to consider. Participants also commented on questions starting with “how about” (Q4, Q5, and Q6). Participant PWD2 noted the grammar was incorrect, which made some questions unanswerable with the responses available, and participants much preferred a more personal question, such as “how do you feel,” which was easier to interpret. While these terminology issues may appear minor, participants appeared to be fixated on these issues, with half of the entire workshop’s runtime spent discussing the questions.

*I think the use of the term lately is perhaps difficult, does that mean yesterday and today or in the last fortnight?*

[PWD2]

*Depends if you’re thinking about the present time? And is it like a regular question so you monitor it?*

[IC2]

***PWD2:** I’m not sure about “how about your memory” as that’s not really a question.*

***PWD2:** Again, I don’t want to be difficult but again I don’t know what the question is. It starts with how about? What is the answer to “how about”?*

*Yeah, I’d probably bin it [QoL-AD] to be honest, sorry to be so straight forward.*

[PWD2]

### Sensitivity

One theme that arose was how many of the questions asked about participants’ personal lives that could be insensitive, upsetting, or rude. Participants queried if people who were widowed, had no family, or did not have friends would be asked these questions. PWD2 also asked if information about a person’s situation could be collected before the initial setup of the app, so these questions can be omitted from the questionnaire on a user-by-user basis. Participants were also keen on the option to skip questions.

*What do you do if you’re answering the questions, and your spouse is dead?*

[PWD3]

***PWD2:*** *I think the option to skip would be useful. Because of my situation I don’t have children and I’m an only child and have no nieces or nephews so be able to skip the question without refusing me to continue like the census [ The Scottish census which was discussed prior to recording] did.*

***PWD2:*** *And I think PWD3’s point that if someone’s been widowed, if we are gathering that type of information before we are starting the questionnaire process then a flag that says don’t ask questions about marriage or a partner if they’ve been widowed or bereaved, that would be helpful.*

*I’m aware I’m talking as if I am “tommy no mates” but there are some people who see themselves as not having a circle of friends and being asked this question could be a trigger for some emotion around the fact, I am “tommy no mates” and I have got nobody.*

[PWD2]

### Free Text Input

Participants also showed interest in having additional input options when using the FFP. Numerous times when discussing the questions, participants requested a free-text comment box to be added so they could provide additional context along with their answers.

*If it was on a comment, so instead of putting a yes or no, then a bit for a comment where you can put your own wee bit in.*

[PWD4]

*I think there is an assumption that people can. Maybe the question should be are you able to do things that you enjoy and then a comment box for you to list what they are rather than again this “how about.” Sorry I was an English teacher so start a question with how about? How about what? What’s the answer to how about?*

[PWD2]

### Data Sharing and Autonomy

A consistent theme that arose again during cycle 3 was regarding data autonomy. This was raised in cycle 1 with participants wanting to access their results and in cycle 2 with participants requesting greater control of who the data is shared with. In cycle 3, participants were keen on the ability to easily print results on headed paper so they could take this with them to appointments and to show to their HCPs. Participants also wished for the feature to nominate their own people that the data would be shared with.

*Would you be able to make it so you could go to the doctor or hospital and take that [printed results]?*

[PWD4]

***PWD2:*** *Would you be able to add someone to that list?*

***PWD4:*** *I think it would be useful to be able to add it yourself. Yeah.*

***PWD3:*** *I agree with the discussion and thinks it’s all fine.*

### Voice

As with cycles 1 and 2, the use of voice was of very limited interest to participants, with the voice-only prototype (P3.5) receiving universal dislike. Participants unanimously agreed that if voice is to be implemented, it must be accompanied by a visual or textual prompt that displays the question asked and the answer responses, as many said they would struggle to remember these using voice only.

***PWD4:*** *I think even with the verbal one, although the voice is saying Good, Fair, Etcetera, I think its need to be on the page because depending on people’s memory, they might not remember what the answers were spoken.*

***DK:*** *So, you definitely prefer a visual experience or a visual prompt alongside the audio?*

***PWD4:*** *Yes*

***PWD2:*** *Or instead of!*

### Cycle 4

For the final co-design workshops, which aimed to culminate the HFPs into an FFP, time was the most prioritized dimension (F4.1 and F4.2), followed by behavior (F4.3 and F4.4). Participants wished to see a definitive end to the application with an “end session” button that would close it when completed, reassuring them that it is finished. Due to the way the FFP (P4.1) handled data in its database, an earlier feature, which automatically moved to the next page once an option was selected (F1.4), did not function correctly. While the feature did not come up in discussion during the cycle 4 workshop, it was included in the MoSCoW survey, where participants gave significant priority for it to be reinstated (F4.2). The MoSCoW results also showed priority for a comment box to be added (F4.3) so users could add additional context to their responses, as well as a print option being added inside the application (F4.4). Participants also requested some minor visual adjustments, though these were of relatively low priority. Finally, participants had moderate interest in the app being developed for mobile (F4.5), though once again, they had little interest in implementing voice modalities (F4.8).

### End Session

Some users were not aware of what to do once they had completed the questionnaire and reviewed their results. While the webpage can be closed at any time, the lack of a prompt to do this caused minor confusion with 1 participant. It was therefore requested that the inclusion of an “end session” button to be displayed at the end.

*I’ve done that, I’m now back at the screen that says, “your dementia app. Thank you for submitting the questionnaire.” There's nothing that tells me how to close it.*

[PWD2]

*The only thing for me is at this stage is a button to see close or end session.*

[PWD2]

### Free Text Input

Participants were concerned that some of the questions did not provide enough context, with just 4 response options. For example, if the user had an argument earlier that day, this could affect their response to how they feel about friends or family. Adding an optional free-text input at the end of the questionnaire would allow users to include such context. This theme was previously raised in cycle 3 (F3.5), but after further discussion, it was decided that a single comment at the end of the questionnaire was better than a per-question basis.

***PWD2:*** *I think some of the questions I'm just a bit concerned that. Your answer is going to be specific to the day that you do the questionnaire. … And if you've had a row with somebody first thing in the morning, and you do this in the afternoon, yeah. That might affect how you answer.*

***PWD4:*** *You know, even if they have [had an argument] a comments box somewhere, yeah, you know to put in comments if you needed too, you know?*

*[in response to being asked “any other features”]*

***PWD4:*** *And the comments box!*

***IC2:*** *And it would be optional, wouldn’t it? So, you comment if you wish?*

### Frequency

Participants were keen to have the option to choose the frequency of the questionnaire. Most participants seemed to favor monthly delivery for the PROM. One participant did request once a week, and this feature was previously raised as it would be better for building up a routine and make it harder to forget.

***PWD2:*** *I think making it once a month would be more likely to encourage people to use it than once a week.*

***PWD4:*** *Hmmm, yeah, I think you should have the option to do it weekly because I think if it was. Monthly. And. I might forget what I did, yeah? Or even 2 weeks ago? Yeah no. So, I would need to do weekly.*

### Data Autonomy and Ownership

One participant inquired if their data could be saved and with the functionality to print their results. While this feature does exist natively for most web browsers, the participant was keen to have this feature built into the application for easy use. This feature was previously raised in cycle 3 and would be useful to have where participants wished to have results printed on headed paper that they could take with them to appointments and share with their HCPs.

*Would that be printable?*

[PWD2]

*Would I also be able to save that?*

[PWD2]

### Mobile

Participants were indifferent to the prototype being developed for mobile devices. Some thought it may be useful to have as an option, though most agreed that a mobile screen is far too small to comfortably use for this purpose.

***DK:*** *Anyone be interested in seeing it on a smaller device like a mobile phone?*

***IC2:** Urmmmmmm.*

***PWD2:*** *I’m not sure on that.*

***PWD4:*** *Well, I would like the option.*

***IC2:*** *I think too small. There's just not, not manageable.*

***PWD2:*** *I think the options there I’d probably not take the option. Yeah, if it's there, it's there.*

*No, I think phone things are too small.*

[PWD5]

### Setup

Participants were confident in their ability to set up the prototype themselves, as well as being in control of who the data is shared with. Participants who could access the prototype were able to access the questionnaire and all the features independently and quickly. One participant mentioned they would feel uncomfortable with having someone else, such as an HCP setting up the questionnaire, especially regarding questions about finance that they would not like to have raised with an HCP.

***IC2:** Set it up myself, I think.*

***PWD4:** I think so.*

***PWD2:** I think so too, yes.*

*I think that is probably quite a good idea, I wouldn’t want my doctor to know too much about my personal life.*

[PWD5]

### Summative Evaluation

A total of 3 workshops were conducted as part of the summative evaluation workshops involving 2 major stakeholder groups, the first 2 workshops involved HCPs from NHS (n=12, Table 6), and the final workshop involved people living with dementia and IC from the charity Alzheimer’s Scotland (n=8, Table 7).

**Table 6.** NHS^a^ MoSCoW^b^ results.

ID	Themes	Theme covered	MoSCoW score	Will not include	Priority
F5.1	Implementation of a text message or SMS-based system to send the questionnaire	Behavior	2.7	0	Very high
F5.2	Further research into mobile-friendly and application versions	Space	1.85	0	High
F5.3	How to encourage user participation, especially during certain periods of their journey	Behavior	1.65	0	High
F5.4	Expand testing to those with later and more severe stages of dementia	Behavior	1.5	1	Moderate
F5.5	Rebuild inside existing systems using the established and approved tools	Space	1.45	0	Moderate
F5.6	Offer greater breakdown and filter options for how data is displayed to all users	Visuals	1.25	1	Moderate
F5.7	Look into interoperability and how the data can be shared and stored in established systems	Space	1.15	0	Moderate
F5.8	Avoid changes to questions and restore the original QoL-AD^c^/DEMQOL^d^ questions	Word	0.75	2	Low
F5.9	Further research into voice systems, such as Alexa (Amazon.com, Inc) devices	Word	0.7	0	Low

a NHS: National Health Service.

b MoSCoW: MoSCoW (Must Have, Should Have, Could Have, Won't Have) prioritization method.

c QoL-AD: quality of life in Alzheimer disease questionnaire.

d DEMQOL: DEMQOL (dementia quality of life) questionnaire.

**Table 7.** Alzheimer’s Scotland MoSCoW^a^ results.

ID	Themes	Theme covered	MoSCoW score	Will not include	Priority
F6.1	Test the reliability and validity of the questionnaire and results	Word	High	0	2.45
F6.2	Review the use of certain words and colors for better usability	Word	High	0	2.4
F6.3	Ensure GPs^b^ and HCPs^c^ are on board with using this system	Behavior	High	0	2.3
F6.4	Further research into mobile-friendly and application versions	Space	Moderate	0	1.75
F6.5	How to encourage user participation, especially during certain periods of their journey	Behavior	Moderate	1	1.7
F6.6	Further research into voice systems, such as Alexa devices	Space	Low	1	0.7
F6.7	Avoid changes to questions and restore the original questionnaire	Word	Low	1	0.65

a MoSCoW: MoSCoW (Must Have, Should Have, Could Have, Won't Have) prioritization method.

b GP: general practitioner.

c HCP: health care professional.

### NHS Evaluation

Analysis from the NHS evaluation showed the highest priority features related to the space or behavior dimension, with the top 5 features all relating to those dimensions (F5.1, F5.2, F5.3, F5.4, and F5.5). The behavior dimension was also heavily prioritized in cycle 4. The space dimension, on the other hand, proved to be a notable dimension for HCP, who took particular interest in the feasibility and technology being implemented, particularly in relation to their existing systems and processes.

The delivery method was the top priority for HCP, with them inquiring if the application could be delivered via text or SMS message (F5.1) and whether further research could be performed to develop a mobile-friendly version of the prototype (F5.2).

*Just about the email aspect. I'm not sure if that many of the patients use email. Sort of experience from clinics. I think sometimes the carers do but even that’s quite rare and the patients, I think seldom use email.*

[HCP2]

*Did you look at like phone numbers to send the link? … Instead of the email, yeah, use their phone numbers and you know. And then they can access it.*

[HCP4]

*I think we are quite used to receiving text messages from the NHS because I am I just got 1 today… so I think patients might be quite used to that because they're already getting that from their surgeries. And I think that might be something to explore if there is a better way of linking it to your questionnaire.*

[HCP1]

The next prioritized features involved investigating ways to encourage user participation throughout the different stages of their treatment journey (F5.3), including expanding the testing of the prototype to people with later and more severe stages of dementia (F5.4) to understand the app’s usability at stages where problematic behavior is more prevalent. They also recommended revisiting icons and images, as other patients may find the use of visual aids helps those who are less digitally skilled or have a more severe diagnosis of dementia to better interpret the questions being asked.

*I think it's a really good way to measure somebody's response to medication to care treatments and things like that on a week-by-week basis. So, I actually think it's really good and the simplicity of it for people who are obviously coping in public they appear, but they're a very stressed period in their life… I would actually incorporate that into my assessments, review feedback to the consultant.*

[HCP3]

*I guess it's about kind of envisioning which group would be using it? And then the frequency kind of comes into lawful purpose. I mean it is almost at the early stages of dementia, so if you think of immediately post diagnosis, is this about trying to kind of get some sort of early detection of somebody's QoL starting to sail off?... Actually, if a dementia outreach team were seeing somebody for quite an intense period of work, more frequently might be useful.*

[HCP1]

*You could change it depending on the execution so like the crisis team, so they might want the weekly because their engagement is good, yeah? Very short term but doctors would be monthly, or you know something along those lines to get the data.*

[HCP4]

The topic of delivery also extended to the possibility of rebuilding the application inside existing systems that are established and approved (F5.5). This comes with several complexities, as many NHS systems will have their own independent and legacy systems, which would require rebuilding multiple apps for multiple systems to multiple standards and requirements. It was also noted that, as the prototype is web-based, most existing NHS systems would be able to access it with ease, and keeping it as a separate standalone app would benefit it more than direct integration. An HCP-specific feature that arose regarding how HCPs would access the data collected and how it can be used to benefit their existing processes. One feature would be the ability to break down and filter options for how data is displayed to all users (F5.6). They also raised consideration into the interoperability of the data and how it can be shared and stored in established systems (F5.7), with concerns regarding data governance and how different layouts and structures in existing systems may make integration very difficult.

*How will healthcare staff access this information? In particular, if you're accessing an application or whatever in terms of not only the confidentiality, but how that will mesh, and if it will play nicely with the NHS systems?*

[HCP6]

*I guess the difficulties that you have is across the country. Is that different people have different electronic records and some of them have got more advanced patient portals than others and…not even so much the technical bits, it's the information government side about the data sharing with NHS systems and who gets to keep it where, yeah?*

[HCP1]

### Alzheimer’s Scotland Evaluation

Analysis from people living with dementia and IC in the Alzheimer’s Scotland workshop (n=8) showed a significant departure in priorities and topics when compared to the HCP in the NHS workshops. While the NHS evaluation focused significantly on the behavior dimension, participants of Alzheimer’s Scotland were far more concerned with the word dimension, particularly regarding the choice of questions that were used.

Participants’ main priority was regarding the reliability and validity of the questions and answers, and they were keen to get the new questions validated (F6.1). While participants did have concerns with the validity of the updated questions, there was very little call for restoring the original QoL-AD PROM questionnaire (F6.7).

*How do you know that? When somebody says they're not, their “Fair,” their “fair” is somebody else’s “good”?*

[IC3]

*Sorry, so just thinking it's very subjective and if you look at what you feel like today. And yes, it could change that, you know, and so how reliable is it?*

[PWD7]

*I just think there's so much. Relating to reliability and validity. Measuring what you say you are and then validity in the actual meaning of everything of each word, and so on. I have a lot of concerns, with that.*

[IC3]

The next priority regarded the usability of the interface regarding layout, font sizes, and use of colors, as well as some of the terminology used (F6.2). Some participants found the use of colors distracting, though they were pleased at the option to turn colors off. Participants also found the text and line graphs to be far too small and thin to see and would prefer to see their size increased.

*Yeah, for me I would like to see just plain black and white really.*

[PWD8]

*I think the colours are actually distracting, yeah.*

[PWD7]

*Hey DK how would that work with the graph that comes at the end that shows you know the variation in health and mood? In that the colours are helpful.*

[IC2]

*Yes, yeah, but would you be able to read the words below the drawings. You know you've got this line graph like Oh yeah, yeah. And were supposed to be able to read them. They're tiny?*

[PWD7]

The next priority for the Alzheimer’s Scotland group was ensuring HCPs and general practitioners (GPs) were on board with using the system (F6.3). Participants vented frustration that the level of interaction with their GPs was minimal. Therefore, it is paramount that future researchers ensure cooperation and involvement from GPs and HCPs to use the system before implementing, as the data would be useless if they do not use it.

*Yeah, and also how practical is it? I've not seen a doctor for quite a while. I actually had a phone call with her last week and there is just not the time to do something like that.*

[PWD8]

*I was thinking also, but in terms of GP, GPs aren't even doing an annual review. So, the prospect of him trying to get only a GP and take this along and look at it. Yeah, you know it's. I don't think you know. 1 of the missions are things we have to change the attitude of GPs as well to looking after people even say once a year. yeah.*

[IC3]

*I don’t want to be pessimistic. But yeah, the time factor just wouldn't allow it.*

[PWD8]

A theme was added to the MoSCoW questionnaire due to its significance in the NHS workshop, regarding further research into mobile-friendly and application versions of the prototype (F6.4). The MoSCoW response showed a moderately high interest in the prototype to be designed for this, though the topic itself did not arise during the workshop.

As with earlier phases, participants disliked the use of a voice modality (F6.6), with participants finding it overly robotic and 1 participant describing the voice having “disturbed” them and felt the voice sounded “manipulative.” While participants did recognize the usefulness of having a voice option for people who may struggle to read, each participant would choose to disable the voice feature if given the option.

*Are you going to use that voice? Why is it an American accent?*

[PWD7]

*I just feel. Disturbed by being manipulated about my deepest things by Alexa [Digital Voice assistant].*

[PWD3]

*Is it and it just feels like totally manipulative of everything. Hmm, yeah. Yeah, definitely don't want you, don't want you expect when you're dealing with intimate things where you when you look at the sensitivity.*

[PWD3]

*You know they would be nice to have that option. Especially because then PWD8 speaks about, you know, just difficulty they you know seeing you know the words and reading due to Alzheimer’s that if you had the voice activation option. Yeah, it could read the question, but I do agree. I think in that robot version. Yeah, it is. Yeah, it's not so clear and things as well, but I think there's more options that they have to have that.*

[PWD10]

Finally, on the topic of encouraging user participation (F6.5), people living with dementia who attended the workshop without an IC stated they would have no issues completing the prototype themselves regularly, though 80% of participants who completed the demographic questionnaire described themselves as being “somewhat or very confident” using technology. People living with dementia who attended with an IC stated they would likely need help from their IC to complete.

*I think I would be comfortable doing it myself.*

[PWD2]

*Oh, I’d do it myself. I think it mustn’t be very difficult, different for Everybody.*

[PWD8]

***IC2:*** *Would you need my support to fill in the questionnaire? To this. If this was sent to you. Would you feel happy just to do it or would you like me to be there to help you?*

***PWD3:*** *I would, I would need support, yeah.*

***IC3:** Well, my husband would certainly need my support.*

## Discussion

### Principal Findings

The purpose of this study was to co-design a novel digital application to enable people living with dementia to regularly self-report QoL PROMs. This aim was successfully achieved as demonstrated with a co-designed FFP that adheres to modern web standards [35,36] and DEEP (Dementia Engagement and Empowerment Project) guidelines [37-39]. The prototype was co-designed with direct involvement from people living with dementia during every stage of development and was evaluated by key stakeholders (including people living with dementia, ICs, and HCPs) around features, questions, and modalities alongside design considerations, which are discussed below.

### What Features, Questions, and Modalities Do People Living With Dementia Prefer When Interacting With a Digital Application That Enables Them to Self-Report QoL PROMs?

Existing PROM terminology was deemed inappropriate and noninclusive, with the format of certain questions being overtly long, ambiguous, irrelevant, and confusing. Participants requested frequent changes to the questions over the course of the prototype development. Regarding modalities, participants preferred text-based modalities, describing visuals, icons, and animations as distracting, with the use of colors being divisive among participants. Voice modalities were universally disliked and seen as intrusive, aggravating, and “just bad.”

### Use of Dementia PROM Questions

Existing PROM terminology was deemed inappropriate, with participants reporting these questionnaires to be noninclusive, overtly long, ambiguous, irrelevant, and confusing. While QoL-AD is a well-established and validated tool designed specifically for people living with dementia [40], it has seen limited clinical use with many studies opting for more generic PROMs [17,18]. These studies did not report a reason for using generic PROMS over those designed for people living with dementia, though these studies also lacked involvement from people living with dementia in a co-design capacity. This study’s findings show a need to review existing PROMs developed for people living with dementia. Many of these PROMS were originally developed as only paper-based questionnaires to be used in a clinical setting and delivered by an HCP [17], with very few high-quality QoL PROMs validated for use in a home environment [20]. With the rise in ePROMs [41] and the convenience of online questionnaires [42] with comparable response rates compared to traditional methods [7], it becomes clear that more needs to be done to update dementia PROMs for digital use.

### Preferred Modalities

Participants showed a significant preference for text-based modalities, with visual modalities often resulting in indifference or conflict, and voice modalities being significantly disliked. This is to be expected, as since their inception, PROMs have been primarily text-based [12], with the concept of multimedia PROMs and protocols for it still being relatively new [43]. While there are several paper-based PROMs that use visual elements [44,45], the use of images and icons was deemed patronizing and unprofessional by people living with dementia participants during cycle 1. Such patronizing actions can have a dehumanizing effect on people living with dementia that reinforces negative stereotypes and should be avoided [46]. Participants, therefore, preferred visual flourishes that focus on larger and bolder text fonts (F1.1) and the use of visuals to highlight interactive elements (F1.3). These statements are backed up by other literature that notes that culture and language play a key role in the use of PROMs, and issues with misinterpretation can impact their reliability and validity [47]. Finally, while voice modalities were universally disliked, they have been shown to work as an effective interaction method for people living with dementia, with voice assistant technology being recognized as being mature enough to support health care delivery [48]. The type and style of voice used should be explored to appeal to participants keen to use a voice modality [49], as well as concerns about understanding accents as a major recognized weakness of speech recognition technology [50]. More research is therefore needed for voice modalities to be effective.

### What Are the Design Considerations and Specifications for a Digital Application to Enable People Living With Dementia to Self-Report QoL Data via PROMs?

Overall, participants enjoyed the layout and design of the prototype, finding it highly accessible, and envisioned using it at home without needing assistance. Participants lauded the importance of personalization and optionality. Rather than having fixed features, participants requested the ability to turn features such as color or modality on or off, as well as the ability to skip questions. Participants were also keen on being in control of their own data, requesting access rights to their results as well as the ability to choose with whom and when to share the data.

### Personalization and Optionality

A key feature that arose multiple times during the workshops was the importance of personalization and optionality of the prototype. This matches the background research, which notes the importance of individuality for people living with dementia and how technology interventions need to be adaptable and adjusted to specific users [18] and how engaging early with people living with dementia in the design process to make such adjustments can benefit a variety of different technologies during the development process [51]. Current policies for the future of digital health technologies highlight the importance of personalized approaches tailored to patients’ needs [52], with HCPs increasingly encouraged to implement more personalized approaches in all facets of medicine and treatment [53], including dementia care [54]. Lack of personalization and adaptation is already a recognized issue with existing technology “designed for people living with dementia” [55,56], which is compounded by the lack of involvement and inclusion of people living with dementia in developing technologies which are meant to serve them [57]. Technology that is not designed with personalization in mind may struggle to be adopted as users in all domains will have vastly different needs, perceptions, and capabilities, meaning that implementing any modern technology solution requires consideration of this user diversity [58]. A “one size fits all” solution would not work, and with an already high and increasingly aging population impacted by dementia, there is a risk of the “digital divide” expanding even further, with increasing social inequalities in this domain being driven by technology that excludes people living with dementia and other vulnerable members of society by being inaccessible or not adaptable to their needs [26,59]. This, of course, does increase the level of complexity of an application and the work required to develop, test, and support it. Having too many features and options can result in feature fatigue for users, which can overwhelm them and make an application more difficult to use [60]. Therefore, a careful balance must be struck between optionality and complexity.

### Data Ownership and Autonomy

Another feature was the importance of data ownership and autonomy. Patient autonomy has long been a vital aspect of health care, with HCP respecting patients’ opinions and offering them the opportunity to make informed decisions about their medical treatment [61]. People living with dementia are increasingly being encouraged to be involved in such decision-making [62] with the goal of using technologies to provide medical treatments remotely that will enable patients to stay at home for longer [52,63] well into the later stages of dementia [54]. The danger here is that such technology in a home environment can be intrusive, collecting sensitive and personal data on patients who may not be willing to give or be aware of such a thing, which could be risks for privacy violations or data breaches [64,65]. It is therefore critical that patients have the autonomy to access and control their data and how it is used. Privacy concerns remain one of the top barriers to technology adoption among the over fifties [66], with polls showing almost half (49%) of older adults raising privacy concerns with telemedicine delivery [67]. Regardless, an extended effort should be made to enable and encourage data autonomy among patients. This approach has seen significant success in Estonia, where the Estonian eHealth Patient Portal enables patients to access all health care data regarding them on request and allows them to deny access to their data to any or all care providers [68]. This enables an unprecedented level of patient engagement and trust, with Estonia recognized as having one of the leading eHealth solutions in Europe [69].

### Conclusions

While this study did successfully develop and evaluate a multimodal digital application to enable people living with dementia to regularly self-report QoL PROMs, this work opens further discussion about the wider changes that are required to facilitate regular use of PROMs in routine postdiagnostic dementia care. More research is needed to update preexisting dementia PROMs or validate new dementia PROMs that are designed with digital delivery in mind; solutions should prioritize text-based modalities and avoid voice-only modalities; any technology solution developed for people living with dementia should be designed with personalization and optionality in mind; and data autonomy for people living with dementia should be considered as a top priority, for applications developed to collect QoL data, with people living with dementia given access and control of how this data is shared and used.

### Strengths and Limitations of This Study

The main strength of this study was that the novel application was co-designed with people living with dementia from the outset, with people living with dementia involved directly throughout every stage of development. By including people living with dementia in the entire development of the prototype, we were able to include and remove features and create a prototype that was far more likely to be acceptable and adopted by people living with dementia and serve their needs. We also believe this is one of the first studies to evaluate perceptions from all key stakeholders, including people living with dementia, ICs, and HCPs, regarding the use of ePROMs for dementia in routine care.

The main limitation of our study came as a result of needing to perform entirely online. This caused significant issues with recruitment. Multiple potential participants excluded themselves from this study due to it being online. Many people living with dementia and IC reported not feeling confident enough to take part in an online study, or felt that spending any extended length of time on their computer was exhausting. HCP also reported that many possible participants were overfatigued by online meetings during the pandemic and would not wish to participate in further online meetings. This resulted in a small and homogeneous participant group that likely does not represent the population at large. This itself was an additional limitation as our co-design group and most of our evaluation group were White and native English speakers and did not adequately represent participants from different cultural and language backgrounds. A further limitation was that, as people living with dementia were the central focus of co-designing the prototypes, the voice of IC may not have been heard. While ICs were involved in every stage of development, it may be fruitful in the future to investigate the specific perceptions of ICs. Finally, the inclusion criteria did not permit people living with dementia in later stages of the condition to take part. This was a pragmatic choice as this would have added significant additional complexity to this study, especially regarding the ethical approval required for participants who cannot provide informed consent, and the additional resources needed to ensure their safekeeping and well-being. This was therefore outside the scope of this study and is something to investigate further in the future.

### Future Research

With a functional prototype, the next stage for this project would be a pilot in a real-world environment. Future participants with early stages of dementia would be encouraged to self-report the QoL questionnaire using the novel application remotely using their own device in their home environment, while HCPs access the data and evaluate its clinical utility. Future research would also strive to revisit and test the prototypes on participants with later stages of dementia to gain insight into understanding the app’s usability at stages where problematic behavior is more prevalent, to see if the prototype fits their use case. If this proves viable, new features could be developed for this particular population. We would also aim to recruit participants from a wider range of backgrounds, especially those from ethnic backgrounds or where English is not their first language. We would also seek to investigate further opinions from IC and recruit an expanded pool of them, such as extended family and friends who may offer greater insight and observation from a home environment, especially when piloting the novel application remotely. Finally, more research is needed into ePROMs that are designed to enable people living with dementia to self-report QoL data. The novel application is a good proof of concept for people living with dementia self-reporting QoL data using digital technology, so the next step would be to see if we could validate our questionnaire or create a new ePROM designed to enable people living with dementia to self-report using new technologies.

## Supplementary material

10.2196/87565 Multimedia Appendix 1 Overview of prototypes.

10.2196/87565 Multimedia Appendix 2 Video demo of final functional prototype.

## Abbreviations

DEEP: Dementia Engagement and Empowerment Project
ePROM: electronic patient-reported outcome measure
FFP: final functional prototype
GP: general practitioner
HCP: health care professional
HFP: high-fidelity prototype
IC: informal caregiver
LFP: low-fidelity prototype
MoSCoW: Must Have, Should Have, Could Have, Won't Have
NHS: National Health Service
PROM: patient-reported outcome measure
QoL: quality of life
QoL-AD: quality of life in Alzheimer disease
WFP: wireframe prototype
