# Supplementary material for: Co-Designing and Evaluating a Multimodal Digital Application to Enable People With Dementia to Self-Report Quality of Life Patient-Reported Outcome Measures: Co-Design Study and Summative Evaluation
Source: J Particip Med. 2026 Apr 1;18:e87565. doi: 10.2196/87565 (PMC13043077; doi:10.2196/87565)
Supplement: Multimedia Appendix 1 — Overview of prototypes. [file jopm-v18-e87565-s001.docx]

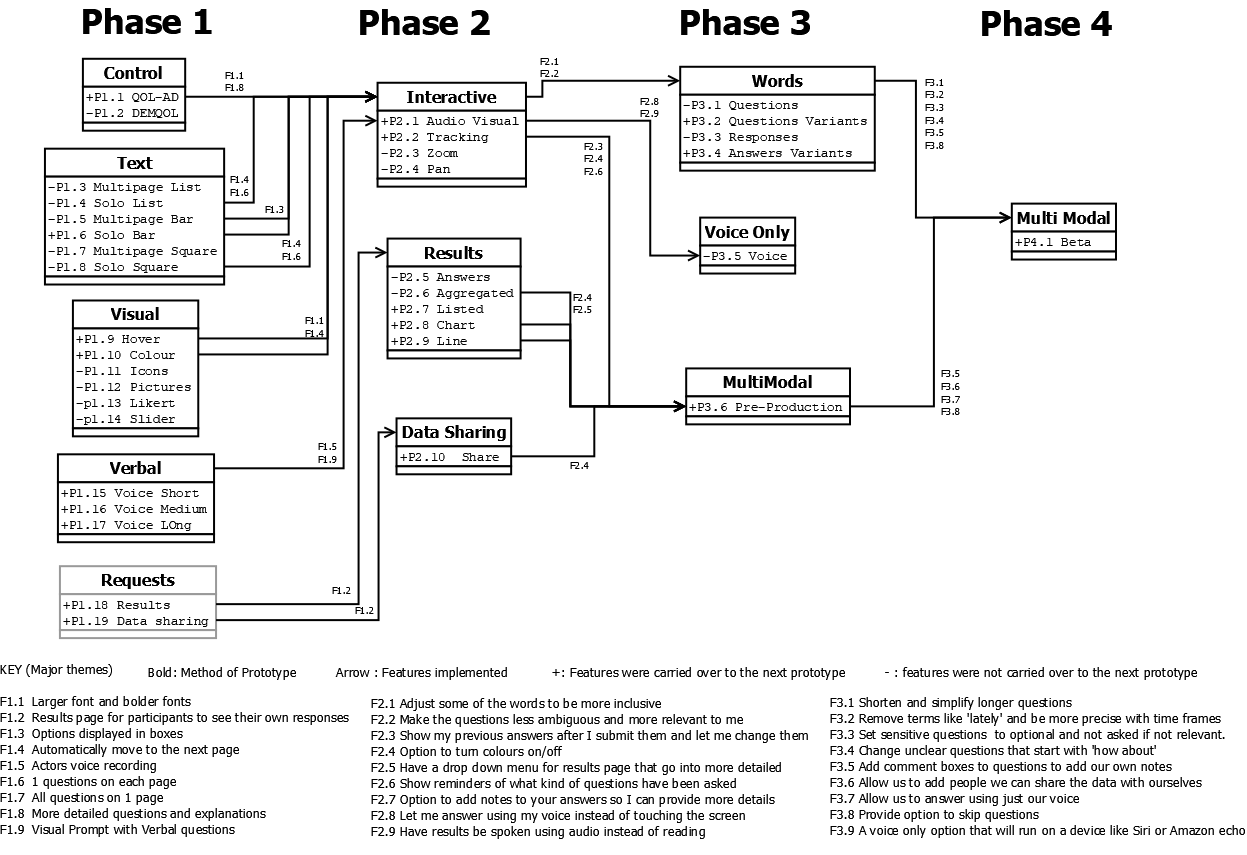


## Phase 1 Prototypes

### **Figure S1.** QOL-AD.

**
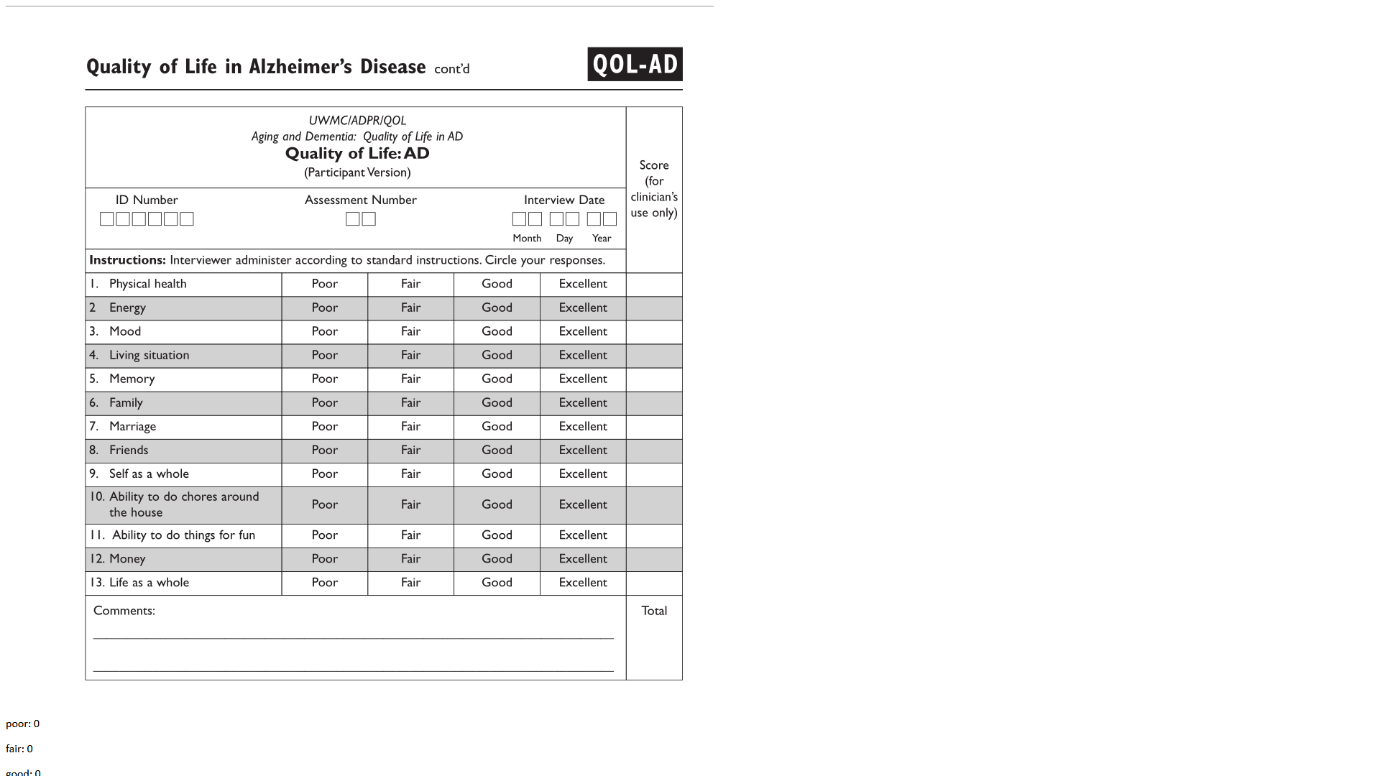
**

### **Figure S2.** DEMQOL.

**
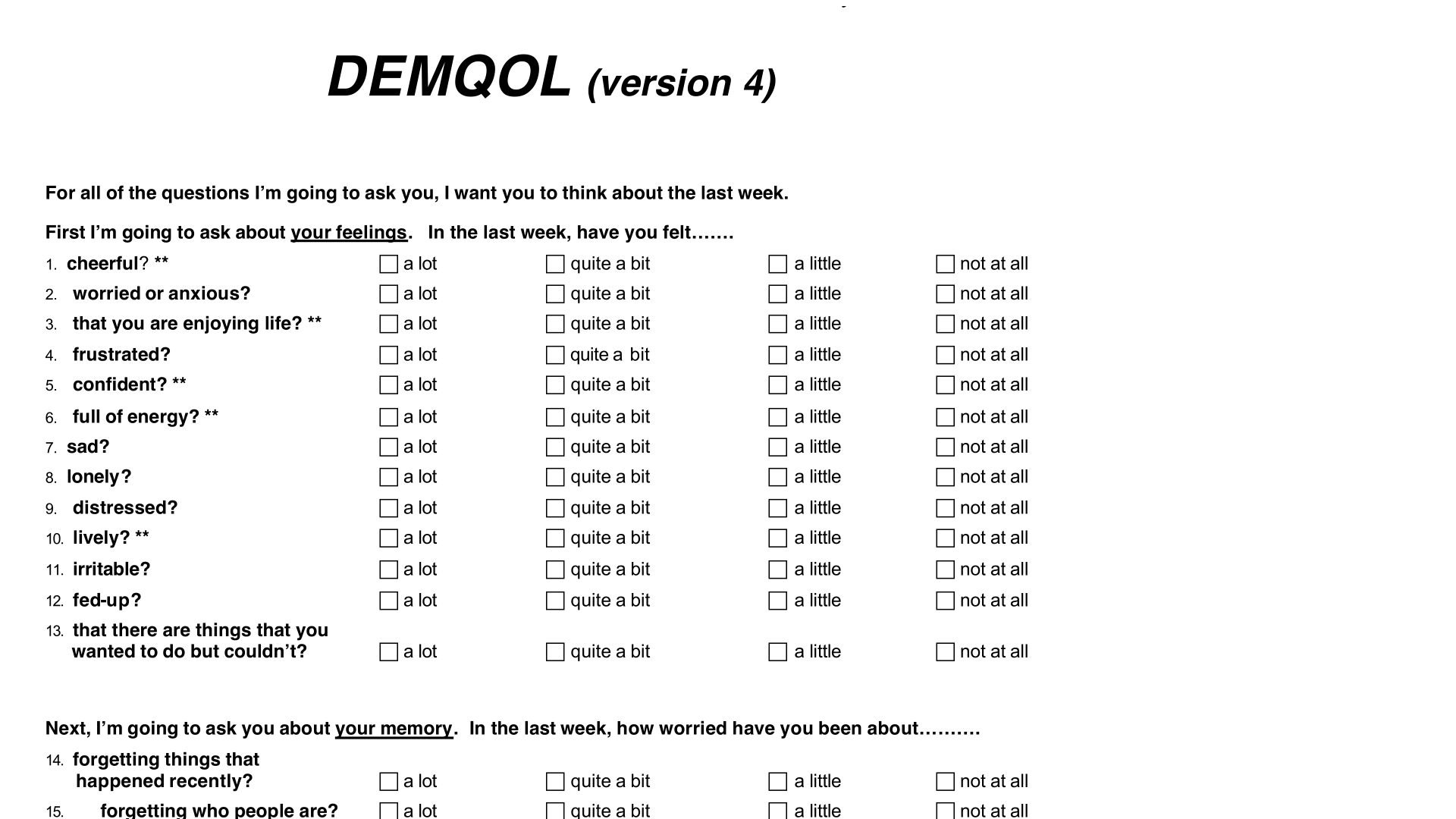
**

### **Figure S3.** Multipage list.

**
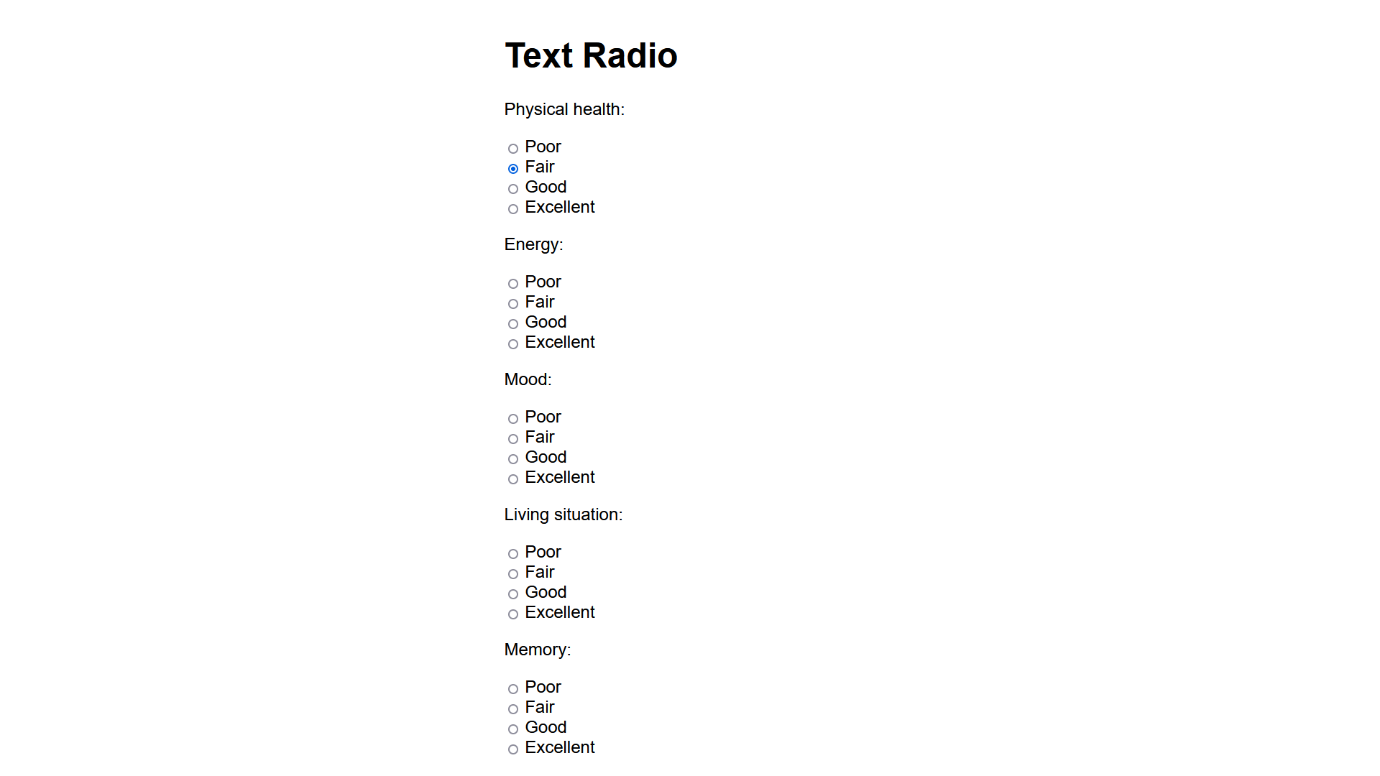
**

### **Figure S4.** Multipage list.

**
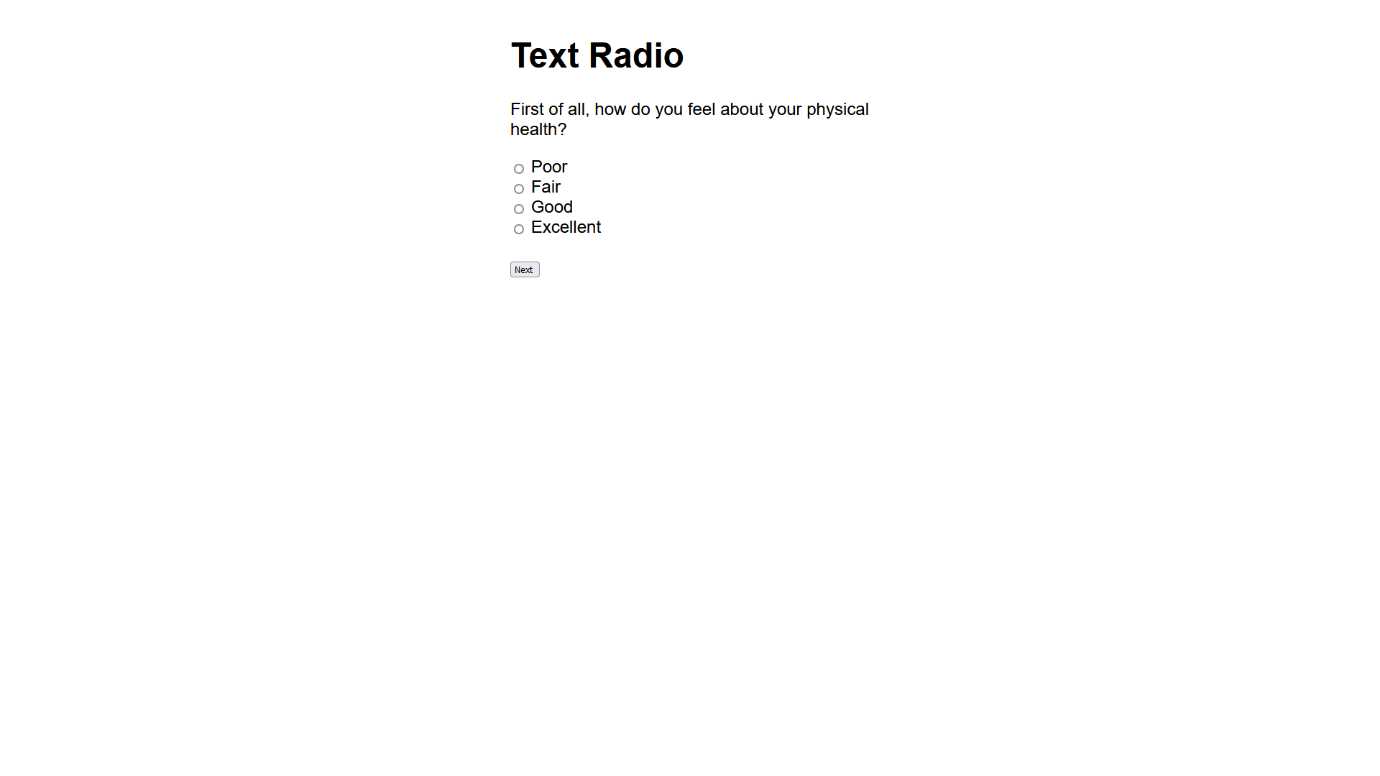
**

### **Figure S5.** Multipage bar.

**
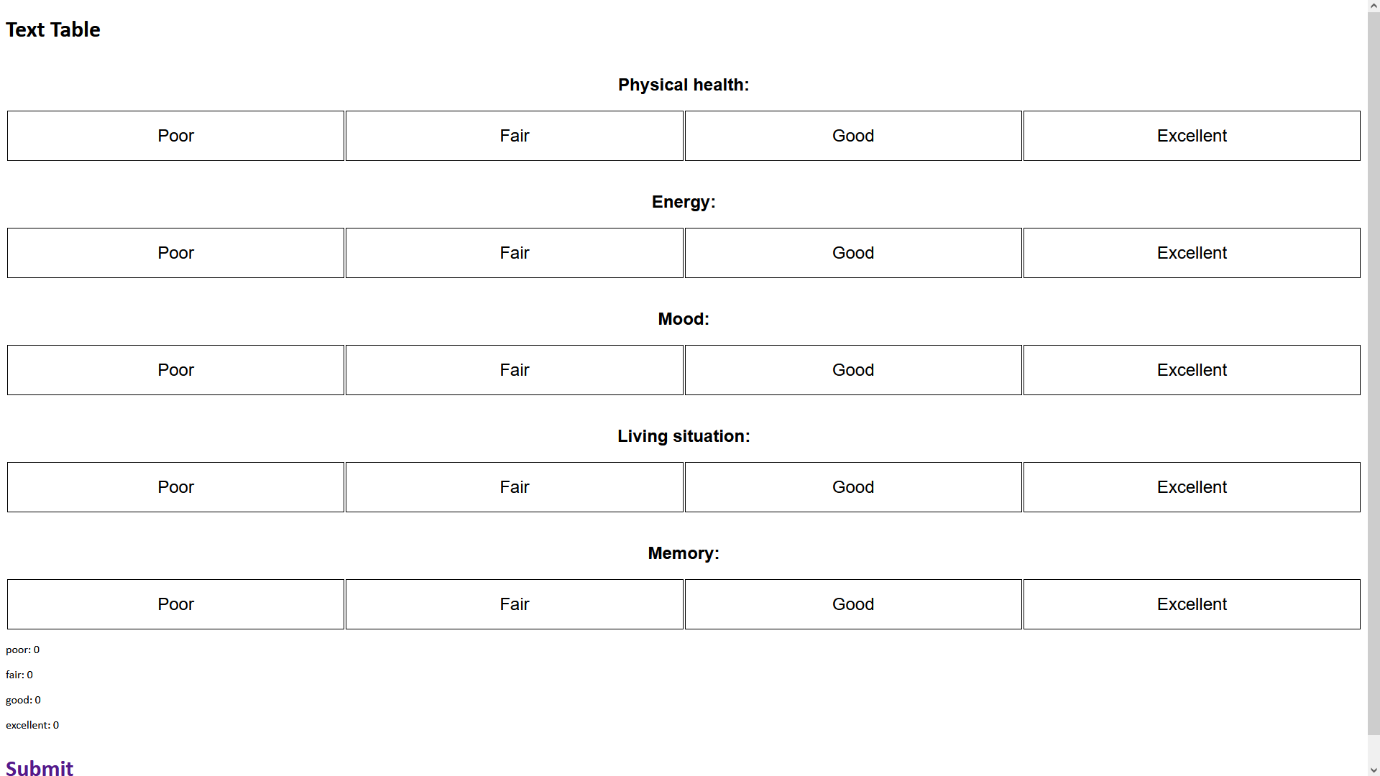
**

### **Figure S6.** Solo bar.

**
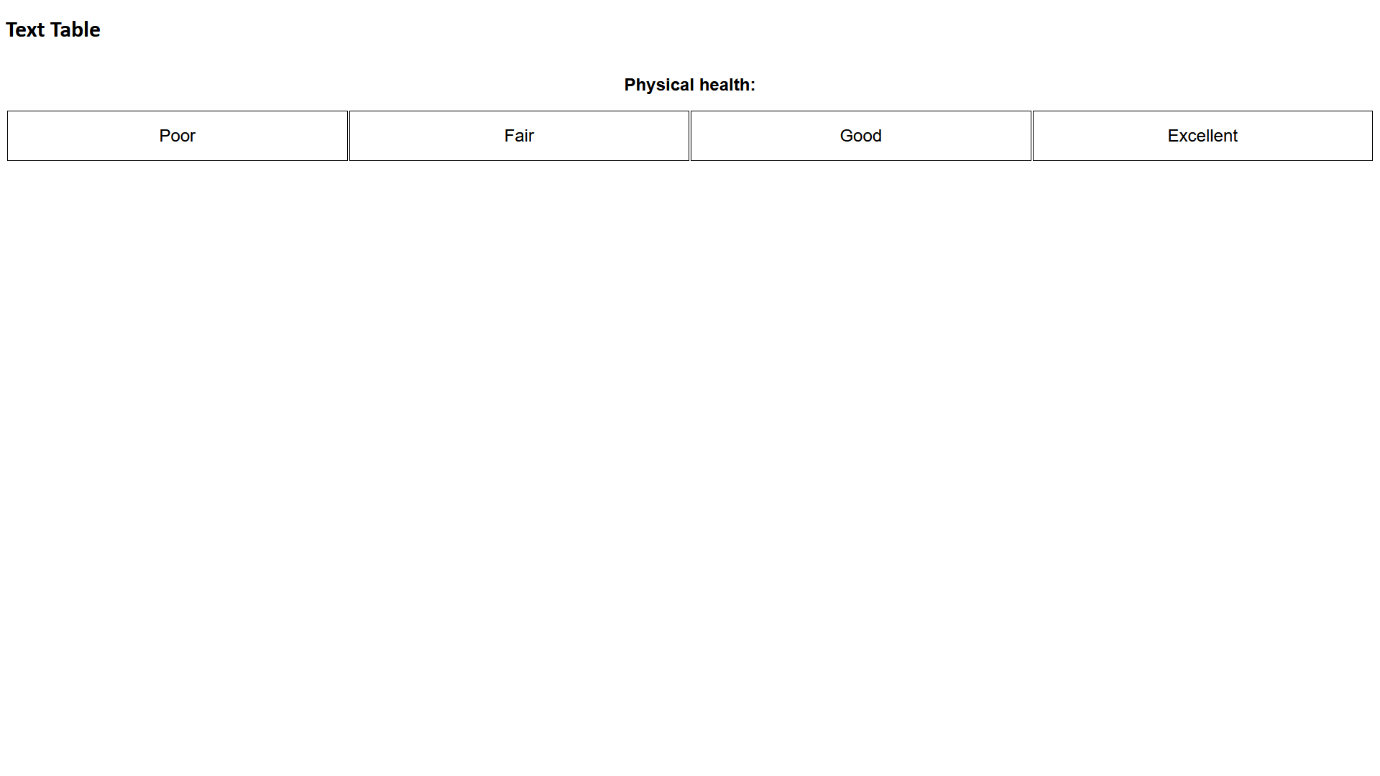
**

### **Figure S7.** Multipage square.

**
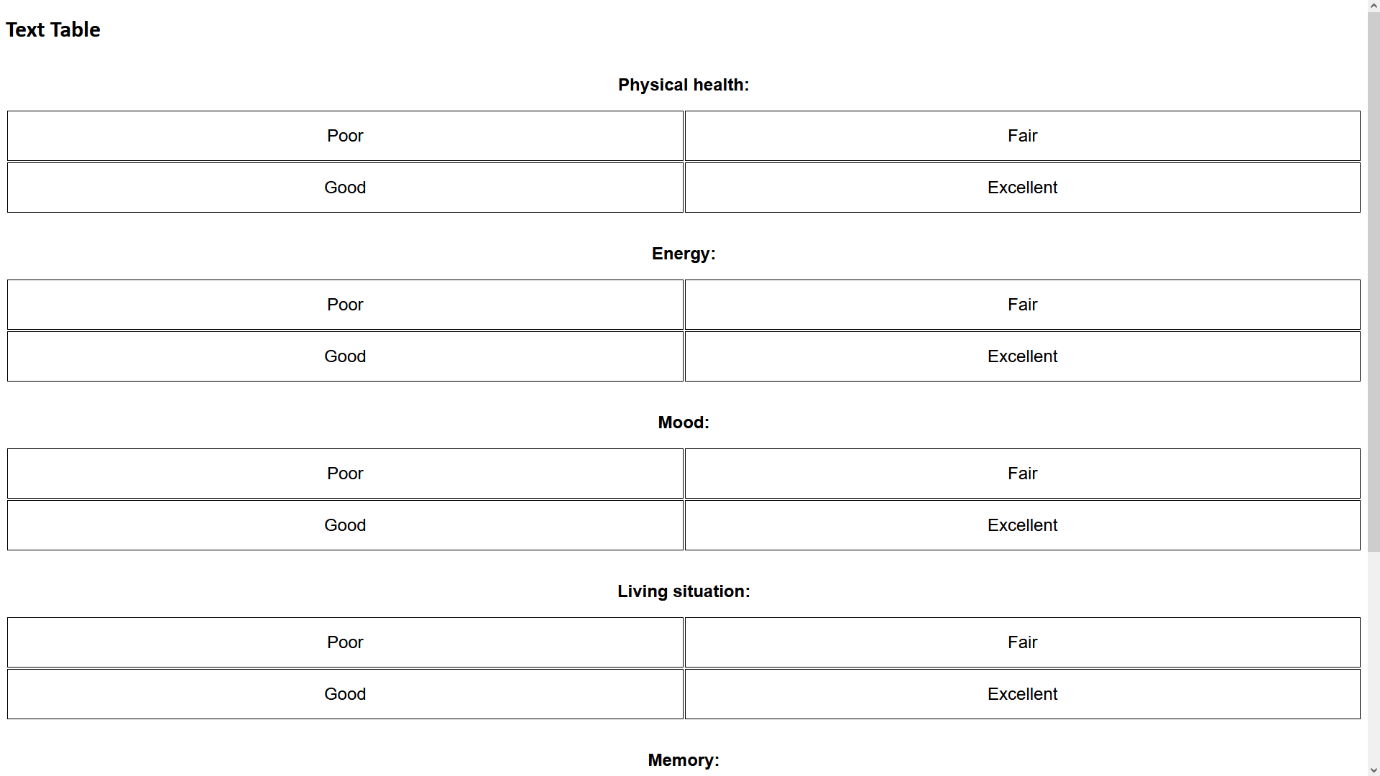
**

### **Figure S8.** Solo square.

**
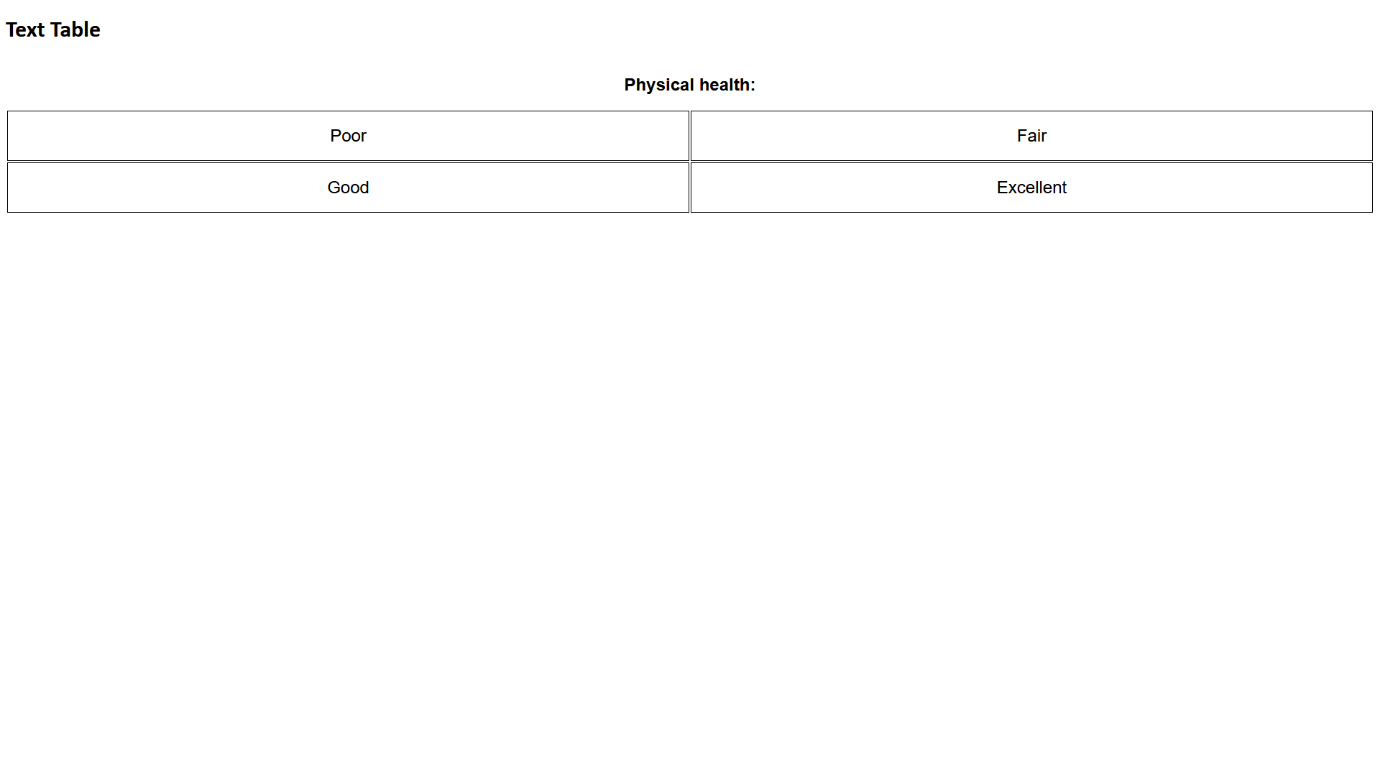
**

### **Figure S9.** Hover.

**
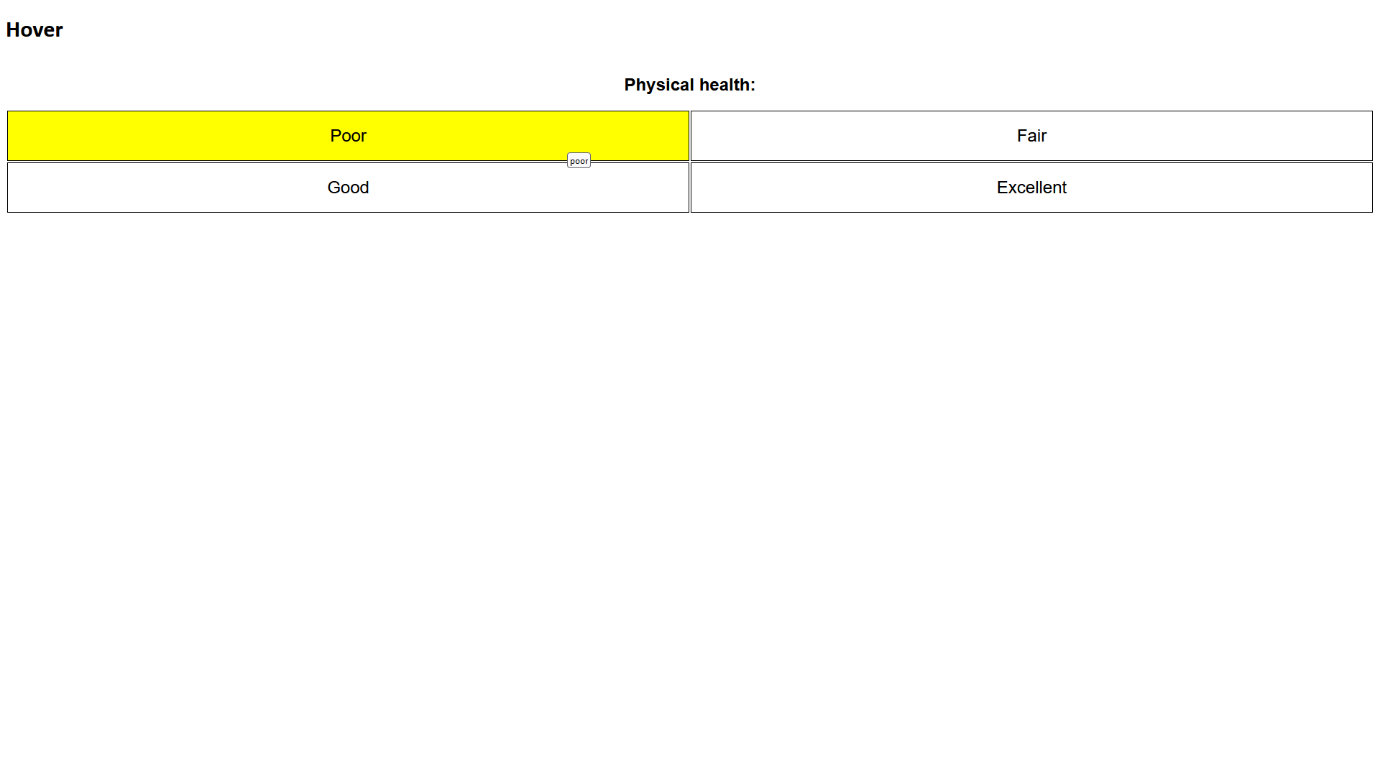
**

### **Figure S10.** Colour.

**
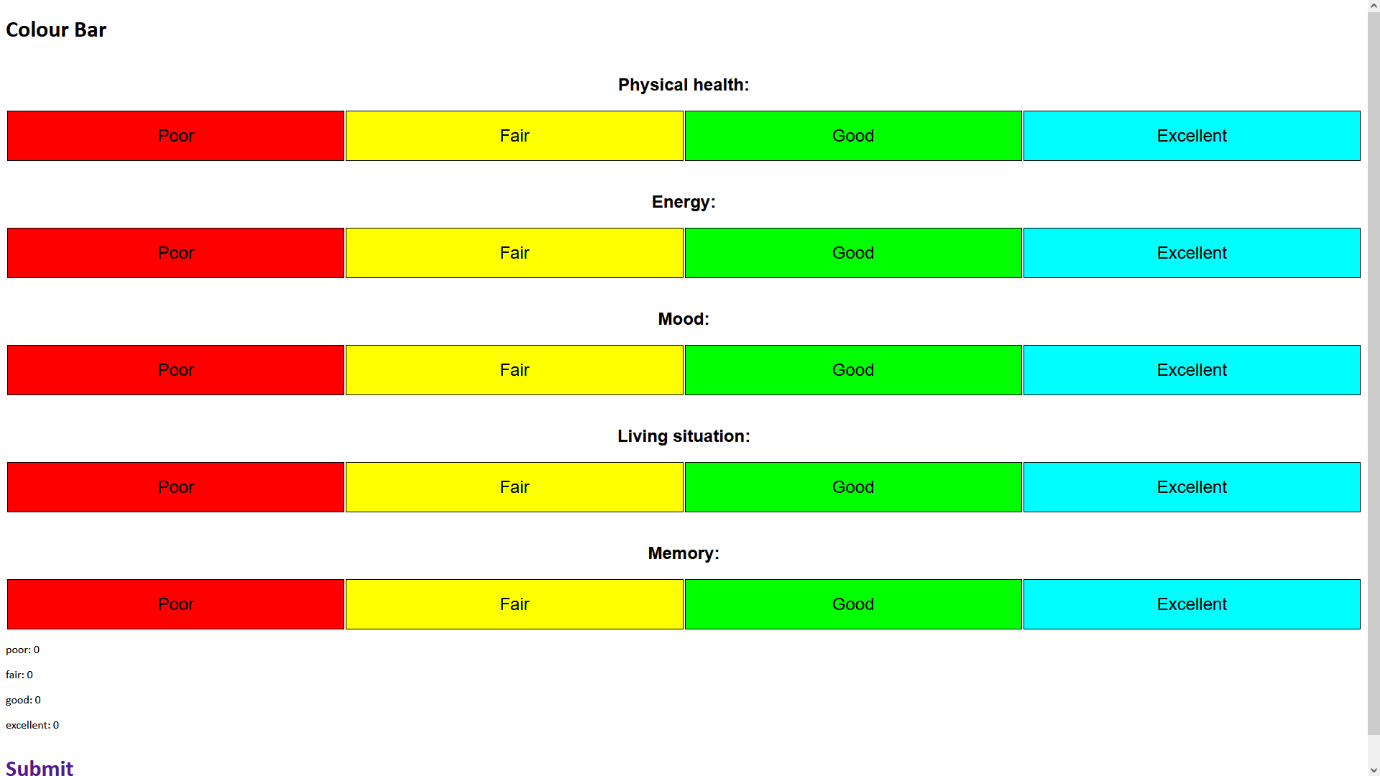
**

### **Figure S11.** Icons.

**
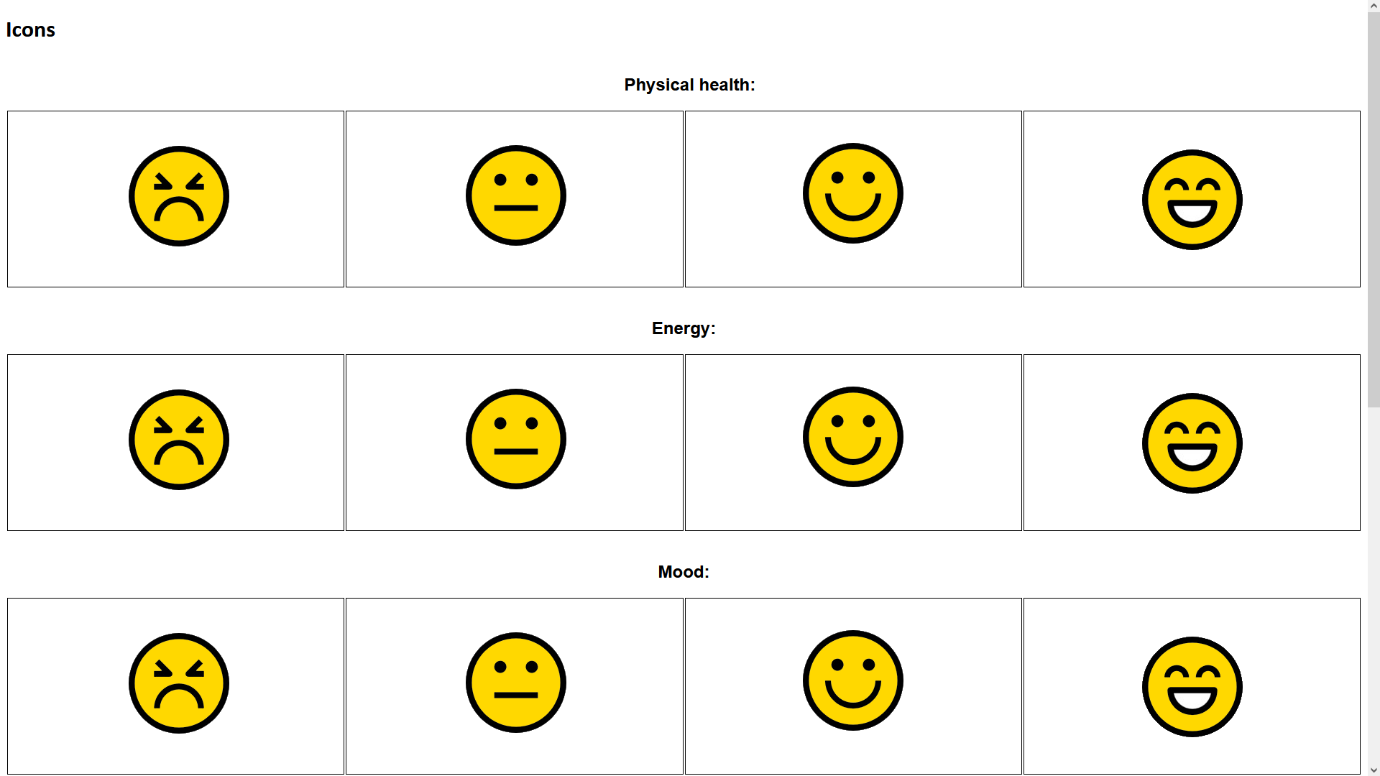
**

**Figure S12.** Picture.

**
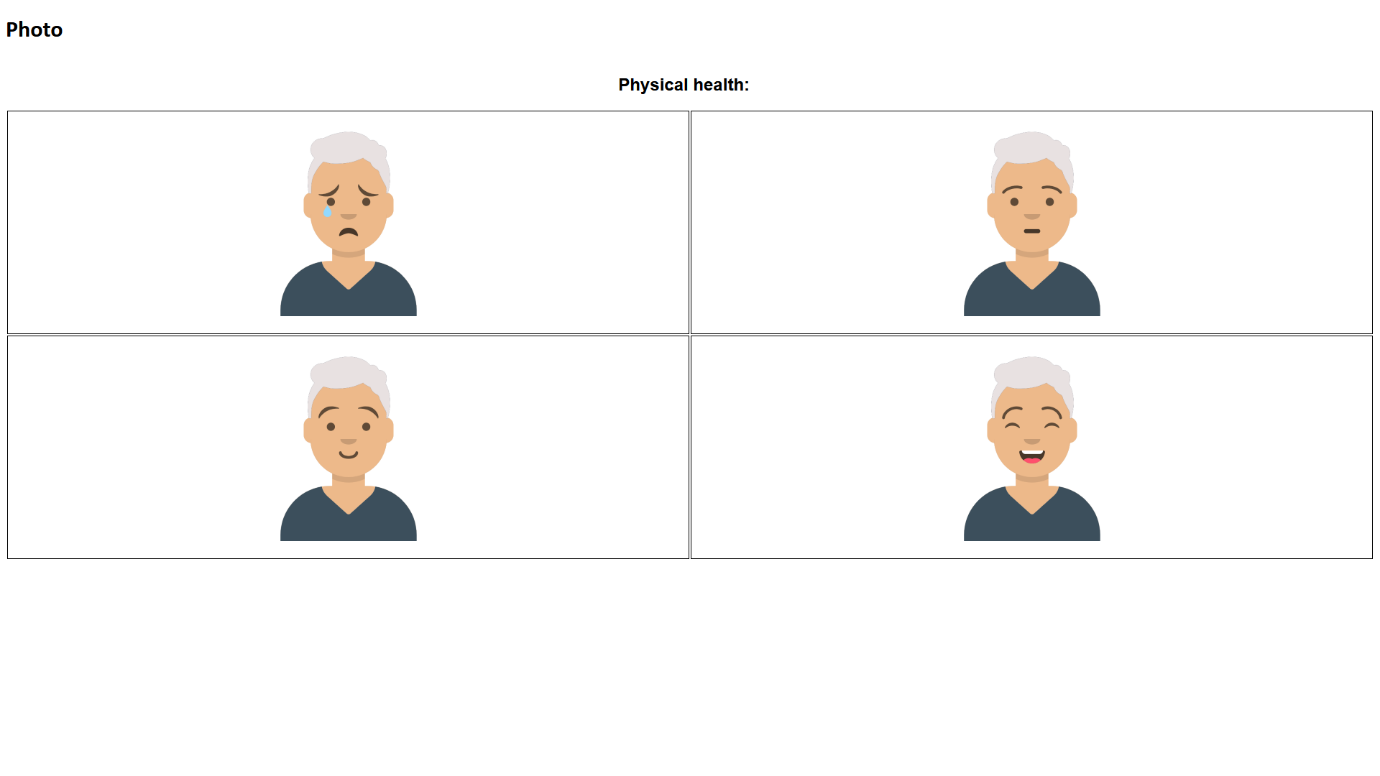
**

### **Figure S13.** Likert.

**
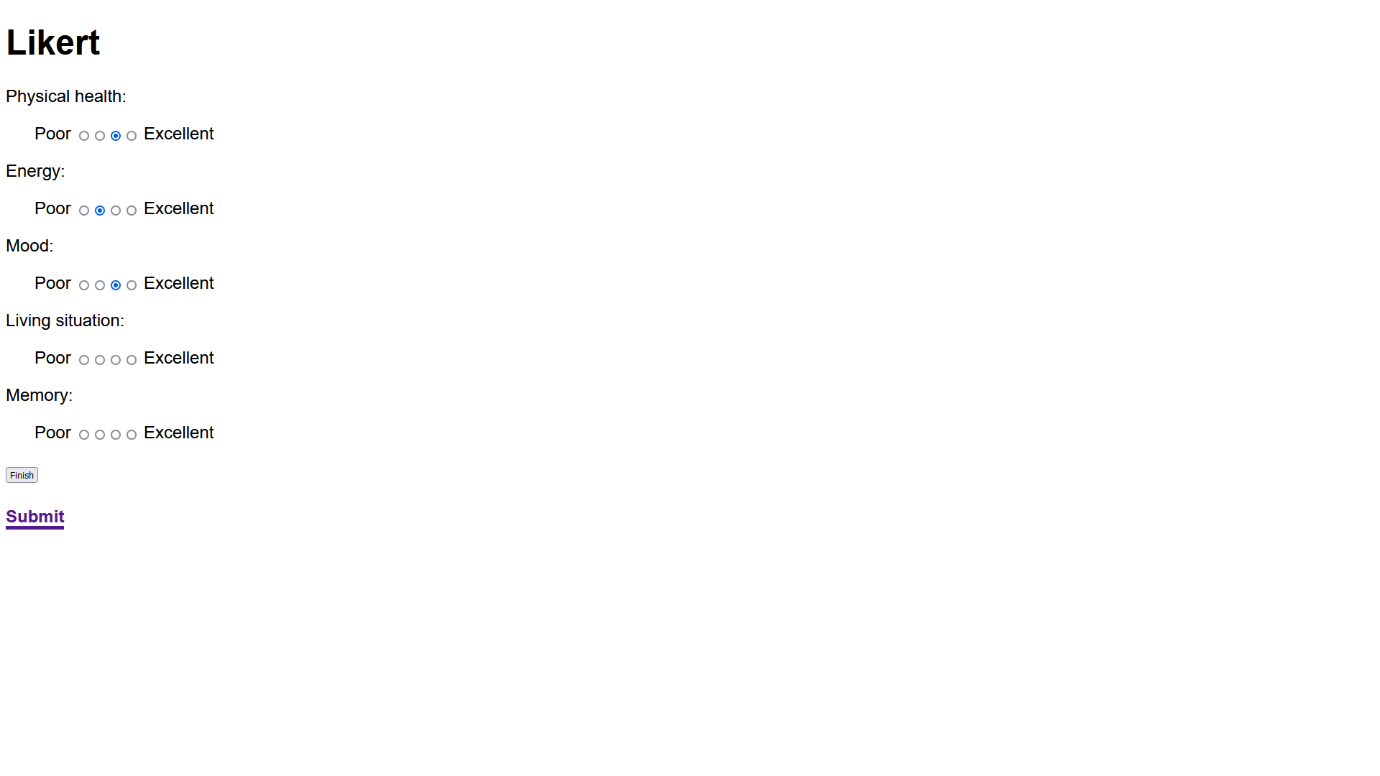
**

### **Figure S14.** Slider.

**
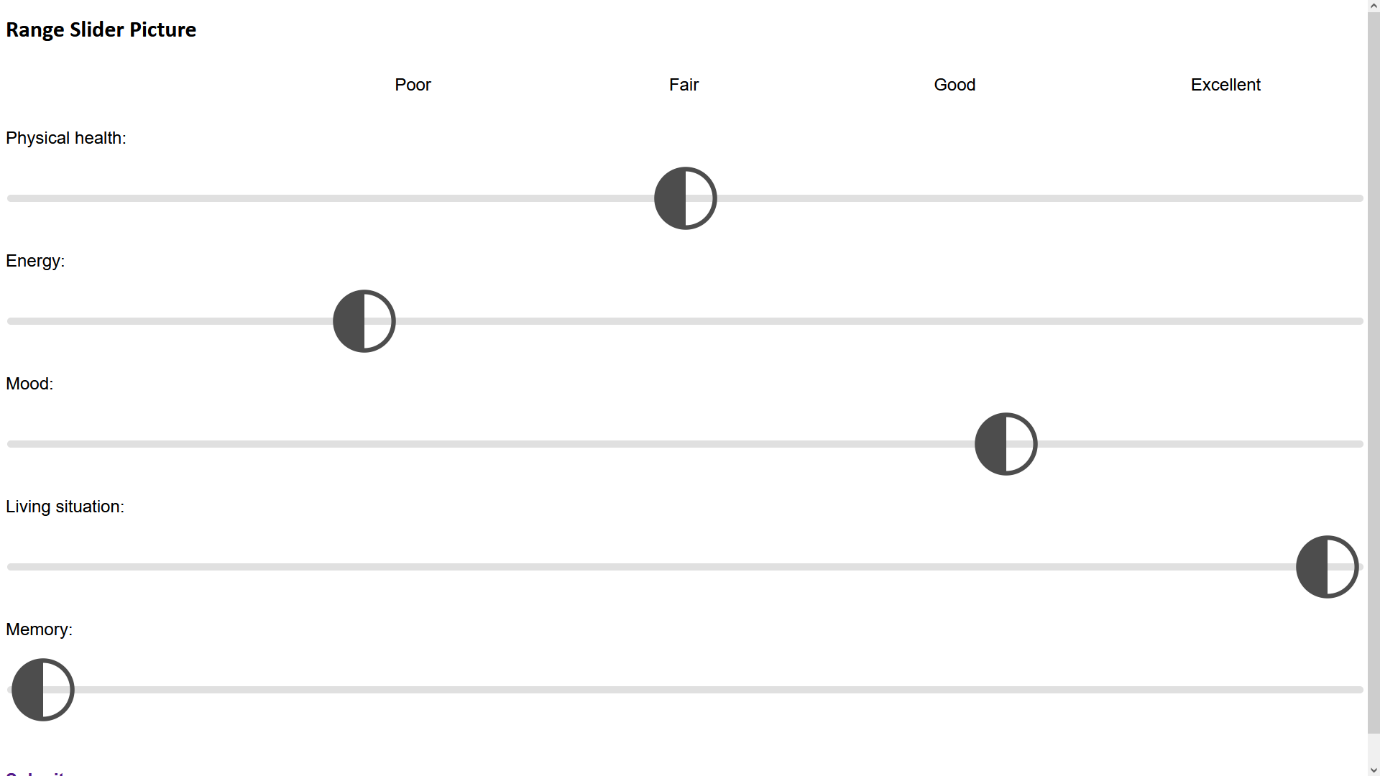
**

**Figure S15.** Voice short.

"How do you feel about your physical health?"

"How do you feel about your energy level?"

"How has your mood been lately?"

**
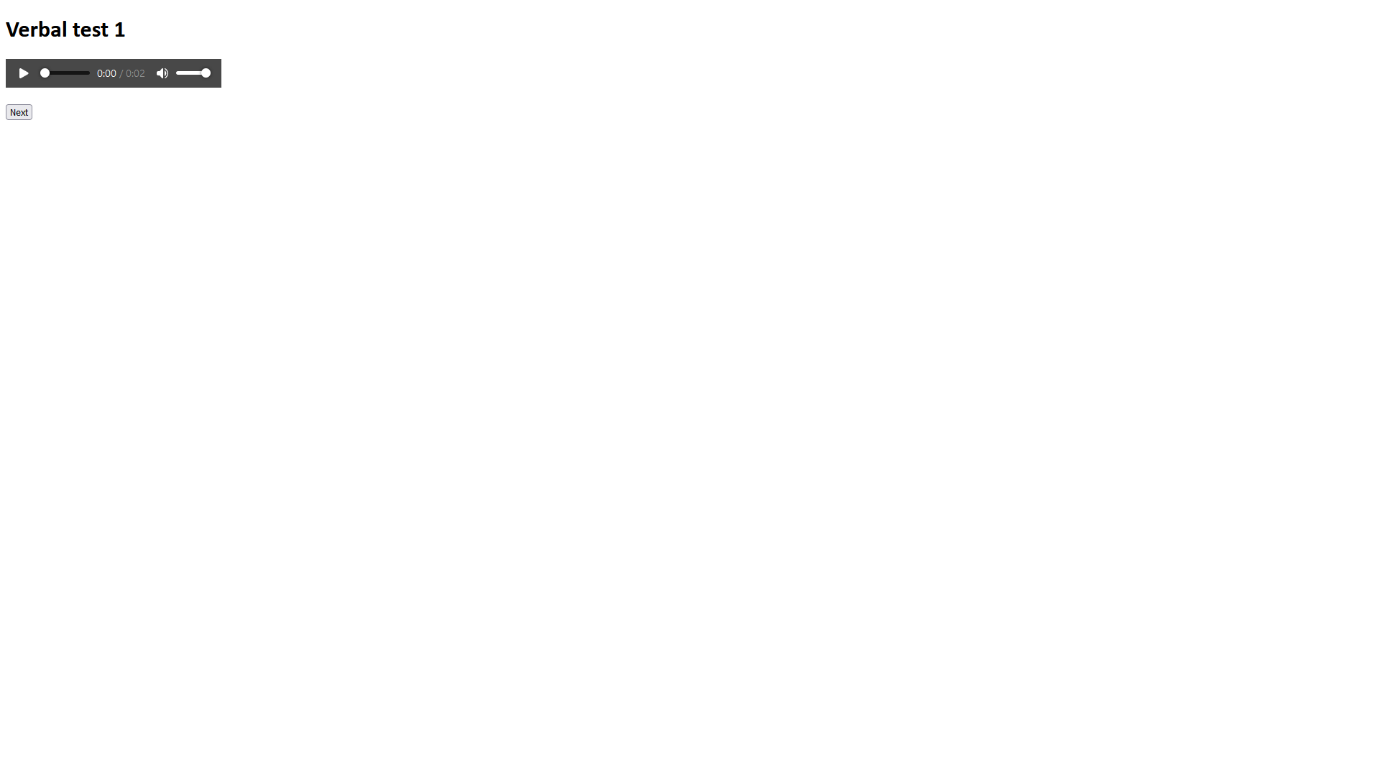
**

### **Figure S16.** Voice medium.

**
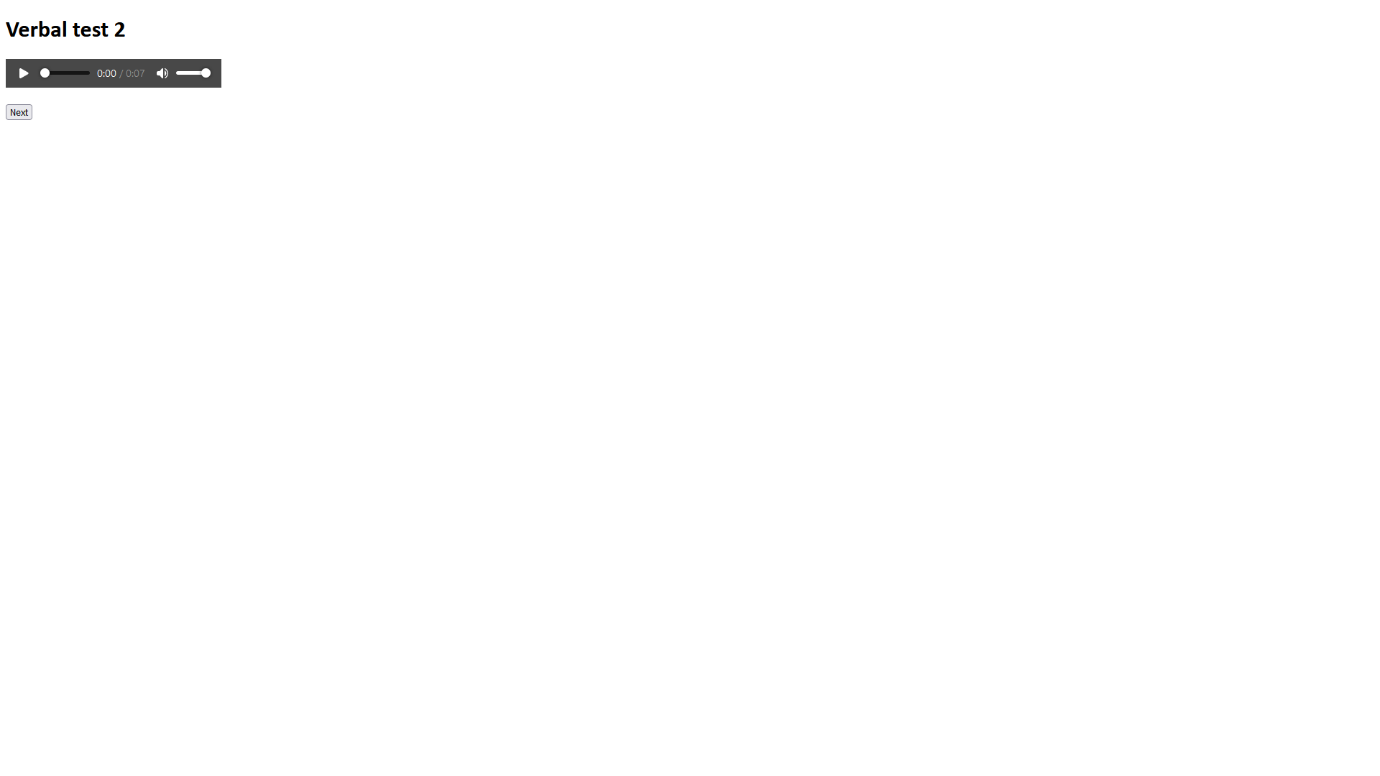
**

“How do you feel about your physical health? Would you say it’s poor, fair, good, or excellent?”

“How do you feel about your energy level? Do you think it is poor, fair, good, or excellent?”

“How has your mood been lately? Would you rate your mood as poor, fair, good, or excellent?”

### **Figure S17.** Voice long.

**
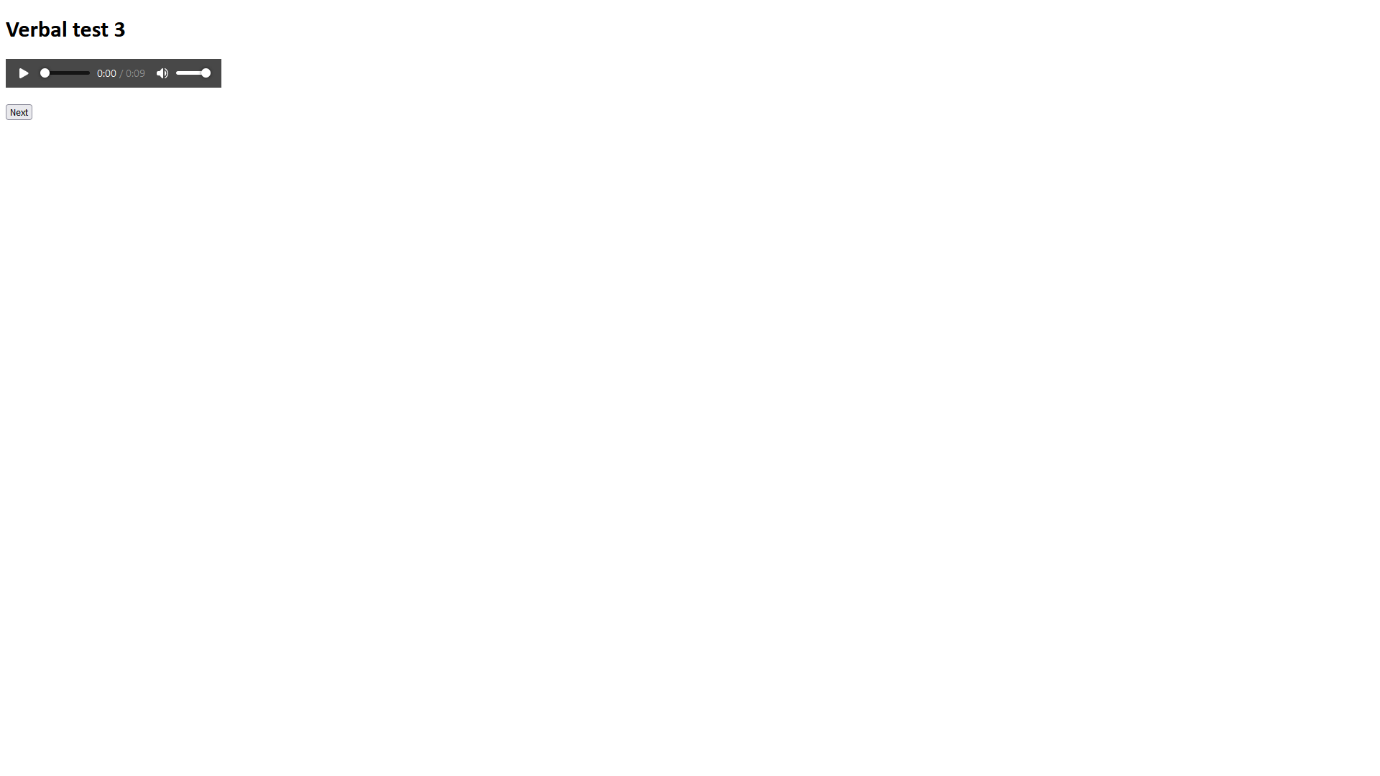
**

“First of all, how do you feel about your physical health? Would you say it’s poor, fair, good, or excellent?”

“How do you feel about your energy level? How have you been feeling most of the time lately. Do you think it is poor, fair, good, or excellent?”

“How has your mood been lately? Have your spirits been good, or have you been feeling down? Would you rate your mood as poor, fair, good, or excellent?”

## Phase 2 Prototypes

### **Figure S18.** Audio visual.

**
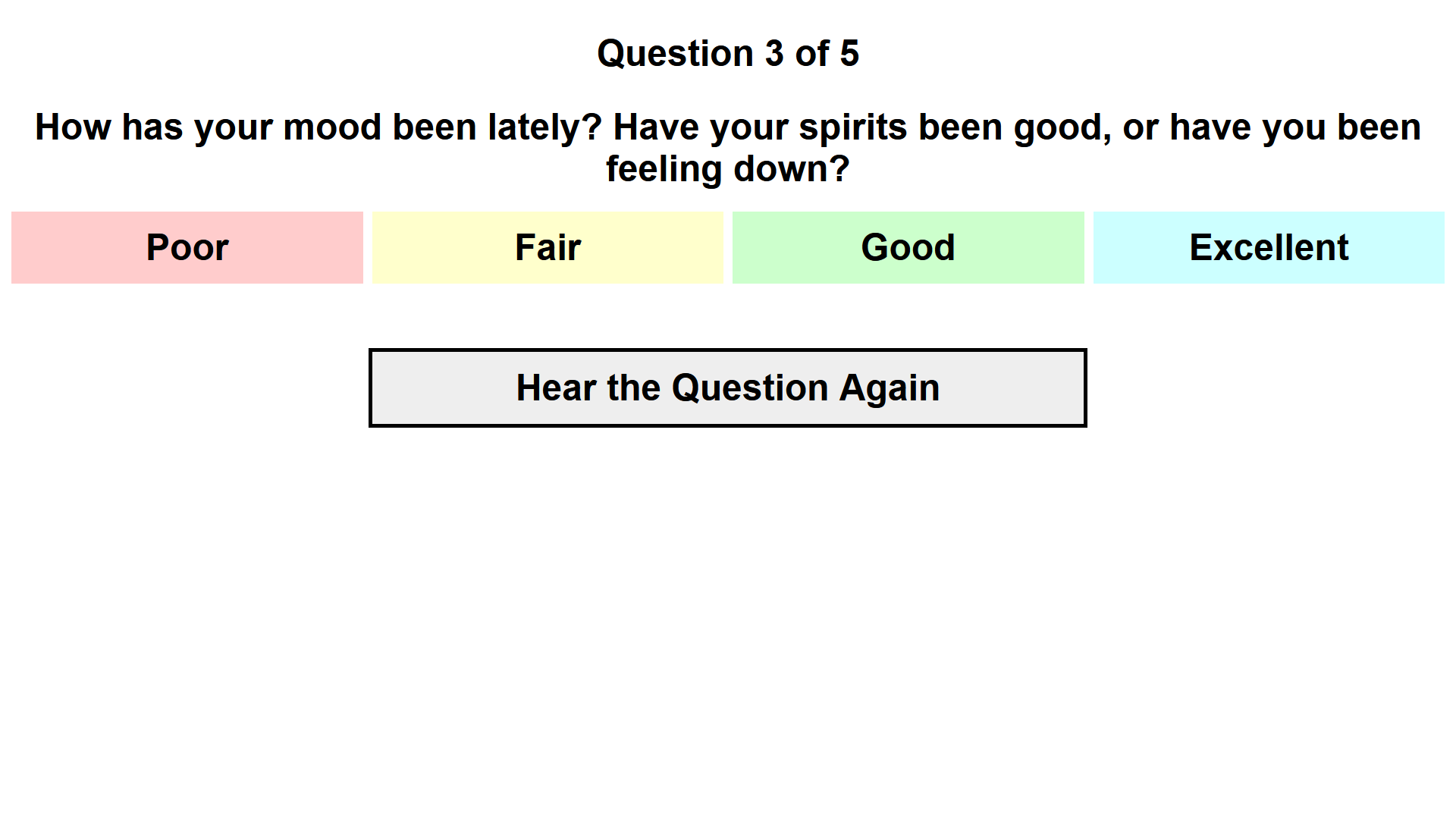
**

*Question is asked verbally & visually*

### **Figure S19.** Tracking.

**
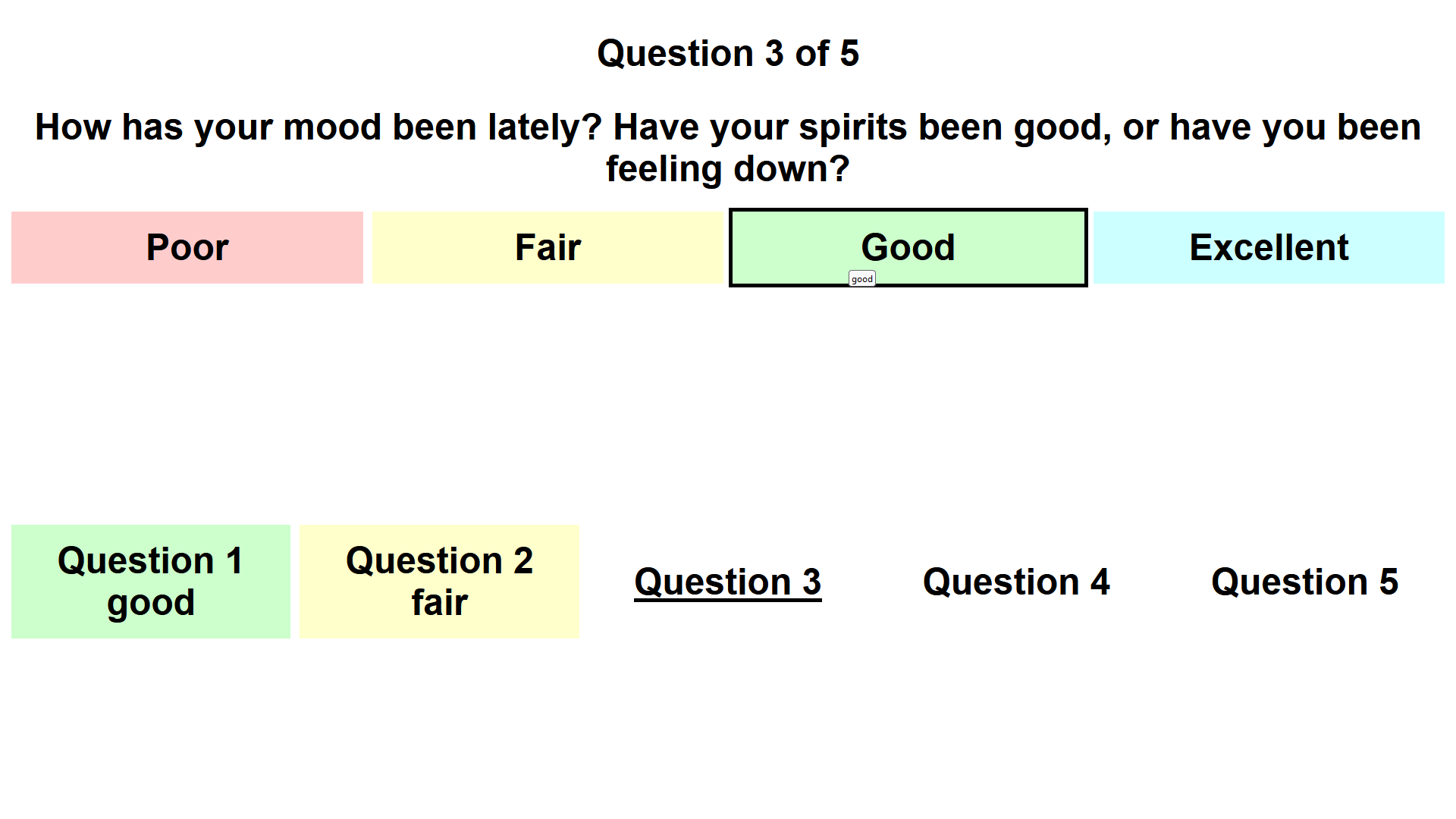
**

*Previous responses are displayed at the bottom*

### **Figure S20.** Zoom.

**
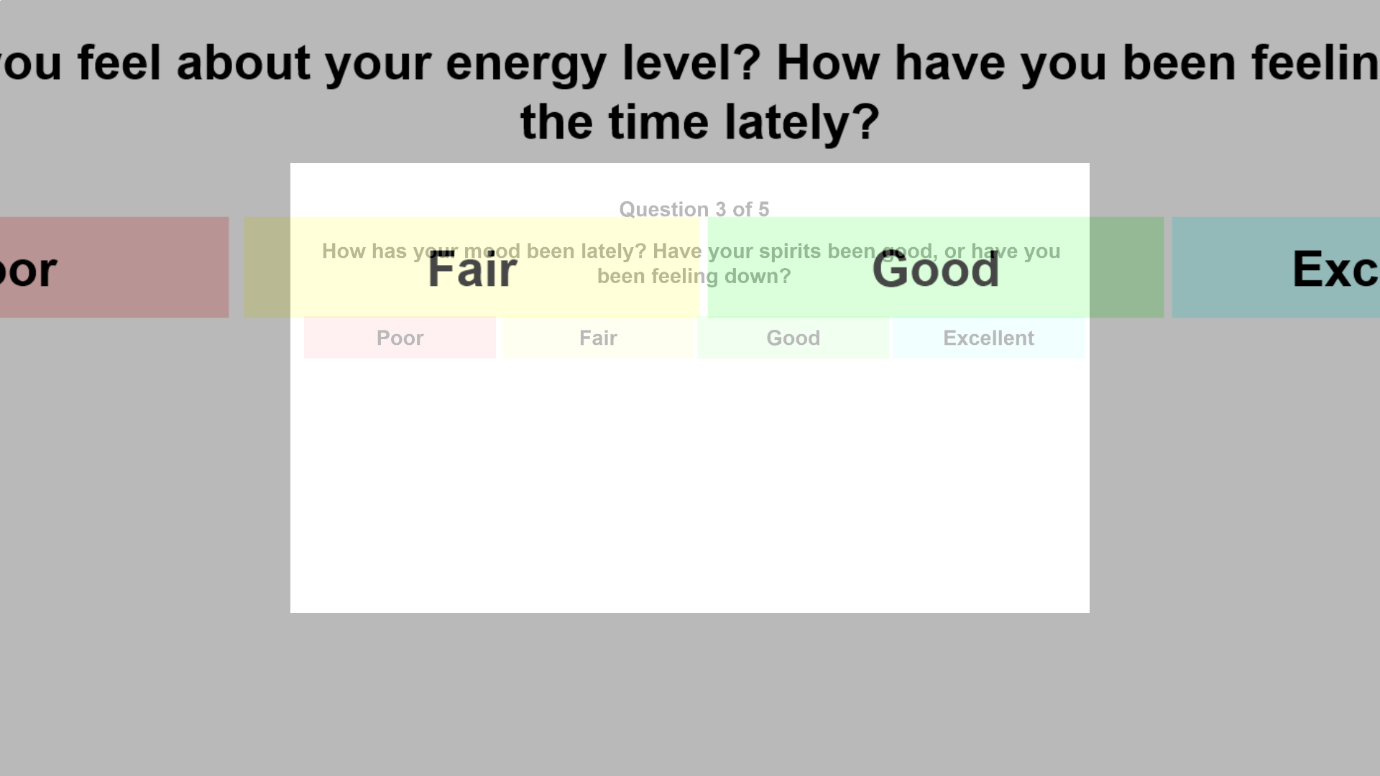
**

*zoom out transition between each question*

### **Figure S21.** Pan.

**
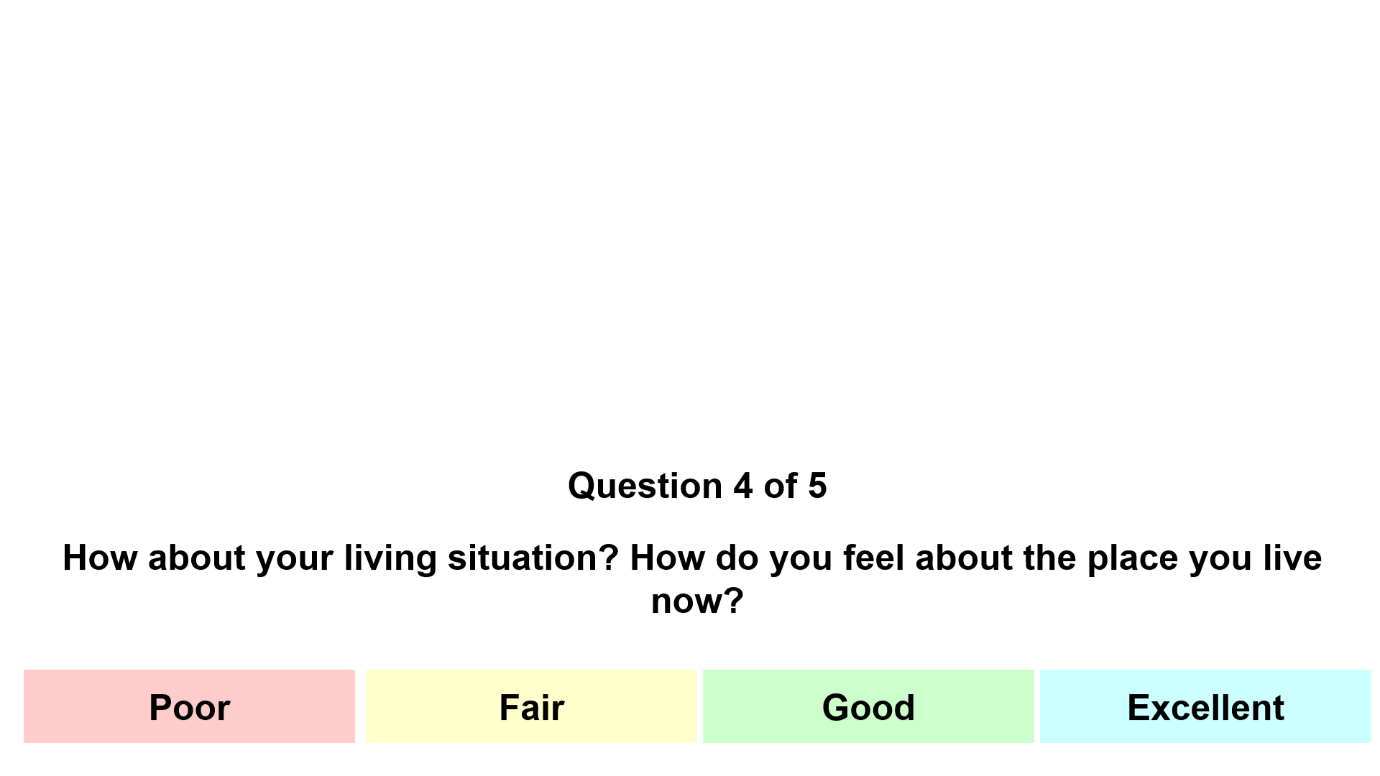
**

*Slide up (pan) transition between each question*

### **Figure S22.** Answers.

**
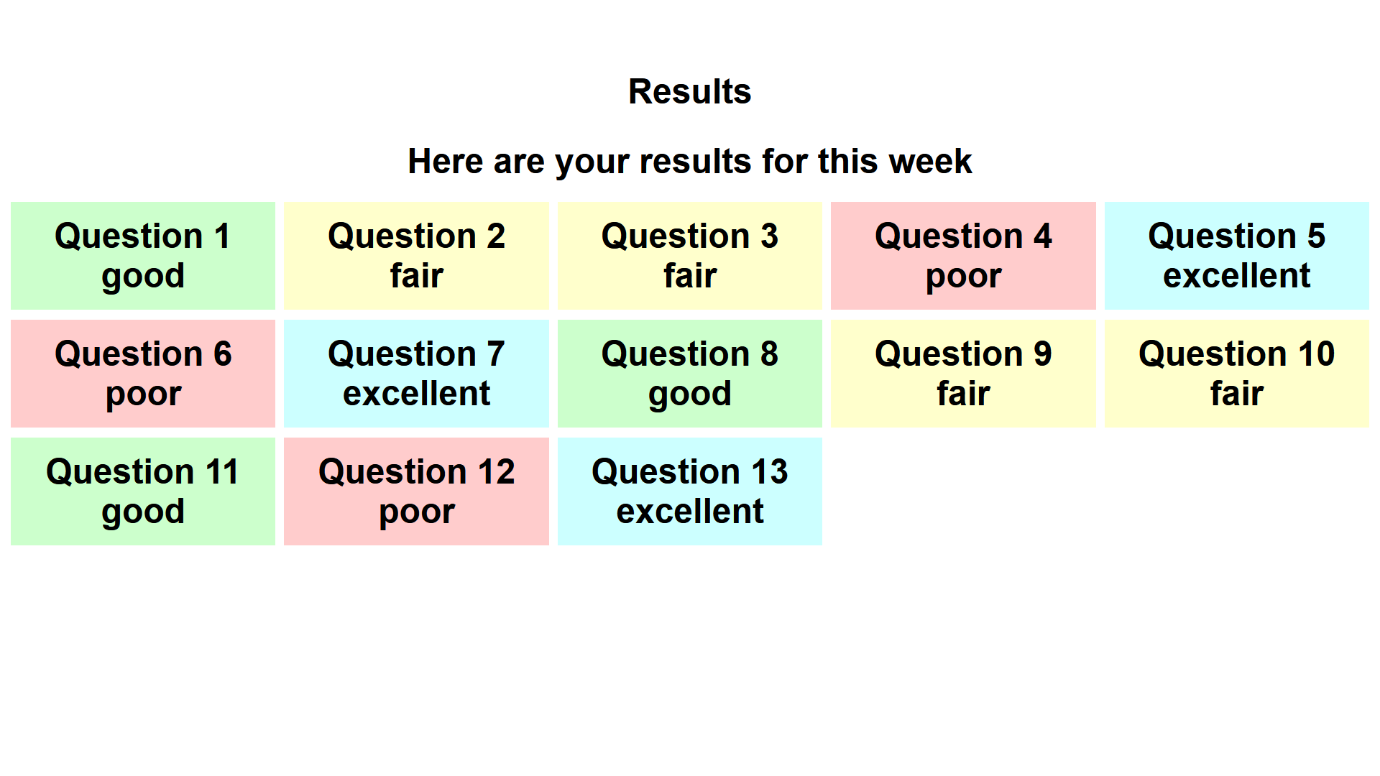
**

### **Figure S23.** Aggregated.

**
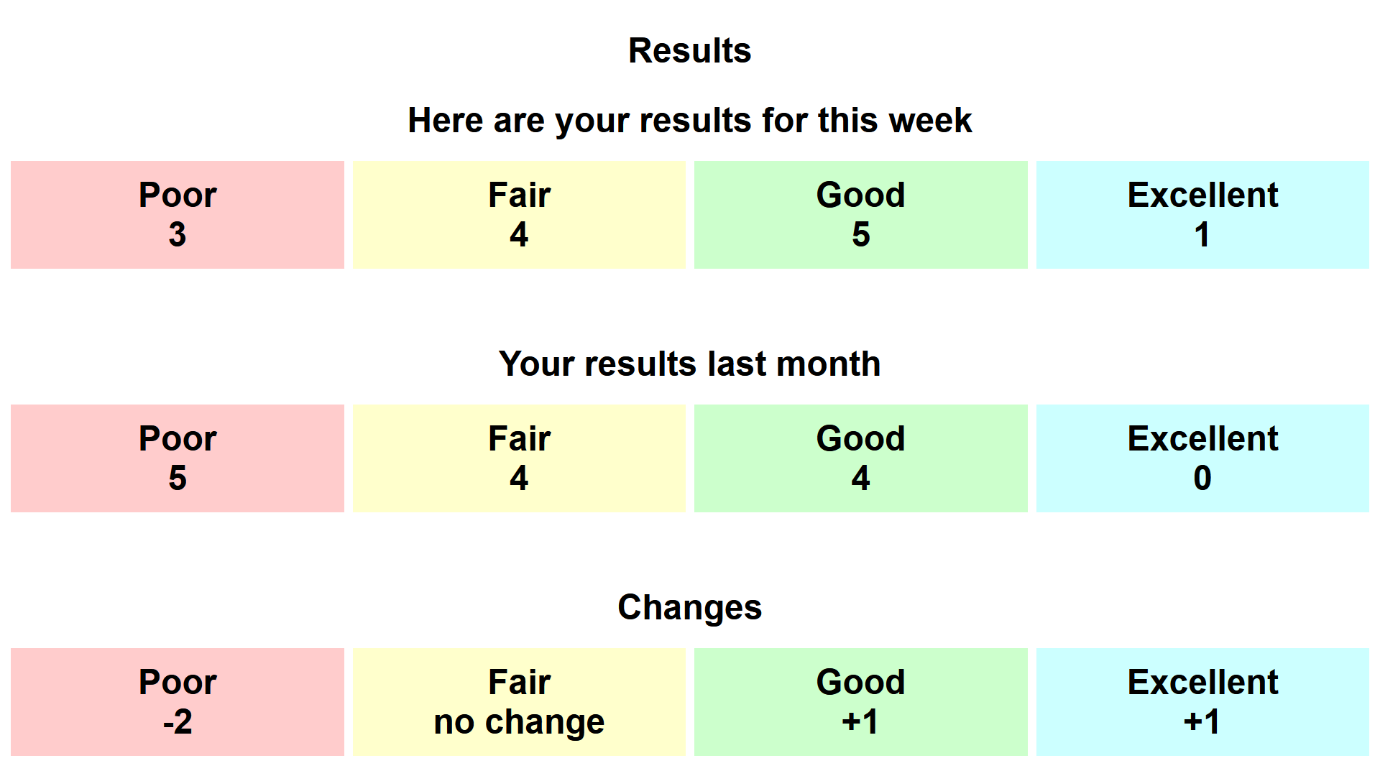
**

### **Figure S24.** Listed.

**
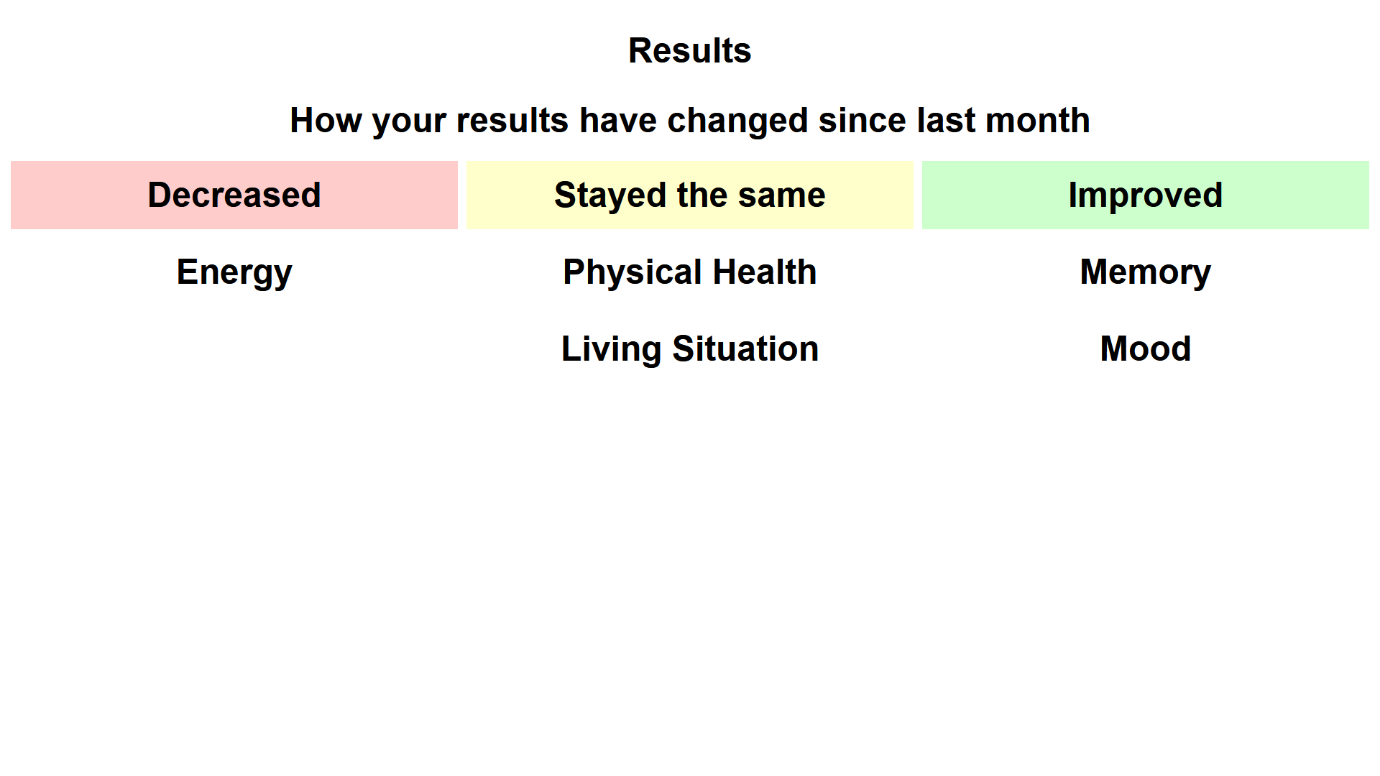
**

### **Figure S25.** Chart.

**
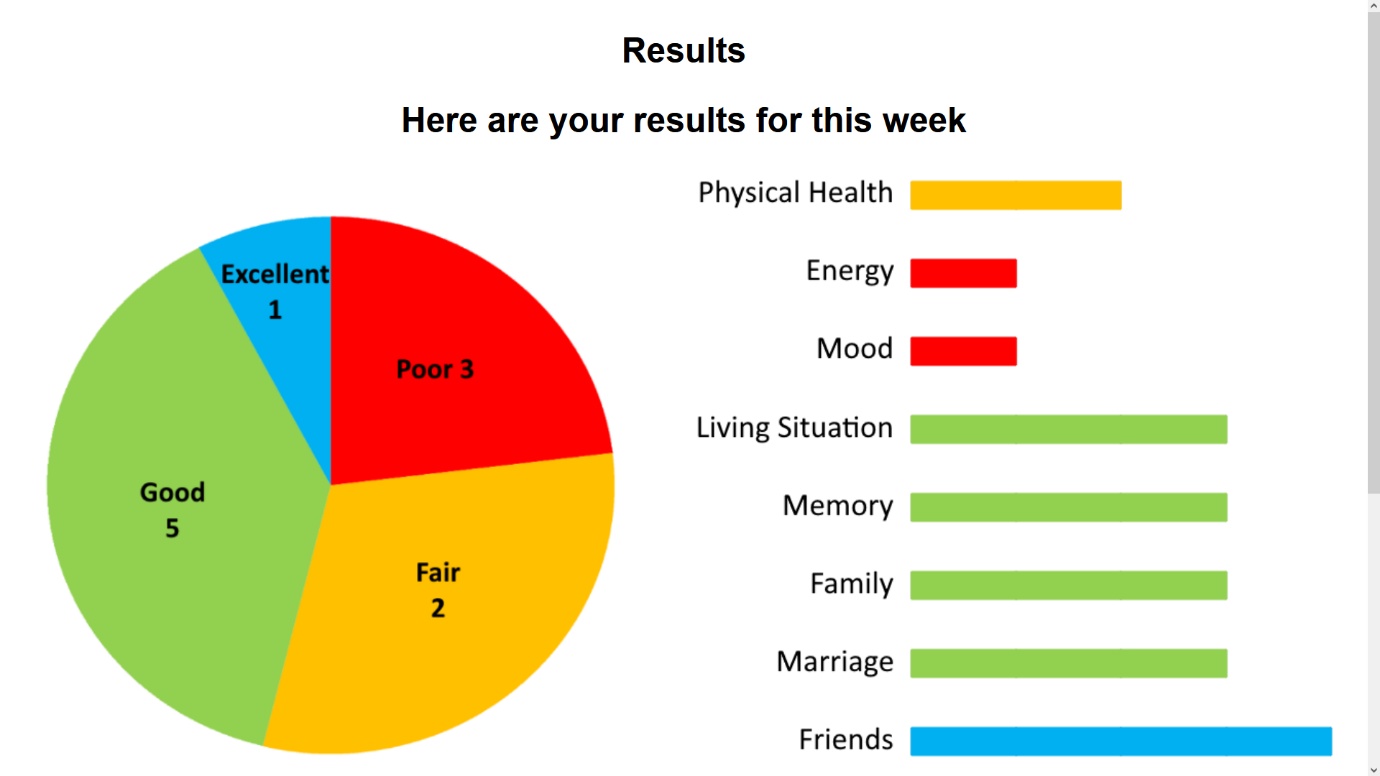
**

### **Figure S26.** Line.

**
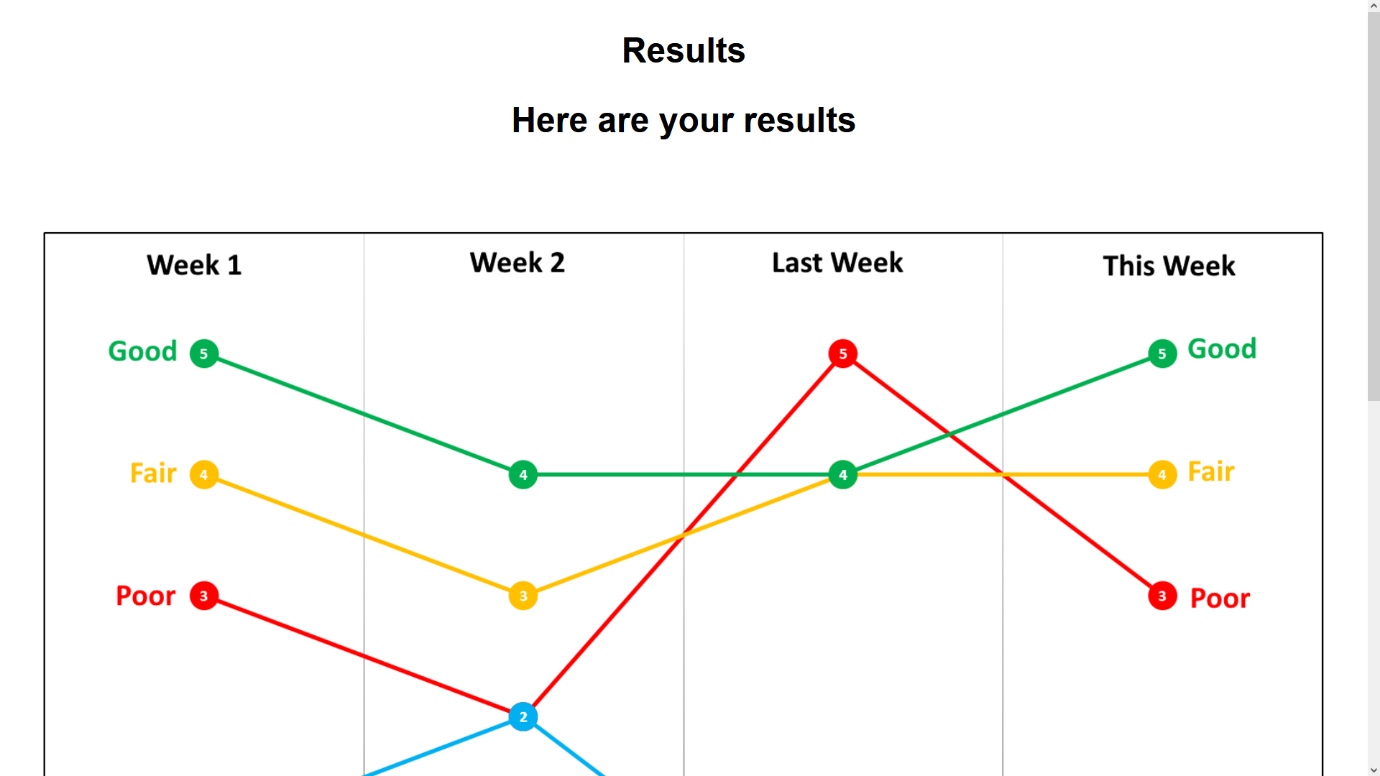
**

### **Figure S27.** Share.

**
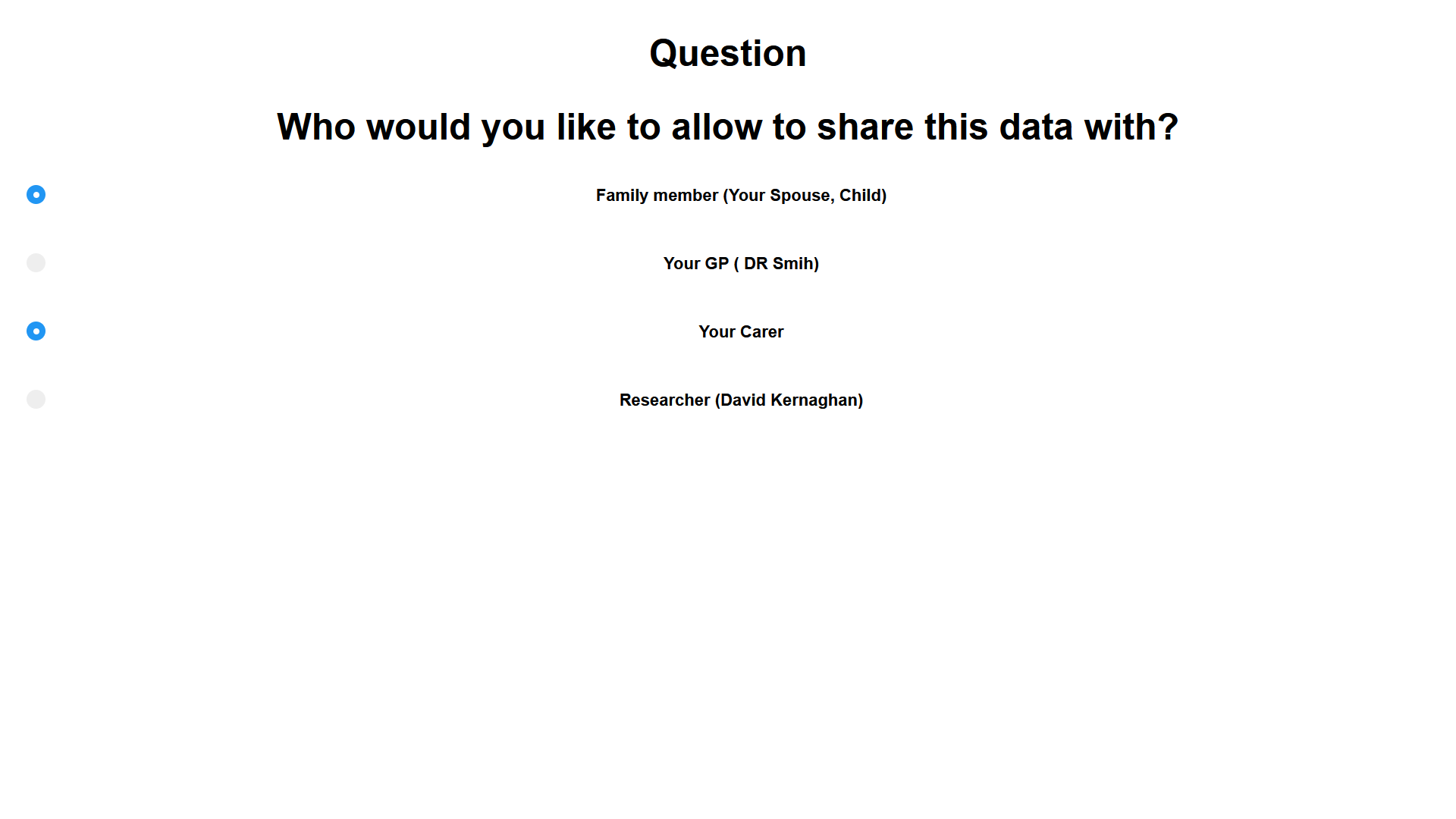
**

## Phase 3 Prototypes

### **Figure S28.** Questions.

**
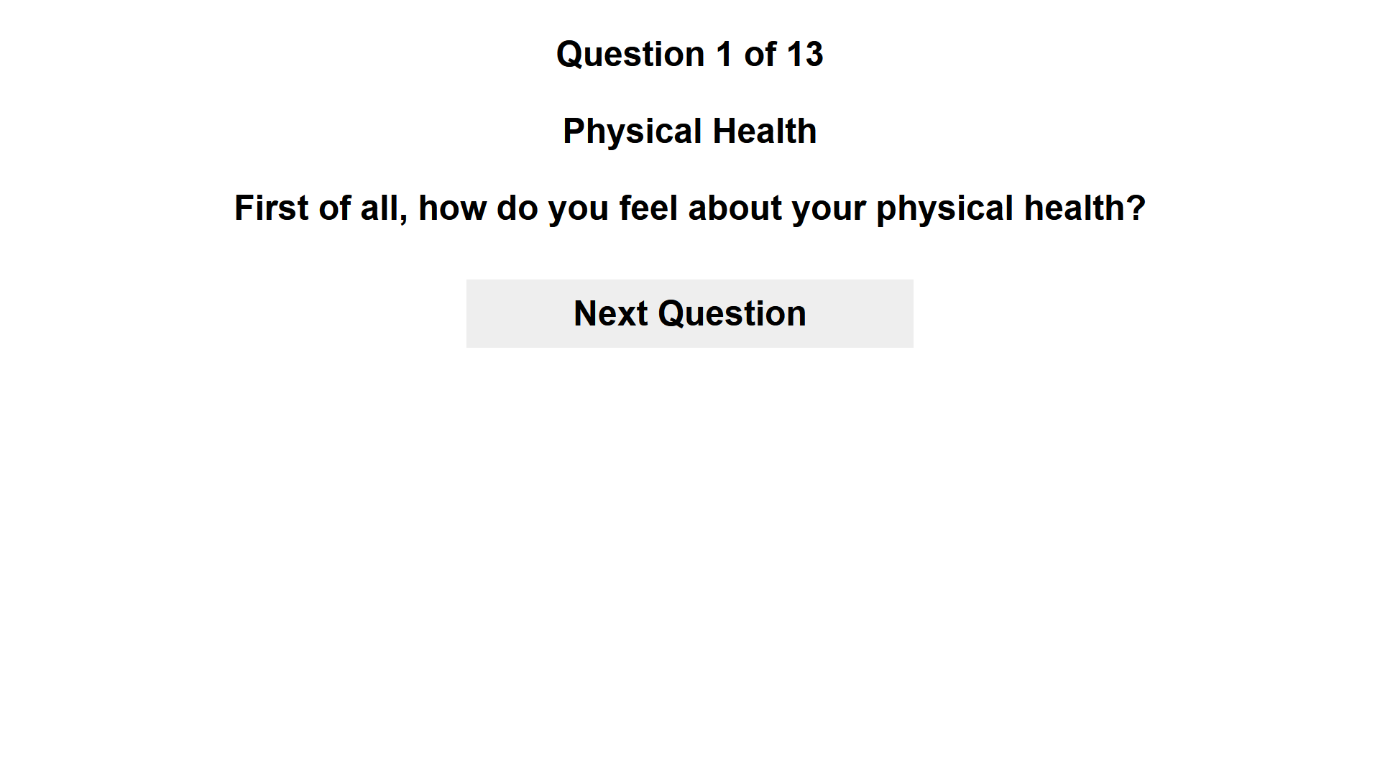
**

### **Figure S29.** Questions variants.

**
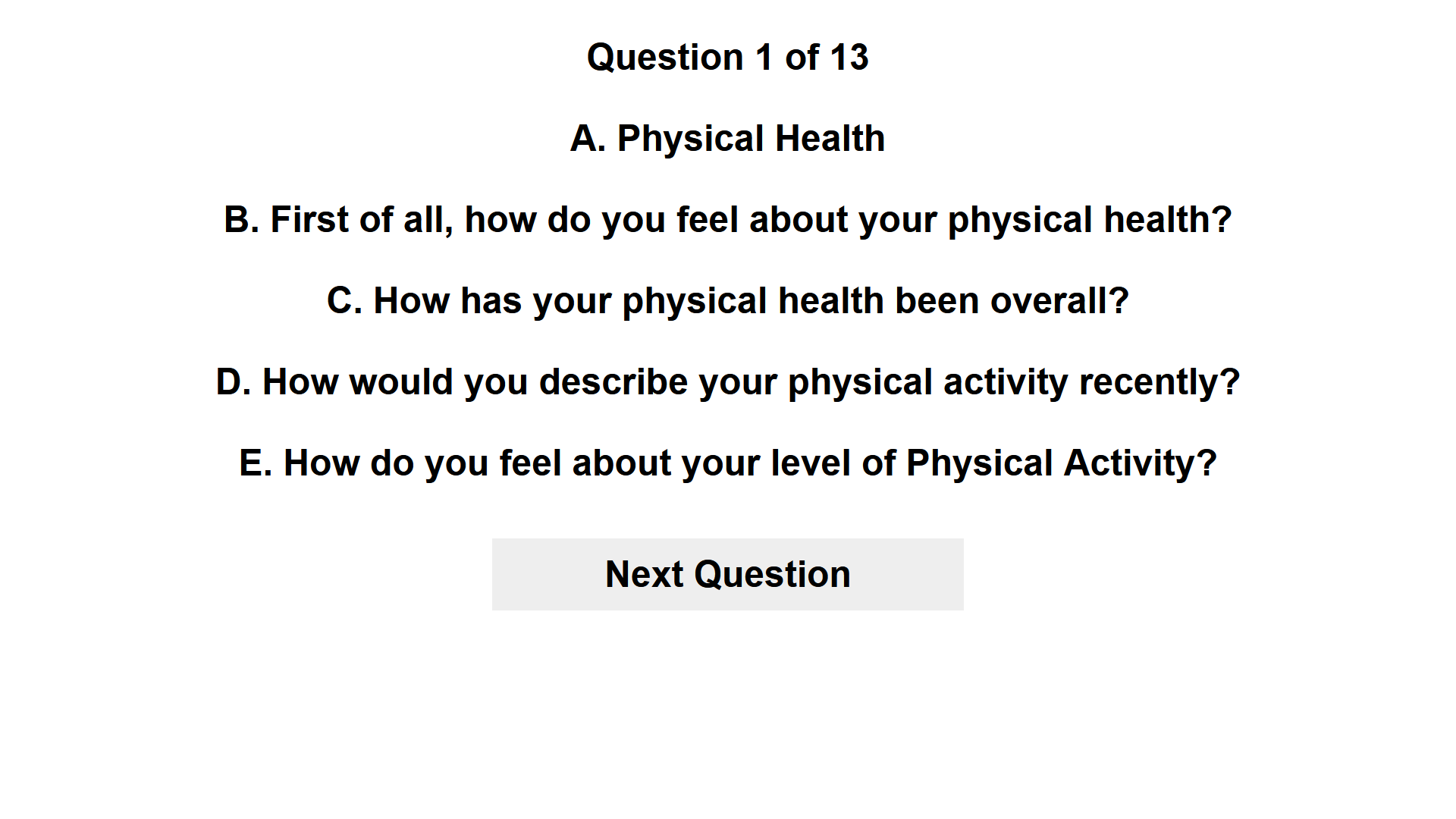
**

### **Figure S30.** Responses.

**
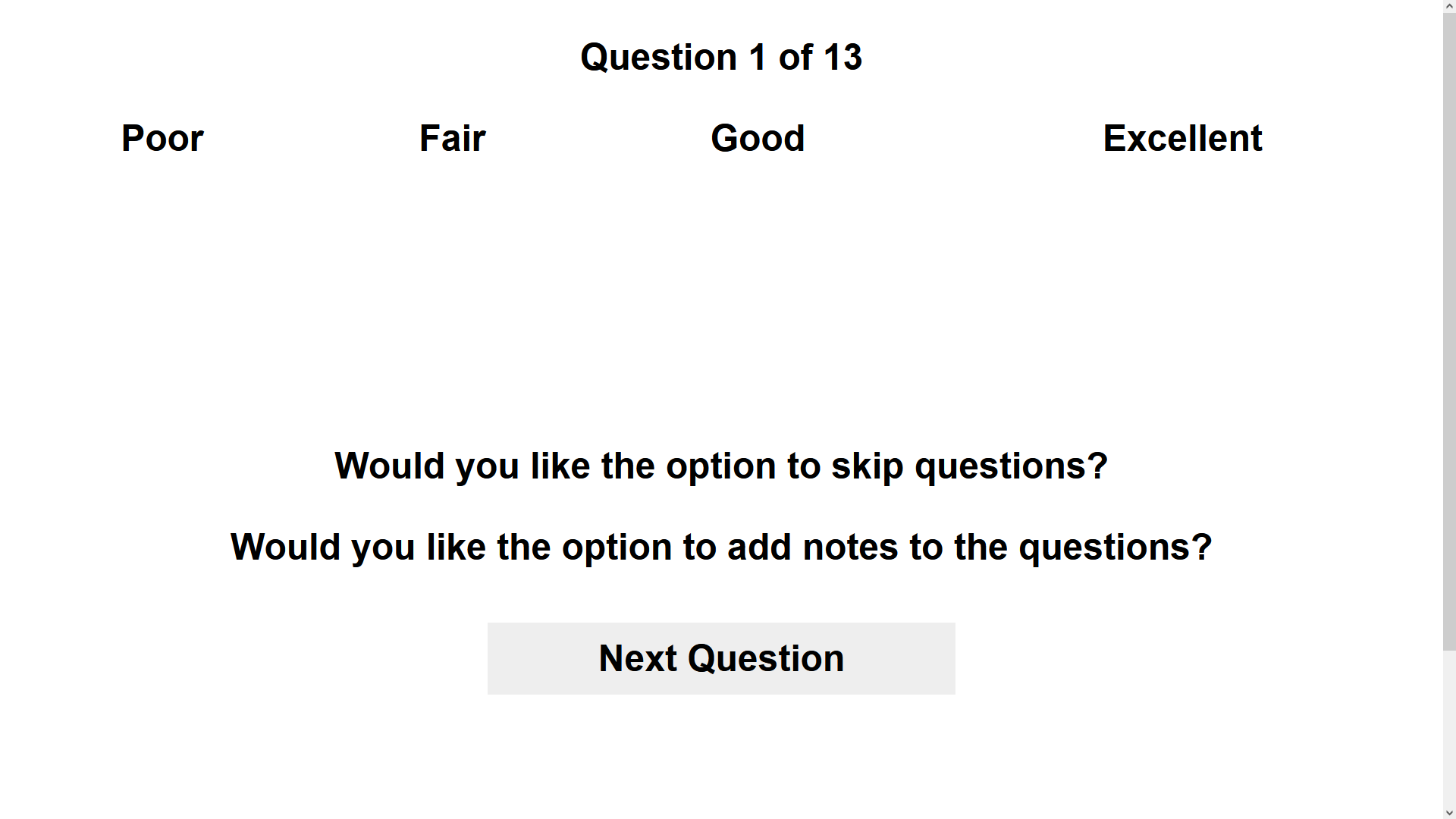
**

### **Figure S31.** Responses variants.

**
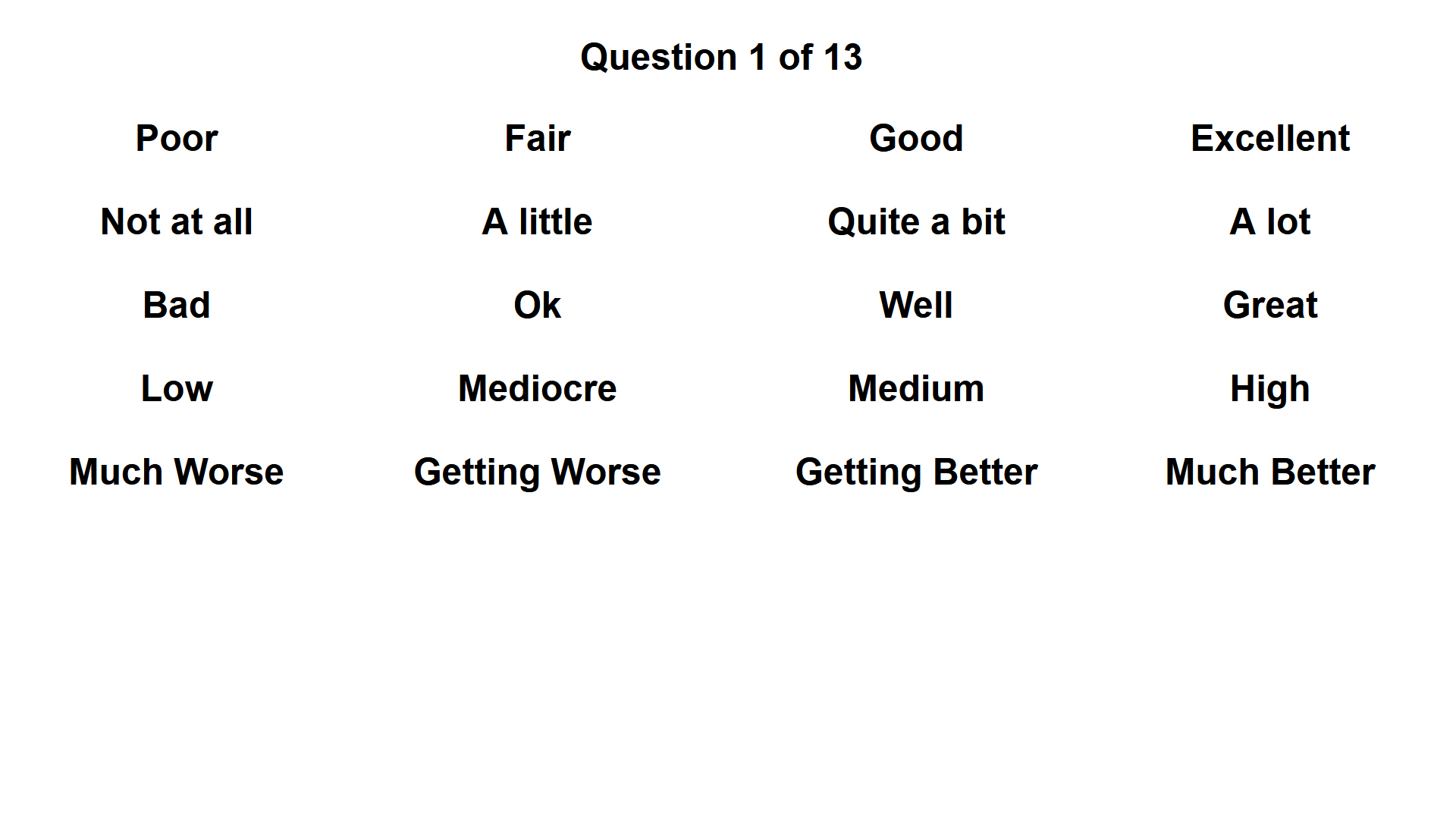
**

### **Figure S32.** Voice.

**
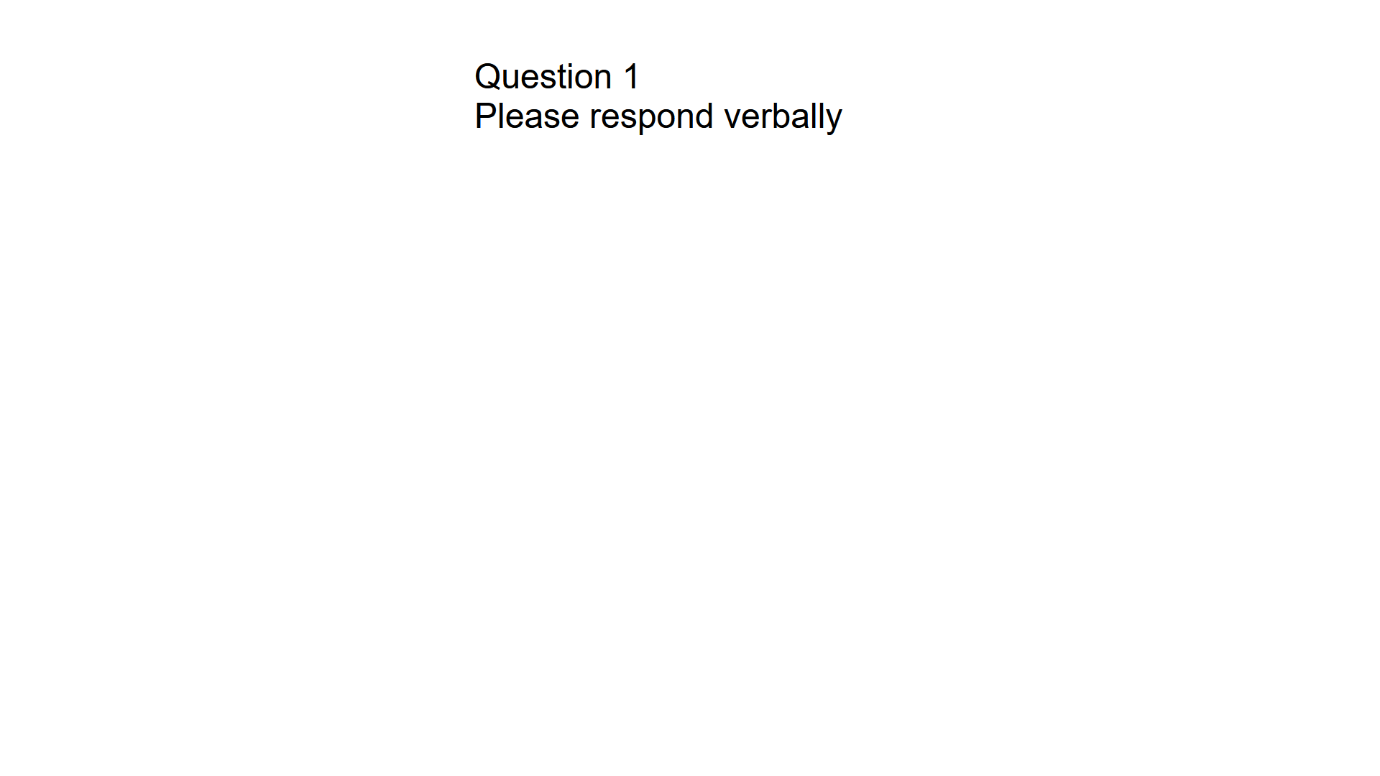
**

**Questions asked verbally:**

"First of all, how do you feel about your physical health? Would you say it’s poor, fair, good, or excellent?"

"How do you feel about your energy level? How have you been feeling most of the time lately. Do you think it is poor, fair, good, or excellent?"

"How has your mood been lately? Have your spirits been good, or have you been feeling down? Would you rate your mood as poor, fair, good, or excellent?"

### **Figure S33.** Preproduction (start page).

**
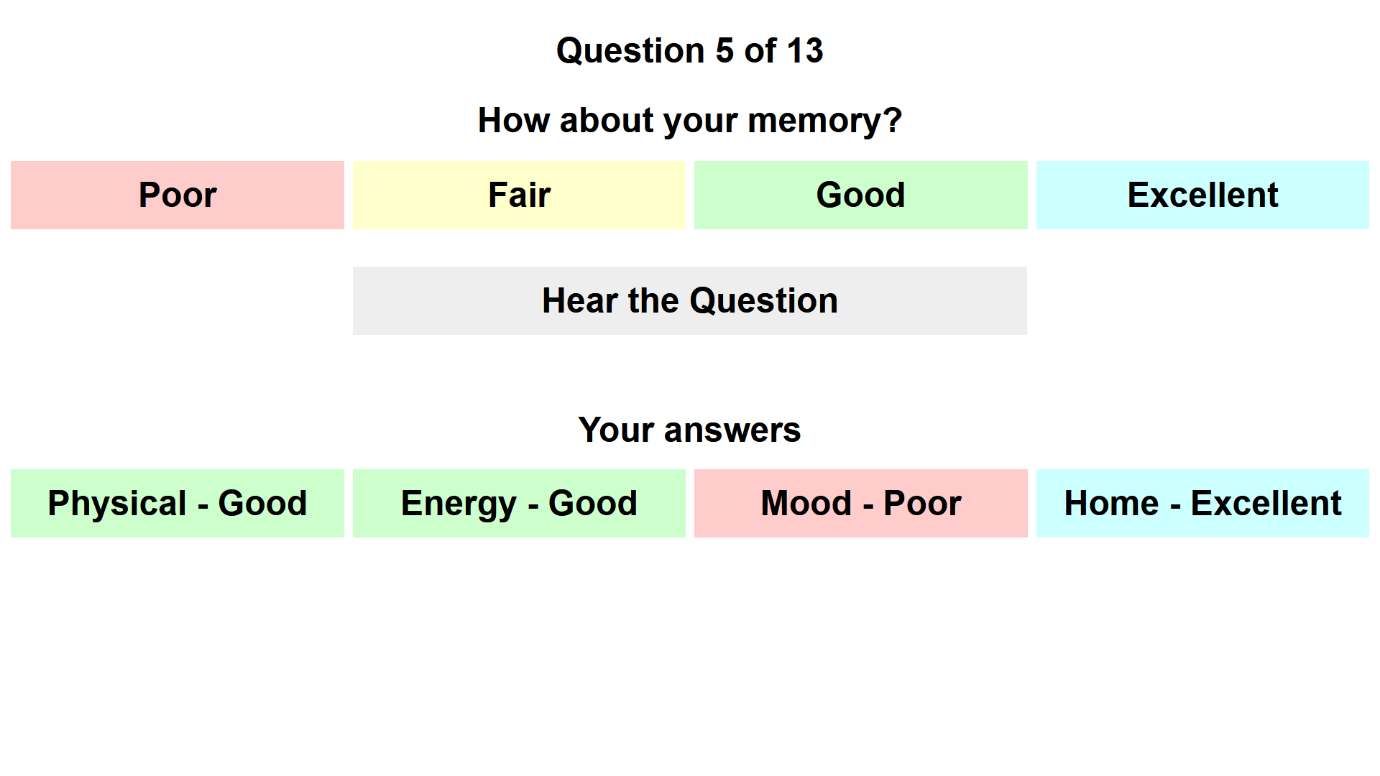
**

### **Figure S34.** Preproduction (questions page).

**
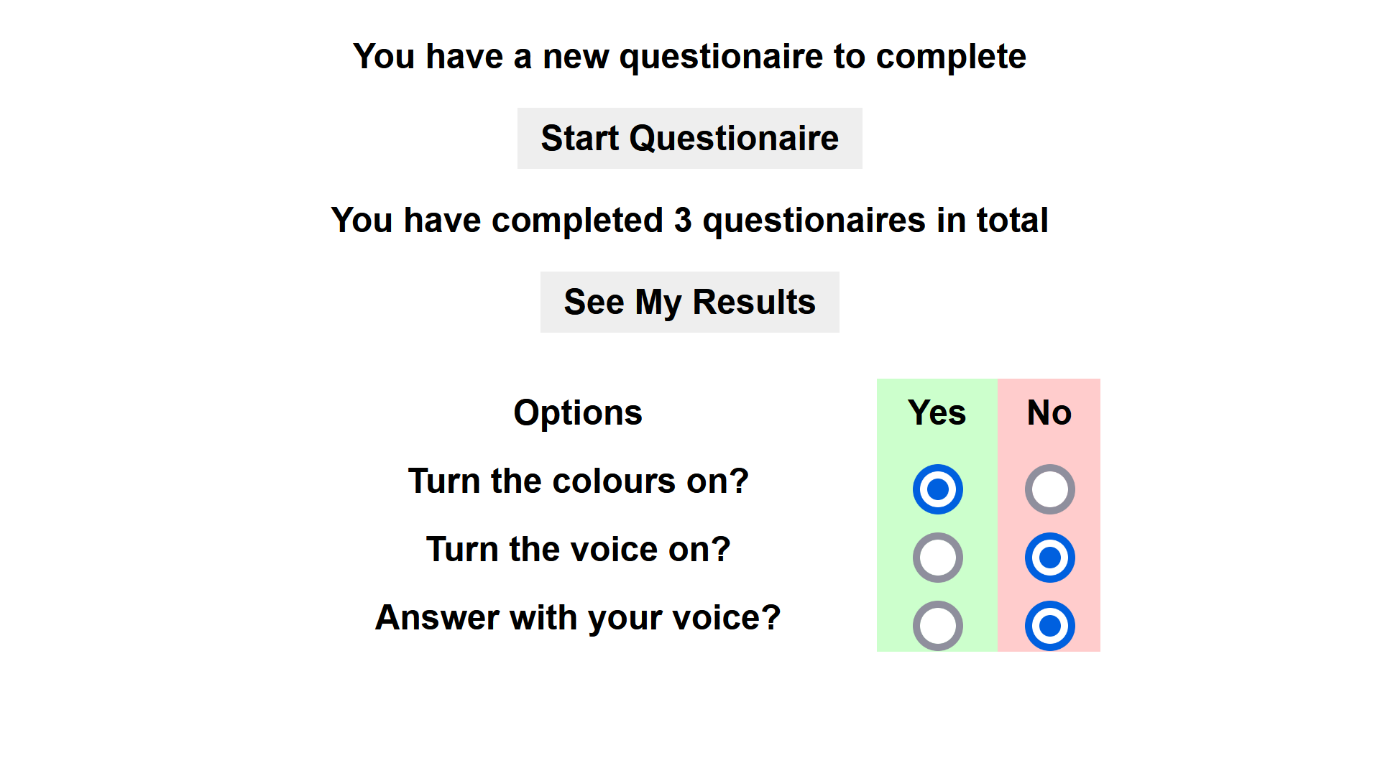
**

### **Figure S35.** Preproduction (Results page 1).

**
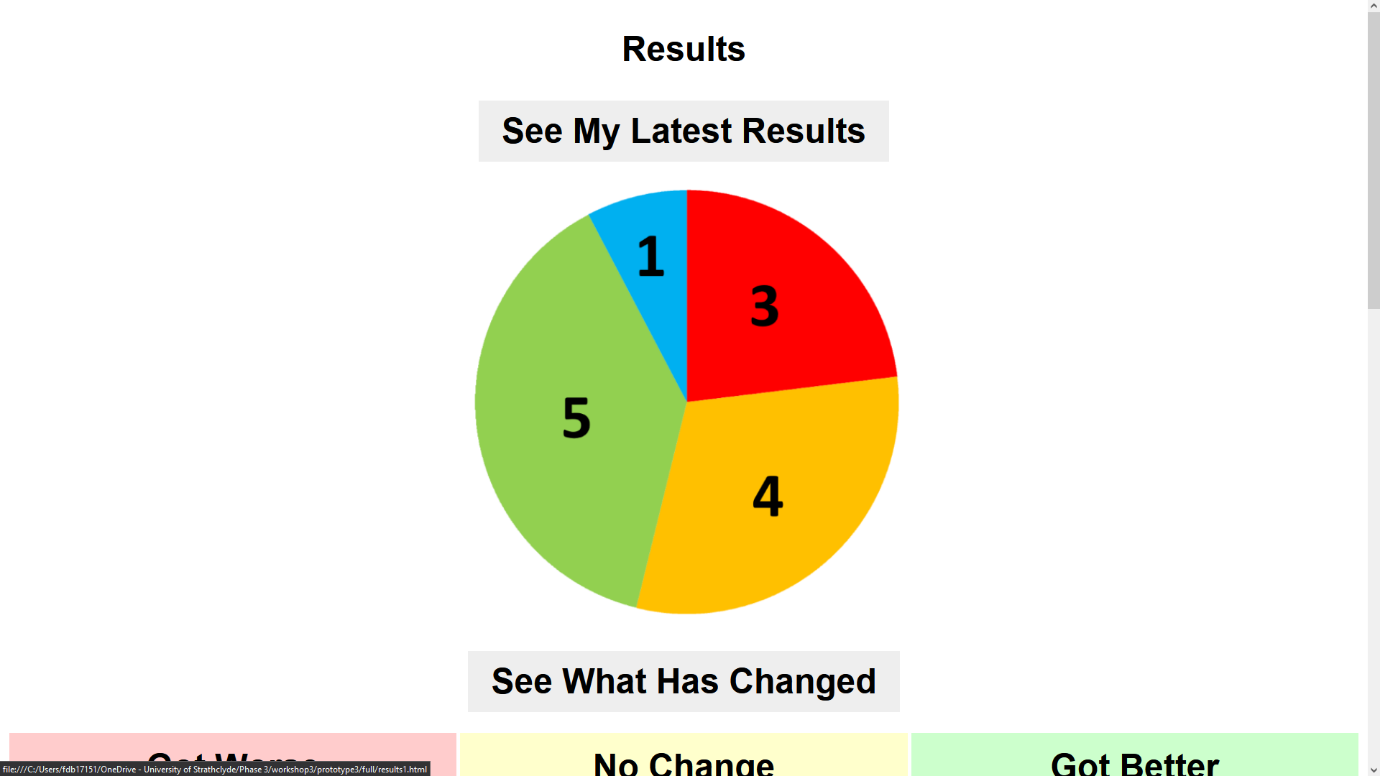
**

### **Figure S36.** Preproduction (Results Page 2).

**
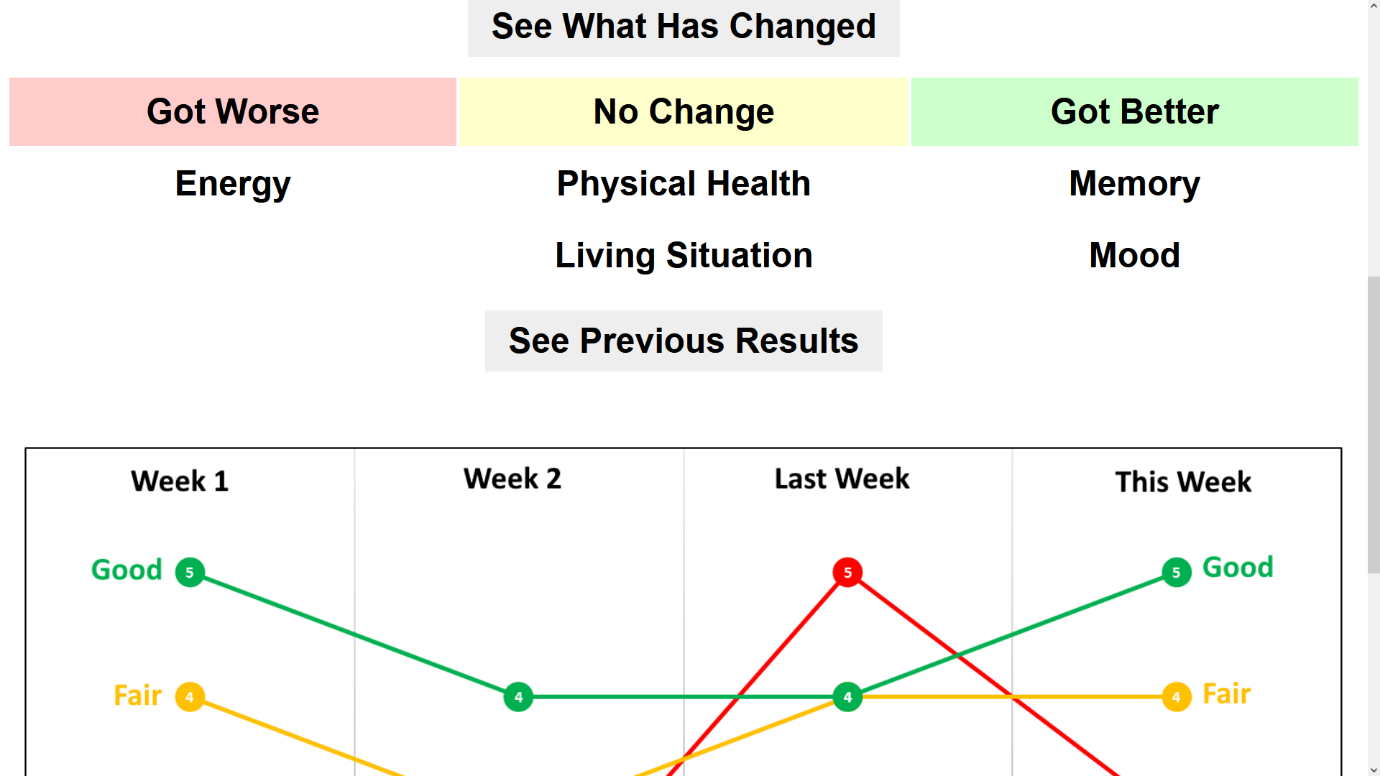
**

### **Figure S37.** Preproduction (share page).

**
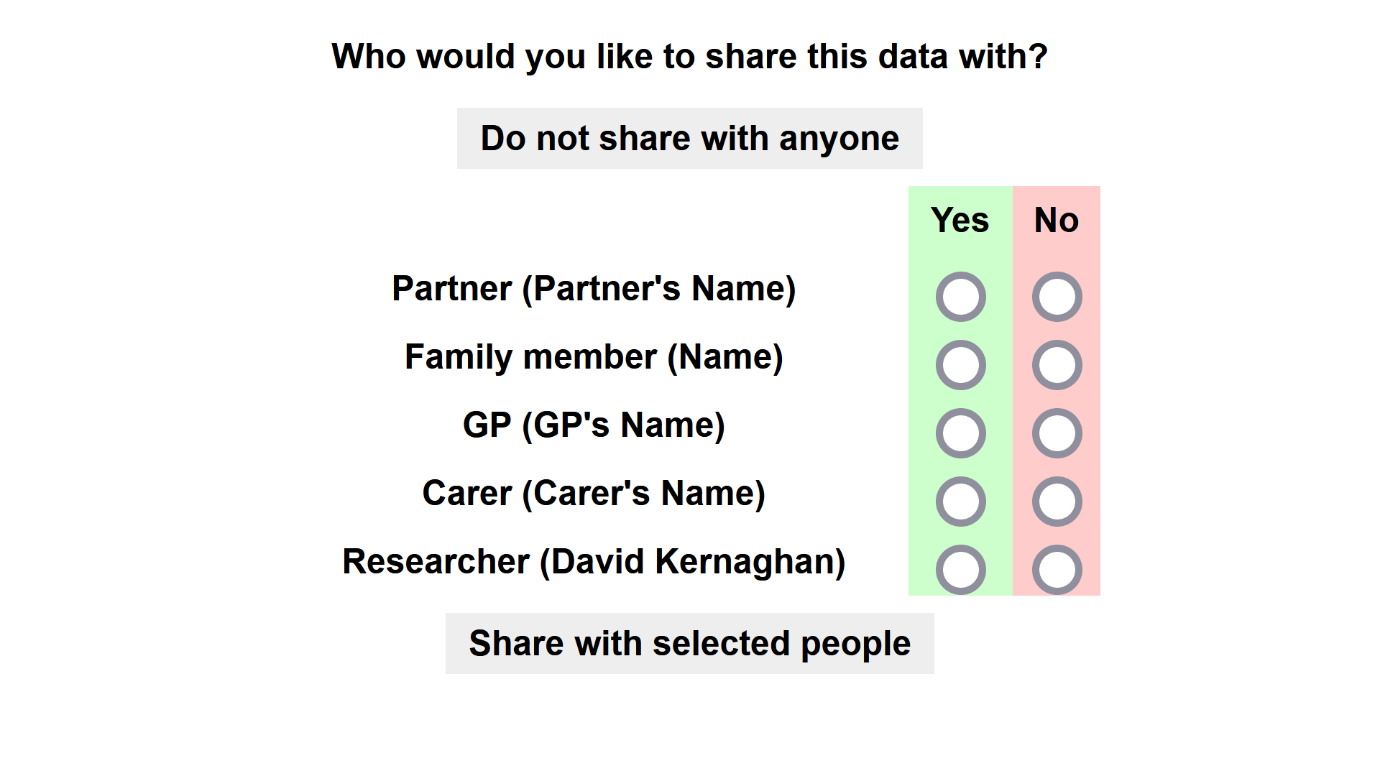
**

## Phase 4 Prototype

### **Figure S38.** Beta (start page).

**
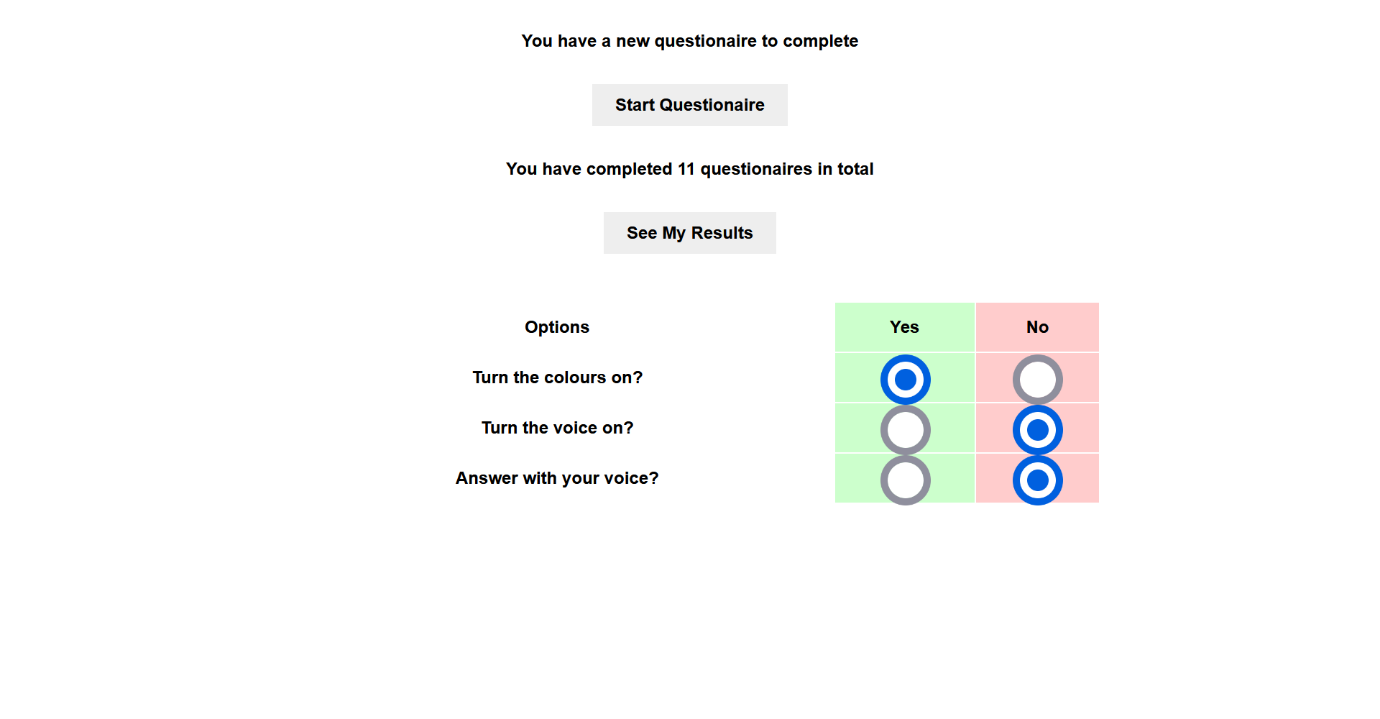
**

### **Figure S39.** Beta (question 1).

**
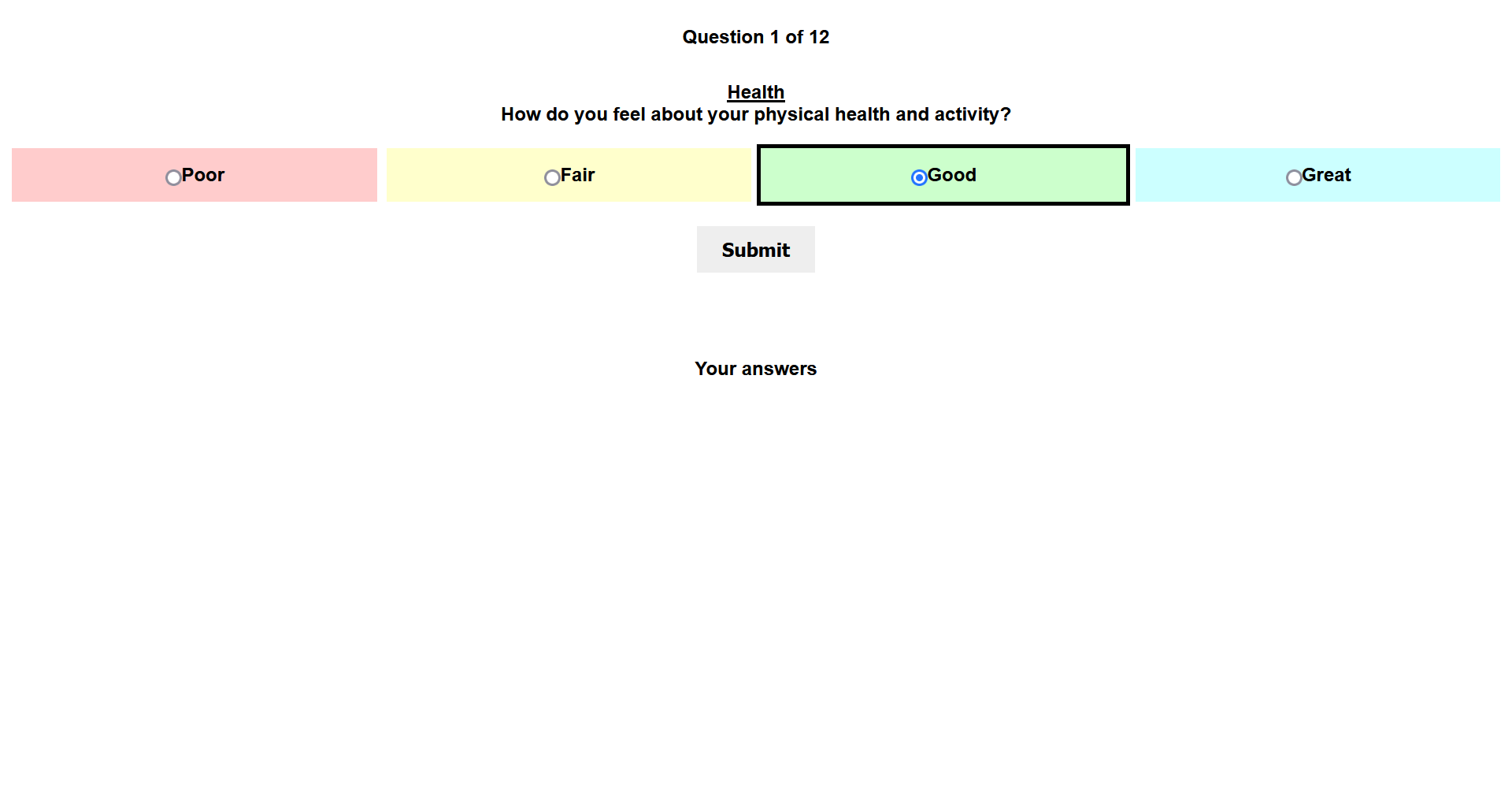
**

### **Figure S40.** Beta (final question).

**
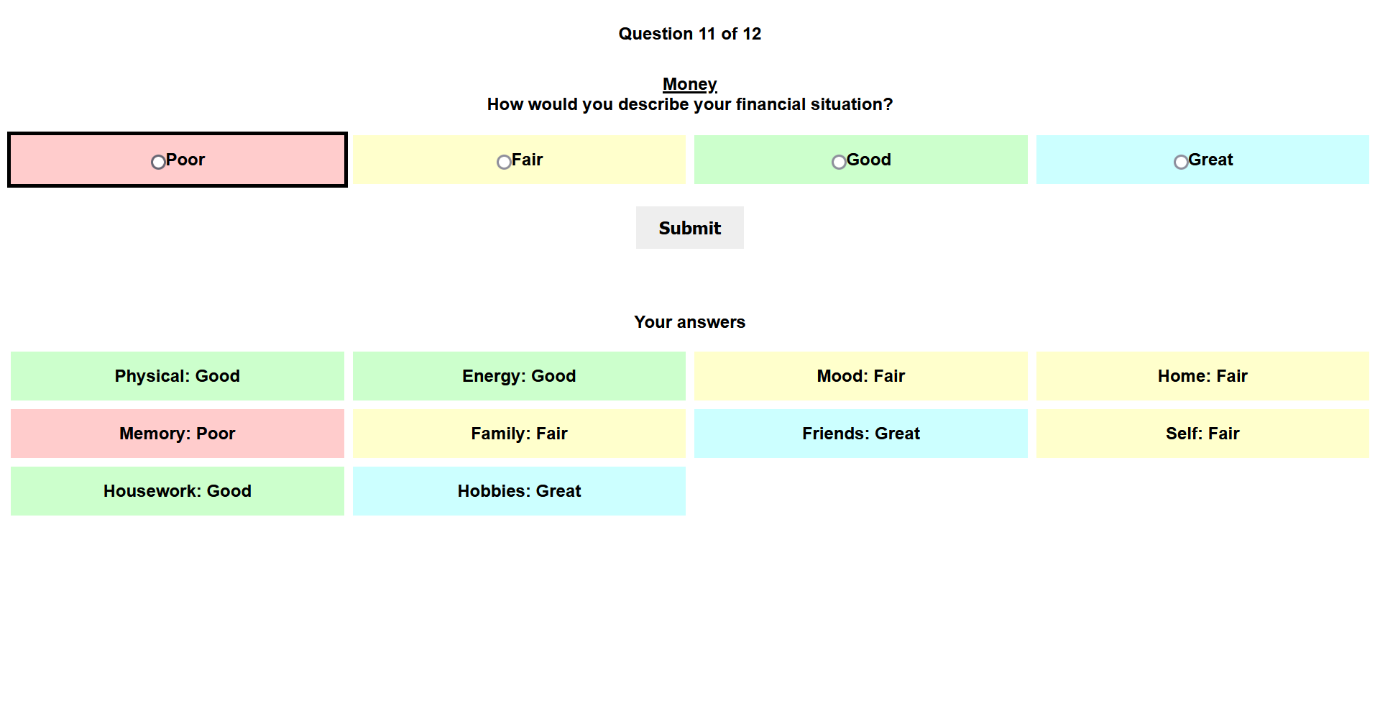
**

### **Figure S41.** Beta (review responses).

**
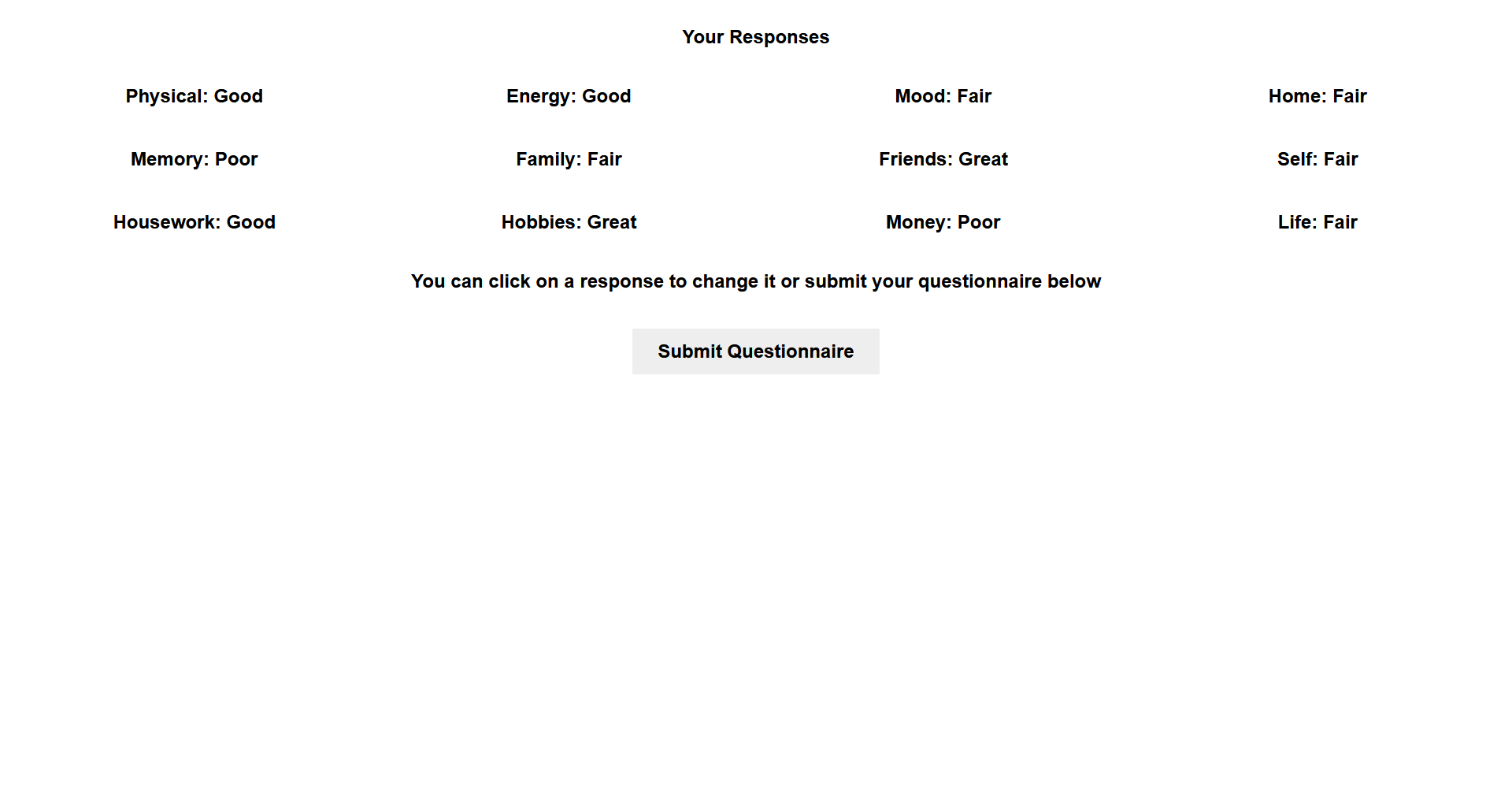
**

### **Figure S42.** Beta (share screen).

**
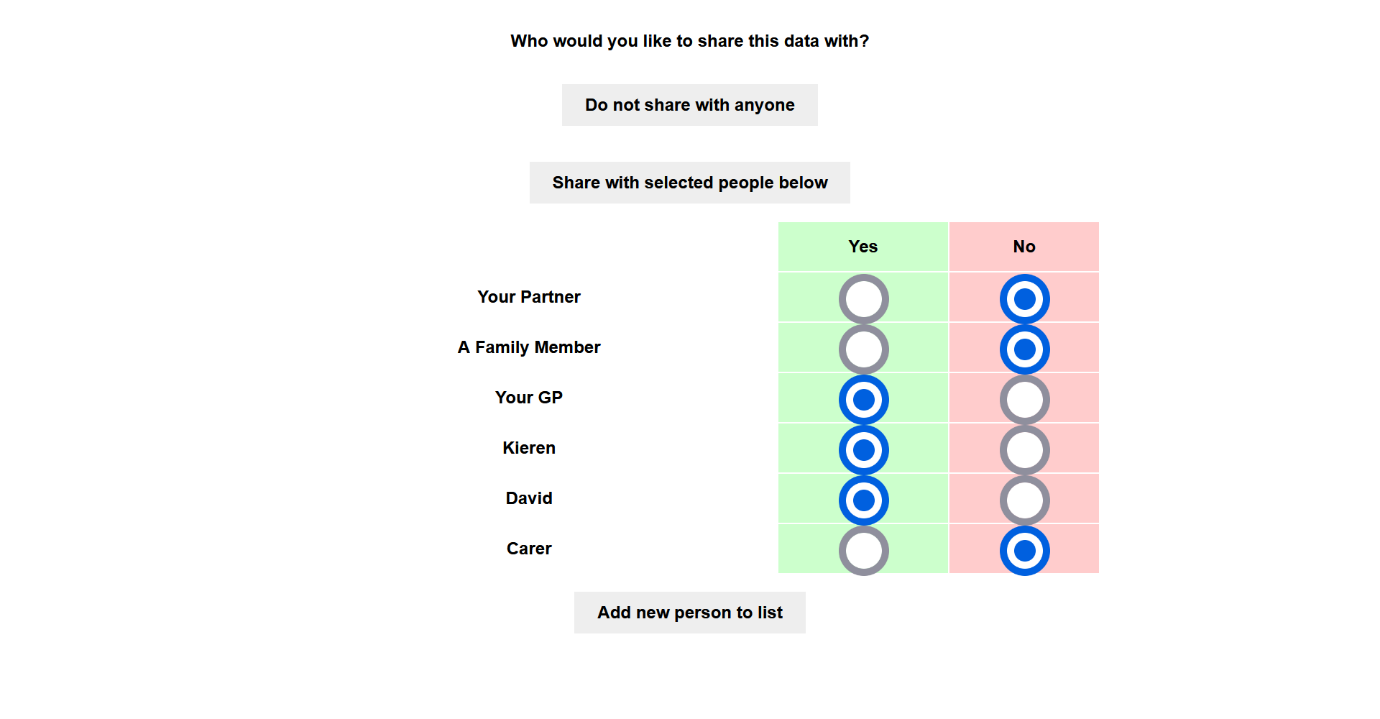
**

### **Figure S43.** Beta (add contact).

**
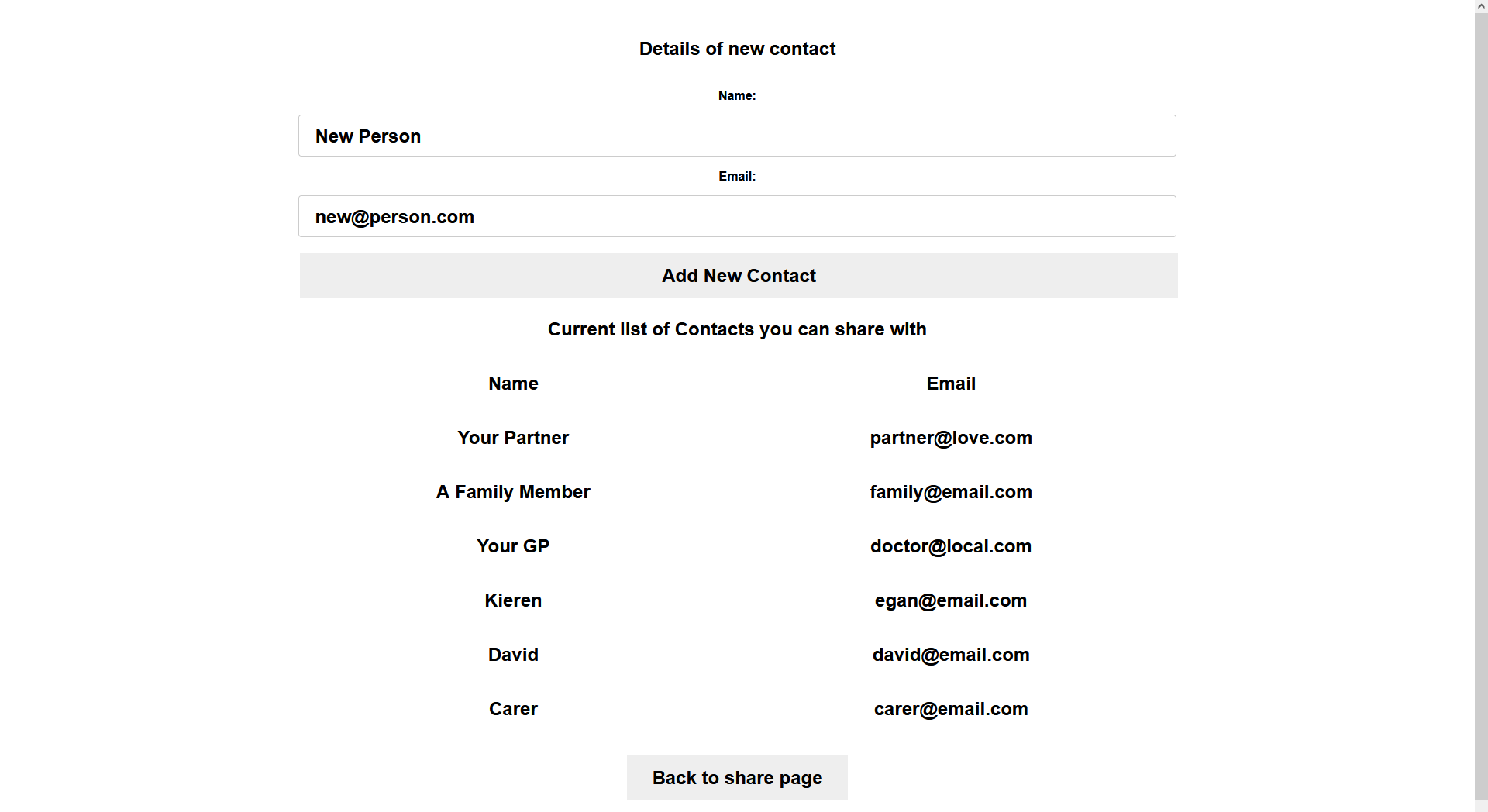
**

### **Figure S44.** Beta (submitted page).

**
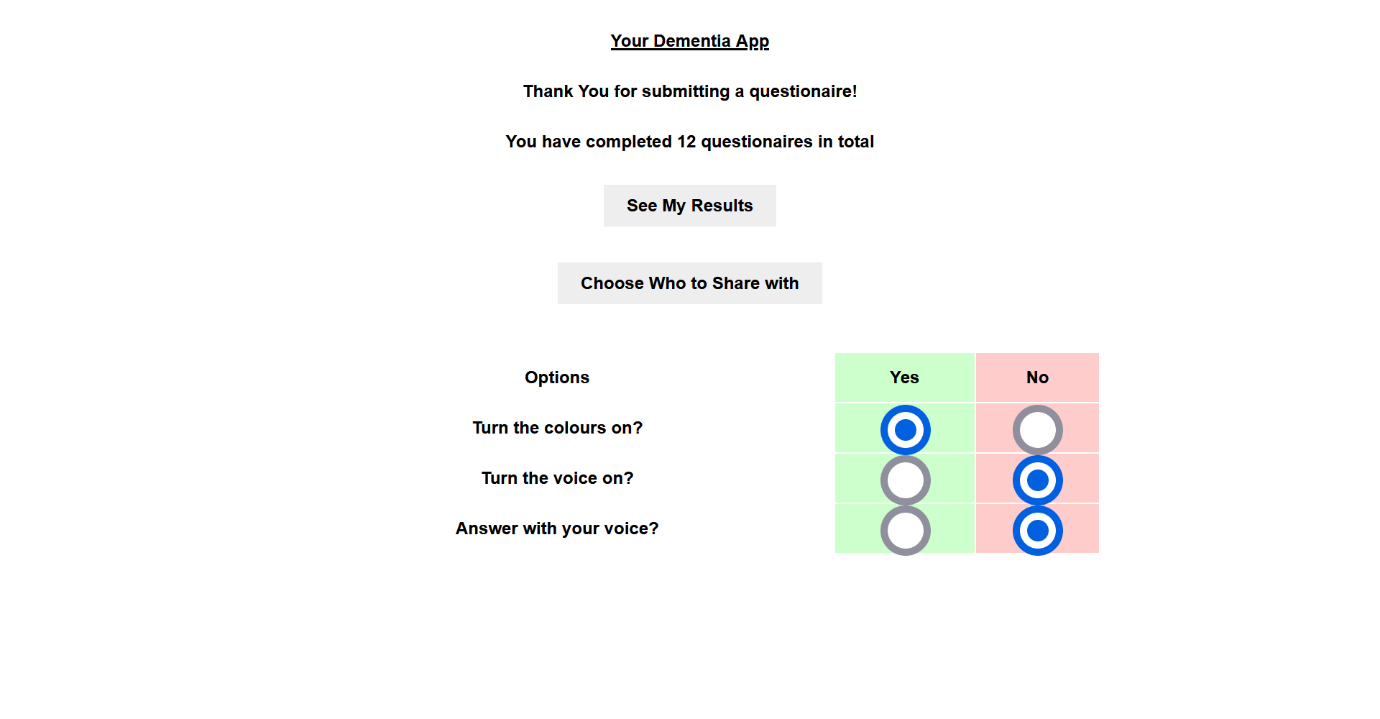
**

### **Figure S45.** Beta (latest results).

**
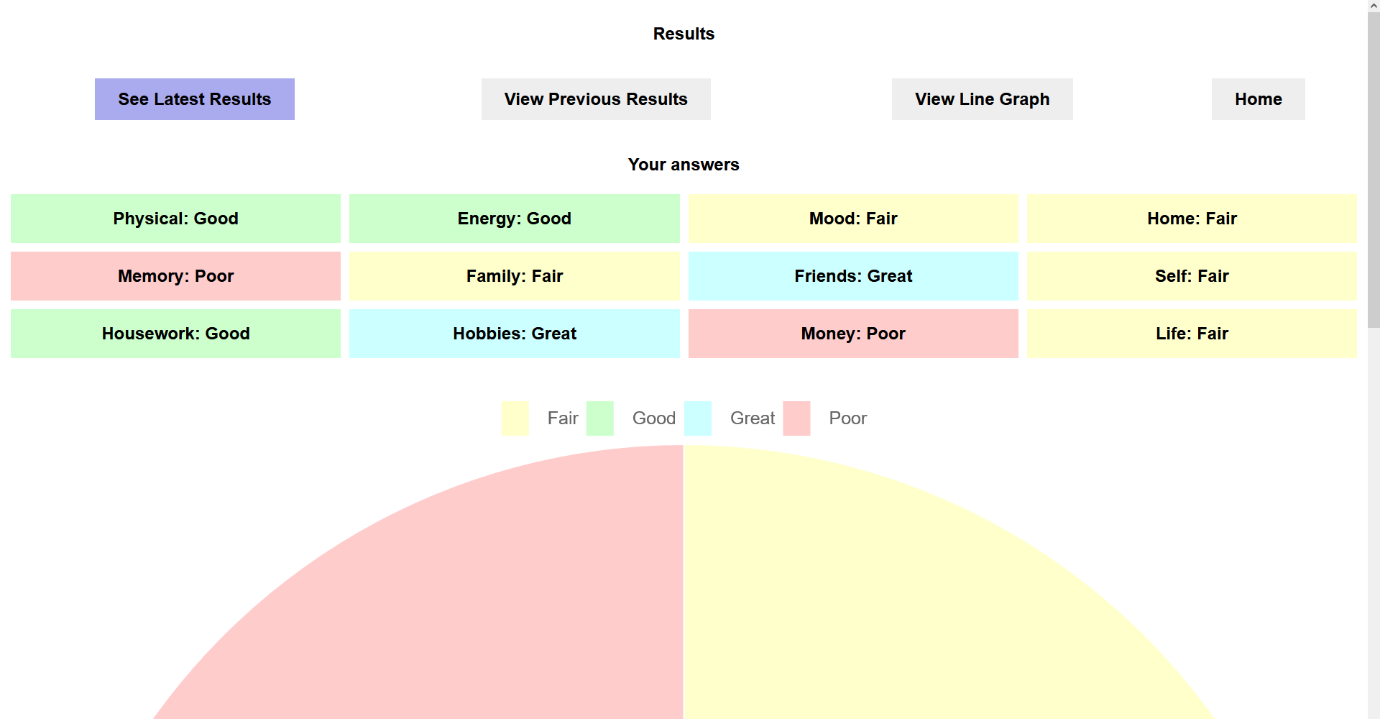
**

### **Figure S46.** Beta (previous results).

**
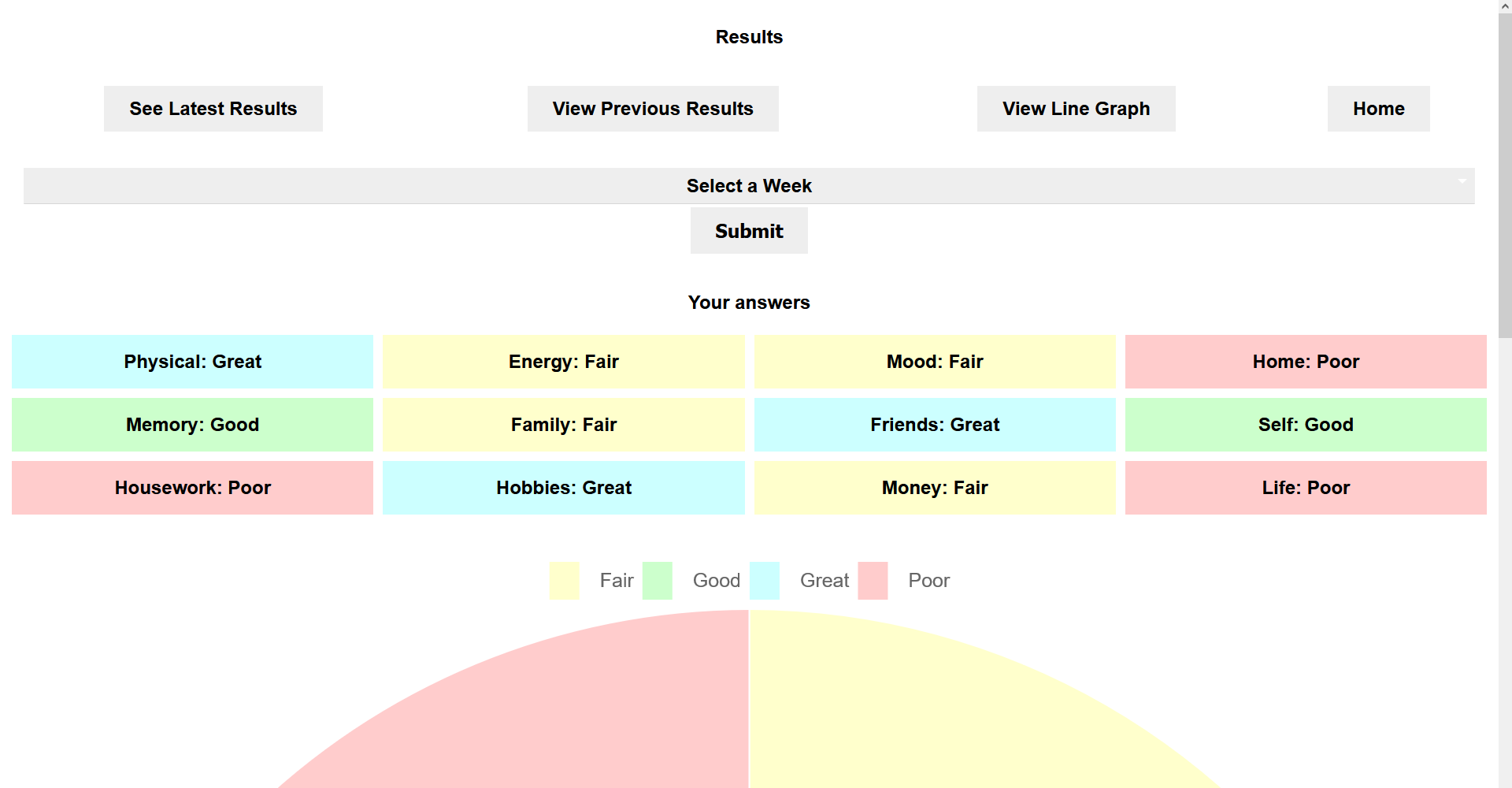
**

### **Figure S47.** Beta (line graph results).

**
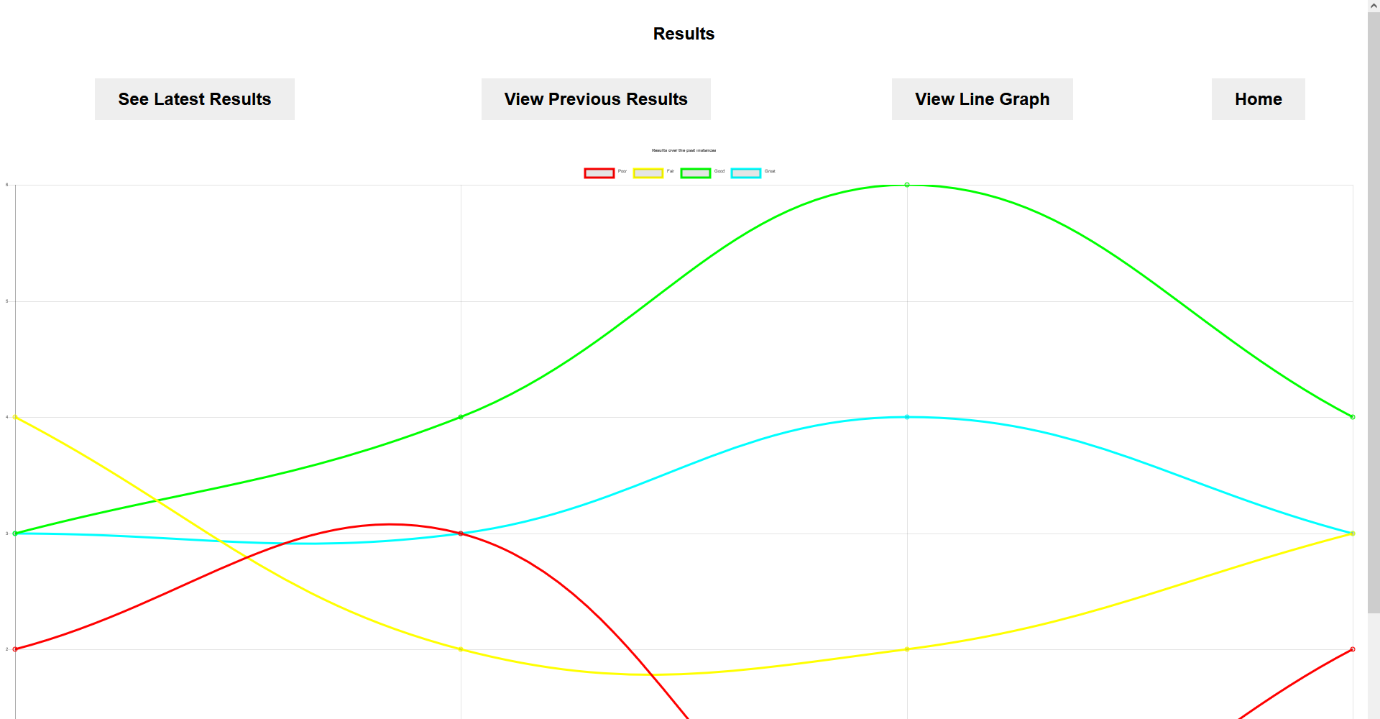
**
